# Catalysis with chalcogen bonds: neutral benzodiselenazole scaffolds with high-precision selenium donors of variable strength†
†Electronic supplementary information (ESI) available: Detailed procedures and results for all reported experiments. CCDC 1568432. For ESI and crystallographic data in CIF or other electronic format see DOI: 10.1039/c7sc03866f


**DOI:** 10.1039/c7sc03866f

**Published:** 2017-10-16

**Authors:** Sebastian Benz, Jiri Mareda, Céline Besnard, Naomi Sakai, Stefan Matile

**Affiliations:** a Department of Organic Chemistry , University of Geneva , Geneva , Switzerland . stefan.matile@unige.ch ; http://www.unige.ch/sciences/chiorg/matile/ ; Tel: +41 22 379 6523

## Abstract

The benzodiselenazoles (BDS) introduced in this report fulfill, for the first time, all the prerequisites for non-covalent high-precision chalcogen-bonding catalysis in the focal point of conformationally immobilized σ holes on strong selenium donors in a neutral scaffold. Rational bite-angle adjustment to the long Se–C bonds was the key for BDS design. For the unprecedented BDS motif, synthesis of 12 analogs from *o*-xylene, crystal structure, σ hole variation strategies, optoelectronic properties, theoretical and experimental anion binding as well as catalytic activity are reported. Chloride binding increases with the depth of the σ holes down to *K*_D_ = 11 μM in THF. Catalytic activities follow the same trend and culminate in rate enhancements for transfer hydrogenation of quinolines beyond 100 000.

## Abstract 2

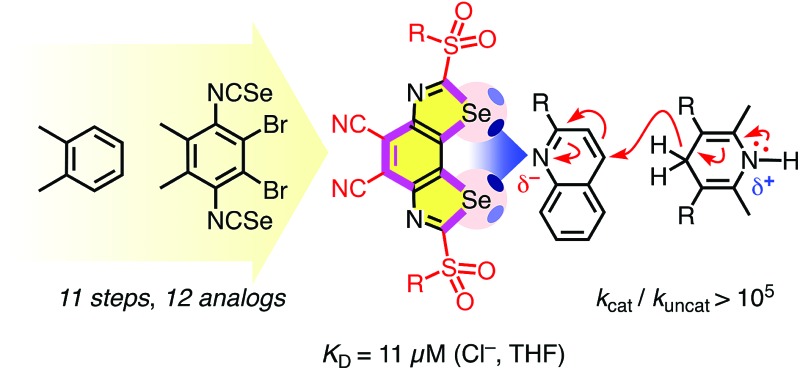
Benzodiselenazoles are introduced for efficient anion binding and unprecedented non-covalent catalysis in the focal point of neutral selenium-based chalcogen-bond donors.

## 

The integration of new interactions into functional systems is an objective of highest, most fundamental importance.^1^–^7^ It expands our ability to create function and promises access to new properties. In organocatalysis, a renewed interest in the integration of conventional interactions such as dispersion forces,^3^ ion pairing^4^ or cation–π interactions^5^ accounts for much recent progress in the field. Catalysis with halogen bonds^2^,^6^ and anion–π interactions,^7^ the unorthodox counterparts of hydrogen bonds and cation–π interactions, have been introduced recently to catalysis. The youngest in this series, chalcogen bonds at work in non-covalent catalysis have been reported earlier this year.^8^,^9^

Chalcogen bonds originate from the σ holes on electron-deficient sulfur, selenium, tellurium but not oxygen atoms.^10^ Produced by the anti-bonding σ* orbitals, the two σ holes locate in plane with the two covalent bonds (Fig. 1). Their sideward, somewhat hidden position has limited attention to solid state engineering and intramolecular covalent conformational control, also in covalent catalysis.^1^,^10^–^12^ The appearance of intermolecular chalcogen bonds in non-covalent supramolecular systems is relatively rare and recent. Leading examples include macrocycles^13^ and rotaxanes,^14^ anion binding,^14^,^15^ anion transport^16^ and mechanosensitive probes.^17^

**Fig. 1:**
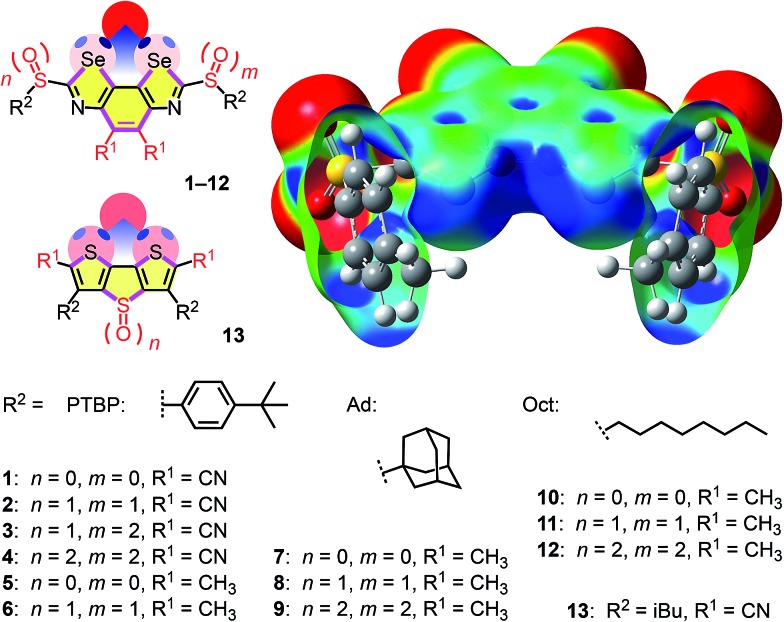
The BDS (top, **1–12**) and DTT motif (bottom, **13**) with electron-rich chalcogen-bond acceptors (red) bound in the focal point of the σ holes (blue), together with the semitransparent cutaway molecular electrostatic potential (MEP) surface of **4′** (R^2^ = pMePh, MP2/6-311++G**//M062X/6-311G**, isosurface: 0.008 au; red: –0.010 au, blue: 0.096 au).^22^

This year's first two examples for non-covalent catalysis with chalcogen bonds focus on dithieno[3,2-*b*:2′,3′-*d*]-thiophenes (DTTs)^8^ and bis(2-selanylbenzimidazolium)s.^9^ DTTs are attractive to stabilize anionic transition states with high precision in the focal point of the two cofacial σ holes (Fig. 1),^8^ but they are limited to weak sulfur donors. Bis(2-selanylbenzimidazolium)s, used in stoichiometric amounts, provide access to more powerful selenium donors but suffer from lack of precision due to conformational flexibility and a dicationic scaffold that obscures contributions from chalcogen bonding and adds complications from counterions.^9^ To overcome these problems, we here introduce benzodiselenazoles (BDS) as an unprecedented structural motif that unifies powerful selenium donors with high-precision chalcogen bonding in the focal point of conformationally immobilized σ holes of variable strength (*i.e.***1–12**, Fig. 1).

Bite or bent angle adjustment is of utmost importance in supramolecular systems.^18^ Determined by the orientation of the antibonding σ* orbital, Se–Cl chalcogen bonding, highly directional, is strongest with a C–Se–Cl bond angle of 180°. For chalcogen bonding in the focal point of the two donors in scaffolds derived from 2,2′-biselenophene or -thiophene, the ideal Cl–Se–Se or Cl–S–S bite angle is thus around 45° (Fig. 2*). Smaller and larger angles move the focal point too close and too far away from the donors, respectively, resulting in a formal outward and inward bending of the chalcogen bonds, *i.e.*, weak binding.

**Fig. 2:**
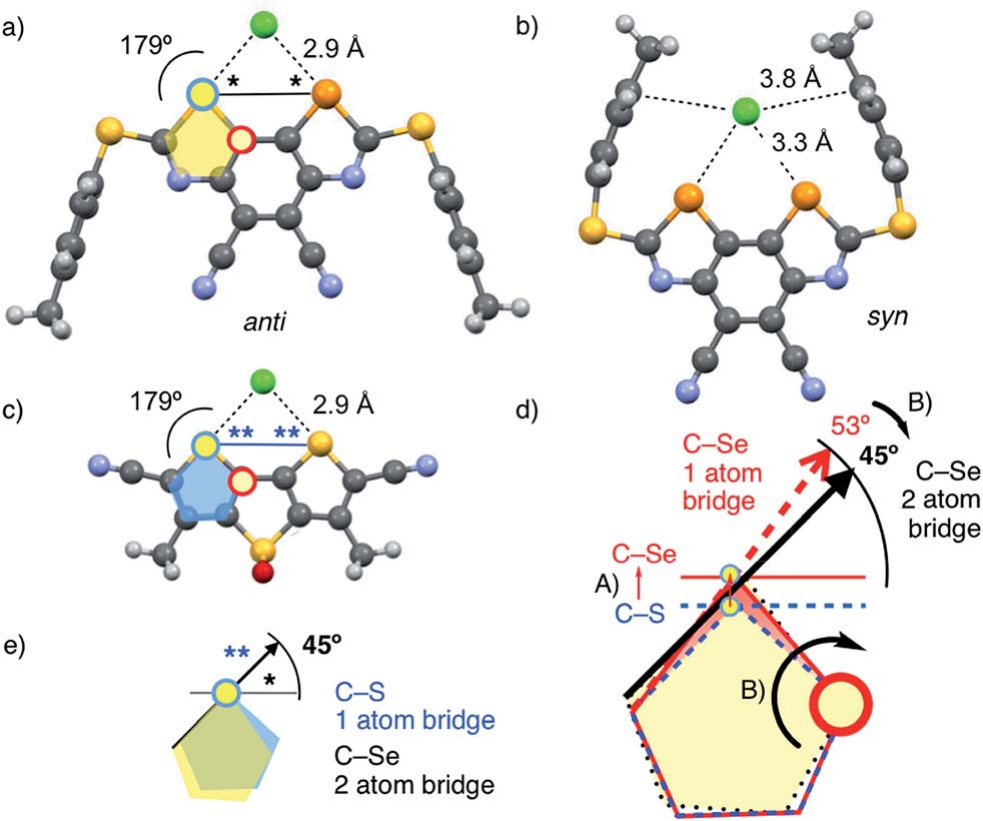
(a, b) DFT-M062X/6-311G** models of chloride (green) bound to BDS **1′** (R^2^ = pMePh) in (a) *anti* and (b) *syn* conformation of PTBP sulfide substituents. (c) Same for DTT **13′** (R^2^ = Me). (d) Overlay of DTT (dashed, blue, c), “DSeT” (red, solid) and BDS (dotted, black, b) on their C2 carbon (red circle). (A) Shift of chalcogen atom from DTT to “DSeT”. (B) Change of bite angle from “DSeT” (53°) to BDS (*, 45°) by inward rotation around C2. (e) Overlay of DTT (blue, c) and BDS (yellow, a) on their chalcogen atom (blue circle).

DTTs such as **13** offer an ideal Cl–S–S bite angle of 45° for the weaker sulfur donors (Fig. 2c**).^8^,^16^ As a result, chalcogen bonds to chloride ions are short and co-linear with the C–S bond. In simple dithiophenes, bite angles are too small (34°), resulting in longer bonds (3.4 Å) with incorrect bond angles (149°).^8^,^16^ The single-atom sulfur bridge in DTTs caused the outward rotation of the peripheral thiophenes that was needed to adjust the bite angle.

Because of the length of the C–Se bond, a formal sulfur–selenium exchange from DTTs to “DSeTs” would increase the bite angle to 53° (Fig. 2d-A, red arrow). This enlarged bite angle would move the focal point away from the Se donors and thus either stretch or bend, *i.e.*, weaken the chalcogen bond. To readjust the bite angle, an inward rotation of the selenophenes was required (Fig. 2d-B). The replacement of the single-atom sulfur bridge in the hypothetical “DSeT” by a double-atom carbon bridge in the BDS scaffold brought the bite angle back to the desired 45° (Fig. 2d-B, black arrows; Fig. 2e*). In computed BDS-chloride complexes of **1′**, the resulting bond angles were correct, and the bond length as short as in DTTs despite the large radius of selenium and the presence of sulfide donors rather than sulfone acceptors (Fig. 2a *vs.*c).

Benzo[1,2-*d*:4,3-*d*′]di([1,3]selenazole)s have not been reported previously. However, similar structures have been explored, mainly for materials applications,^19^ and BDS synthesis could be extrapolated from the known benzoselenazoles.^20^ The most active catalyst **4** was constructed from *ortho*-xylene **14** (Scheme 1). Bromination gave the tetrasubstituted benzene **15**, nitration the fully substituted benzene **16**. Classical transformations afforded diamine **17** by reduction, amide **18** with formic anhydride, and isocyanate **19** by dehydration with phosphoryl chloride, all in good yield. Incorporation of selenium gave isoselenocyanate **20**. Loosely reminiscent of the Edman degradation,^21^ the cyclization into the BDS tricycle **5** was initiated by reacting isoselenocyanate **20** with *p-tert*-butyl-phenyl (PTBP) thiol in the presence of NaH at 0 °C. The resulting selenothiocarbamate intermediate was treated with a catalytic amount of CuI and 1,10-phenanthroline to close the cycles by triggering the formation of the Se–C bond.

**Scheme 1:**
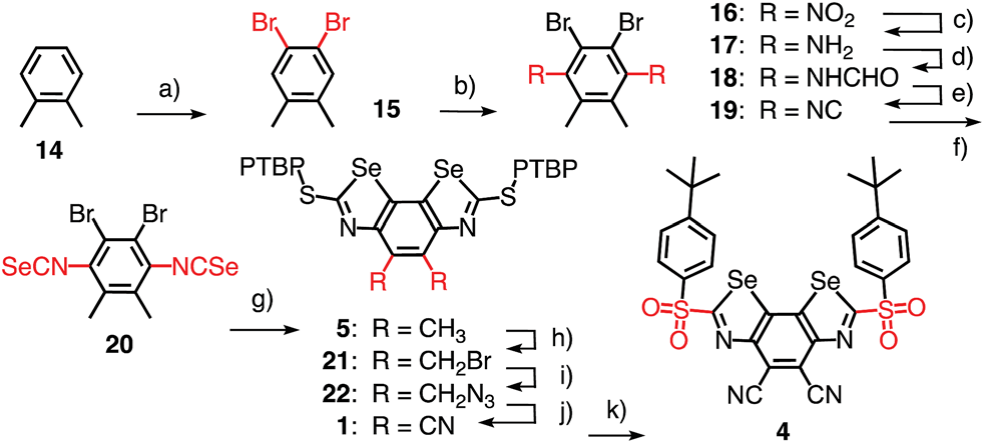
(a) Br_2_, I_2_, neat, rt, 10 h, 52%; (b) HNO_3_/H_2_SO_4_, rt, 8 h, quant; (c) Fe, AcOH/EtOH, reflux, 1 h, 55%; (d) 1. HCOOH/Ac_2_O, 40 °C, 1 h; 2. **17**, 0 °C to rt, 3 h, 57%; (e) Et_3_N, CH_2_Cl_2_, POCl_3_, rt, 4 h, 90%; (f) Se, Et_3_N, CHCl_3_, 90 °C, 14 h, quant; (g) 1. NaH, PTBP-SH, THF, 0 °C, 30 min, 66%; 2. CuI, 1,10-phenanthroline, Cs_2_CO_3_, DME, reflux, 2 h, 45%; (h) NBS, AIBN, DCE, reflux, 2.5 h, 75%; (i) NaN_3_, THF/DMSO, rt, 14 h, quant; (j) DDQ, DCE, 150 °C, μW, 1 h, 30%; (k) mCPBA, CH_2_Cl_2_, rt, 4 h, 45%.

The acceptors on the C_2_ bridge were installed by bromination of BDS **5** with NBS and transformation of the obtained dibromide **21** over diazide **22** into dicyanide **1**. The crystal structure of BDS **1** confirmed the structure of this new motif and provided with CHCl_3_ in the focal point of the σ holes the first indications of powerful chalcogen bonding (Fig. 3a). Finally, the sulfide donors in **1** were oxidized to sulfoxide and sulfone acceptors.

**Fig. 3:**
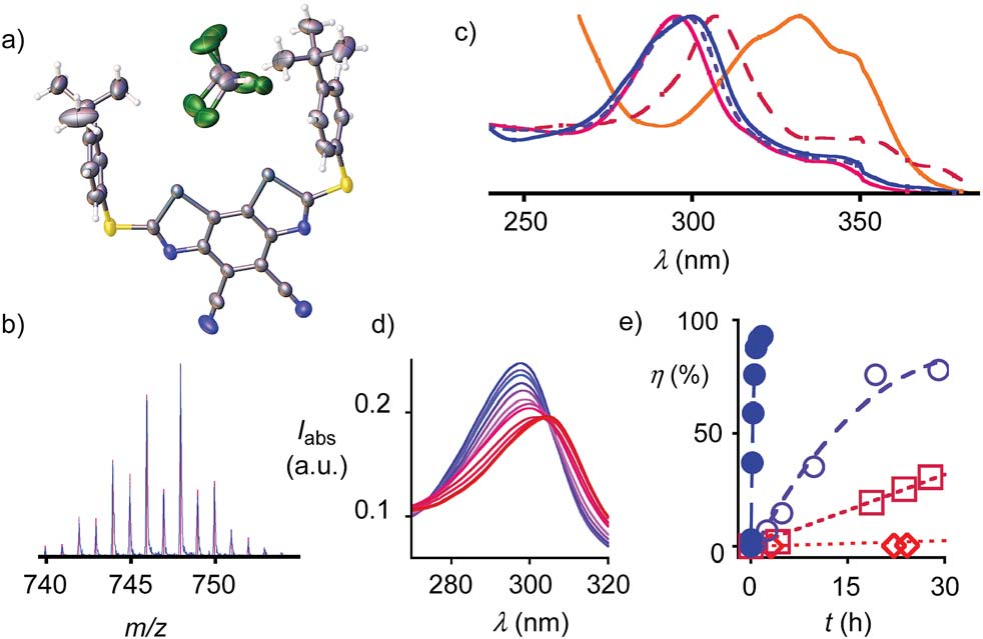
(a) Crystal structure of **1** with CHCl_3_. (b) Simulated (pink) and measured ESI mass spectrum of **4** (blue). (c) Normalized absorption spectra of **2**, **3**, **4**, **1** and **5** in THF (increasing *λ*_max_). (d) Absorption spectra of **2** in THF with increasing concentrations of TBACl (0 to 1.95 mM, blue to red). (e) Conversion *η* of **23a** with 30 mol% **1** (), **2** (□), **3** (○) and **4** () as a function of time, with trend lines.

Catalysts with octyl and adamantly substituents as R^2^ were prepared analogously using the corresponding thiols for cyclization (Fig. 1)^22^ to complete the catalyst collection **1–12**. All selenium-containing products showed the characteristic isotope distribution in the mass (Fig. 3b) and signals in the ^77^Se NMR spectra.^22^

The electron-rich BDS **5** absorbed at *λ*_max_ = 332 nm (Fig. 3c, Table 1, entry 1). The cyano acceptors in **1** and sulfoxides in **2** gradually blue-shifted this maximum down to *λ*_max_ = 295 nm. Further oxidation to sulfones in **3** and **4** shifted it back up to *λ*_max_ = 300 nm. Cyclic voltammograms showed a semi-reversible reduction wave (Fig. S6†) with LUMO energies decreasing with withdrawing substituents down to –3.81 eV for **4** (Table 1, entry 6).

**Table 1:** Characteristics of benzodiselenazoles

Entry	Cpd^*a*^	R^1^^*a*^	*n* ^*a*^	*m* ^*a*^	R^2^^*a*^	*k*_cat_/*k*_uncat_^*b*^	Δ*E*_a_^*c*^ (kJ mol^–1^)	*η* ^*d*^ (%)	*E*_int_ ^*e*^ (kcal mol^–1^)	*K*_D_ ^*f*^ (μM)	*E*_LUMO_ ^*g*^ (eV)	*λ*_max_ ^*h*^ (nm)	*ε* ^*i*^ (mM^–1^ cm^–1^)
1	**5**	CH_3_	0	0	PTBP	∼10	—	n.d	–25.7	n.d.	n.d.	332	27.2
2	**6**	CH_3_	1	1	PTBP	660	–15.8	93	–34.1				
3	**1**	CN	0	0	PTBP	100	–11.3	n.d.	–37.2 (–31.5)^*j*^	n.d.	–3.21	307	79.1
4	**2**	CN	1	1	PTBP	970 (500)^*k*^	–16.8	78	–45.2 (–34.3)^*j*^	530 ± 90	–3.54	295	62.5
5	**3**	CN	1	2	PTBP	3200	–19.6	88	–49.2 (–38.0)^*j*^	37 ± 6	–3.74	298	69.5
6	**4**	CN	2	2	PTBP	150000	–29.1	93^*l*^	–53.0 (–41.6)^*j*^	11 ± 2	–3.81	300	60.3
7	**7**	CH_3_	0	0	Ad	∼10	—	n.d.	n.d.				
8	**8**	CH_3_	1	1	Ad	3100	–19.6	97	n.d.				
9	**9**	CH_3_	2	2	Ad	300	–13.9	47	n.d.				
10	**13**	CN	2	—	iBu	490^*m*^	–15.3^*m*^	96^*m*^	–34.6^*m*^	1130 ± 30^*m*^	–3.70^*m*^	376^*m*^	18.3

^*a*^Compounds, see Fig. 1; *n*, *m*: number of oxygens bound to sulfur, 0 = sulfide, 1 = sulfoxide, 2 = sulfone.

^*b*^Rate enhancement for product formation from **23a** (128 mM) and **24** (281 mM) in CD_2_Cl_2_ at 20 °C with 30 mol% catalyst **1–13**, compared to *k*_uncat_ = 3.9 × 10^–5^ M^–1^ h^–1^.

^*c*^Change in activation energy, from *k*_cat_/*k*_uncat_.

^*d*^Yields determined by ^1^H NMR signal integration.

^*e*^Computed (M062X/6-311G**) chloride binding energy (gas phase, entry 1–6: R^2^ = pMePh).

^*f*^Dissociation constant for TBACl in THF.

^*g*^LUMO energy, in eV against –5.1 eV for Fc^+^/Fc.

^*h*^Absorption maximum in THF.

^*i*^Extinction coefficient at *λ*_max_.

^*j*^ *syn* (*anti*) conformer.

^*k*^Data obtained for a chiral (and the *meso*) diastereomer.

^*l*^Same yield with reduced catalyst loading of 1 mol%.

^*m*^Data from ref. 8. n.d., not determined.^22^

Chloride binding in THF was detectable by UV absorption spectroscopy (Fig. 3d). The obtained *K*_D_'s decreased with increasing depth of the σ holes down to an outstanding *K*_D_ = 11 ± 2 μM for BDS **4** (Table 1, entry 6). They exceeded anion recognition with the best comparable DTT **13** by two orders of magnitude (*K*_D_ = 1130 ± 30 μM) and corresponded well with computed interaction energies *E*_int_ (Table 1).

In computational models of **1**, chloride binding with the PTBP sulfide substituents in *syn* conformation was preferred by 5.7 kcal mol^–1^ (Fig. 2b and Table 1, entry 3). The *anti* conformer shows shorter chalcogen bonds with perfect bond angles (Fig. 2a). However, the *syn* conformer contains additional, very weak (Cl–C_Ph_ ≥ 3.8 Å) anion–π interactions^7^ to the π-basic phenyls that move the chloride away from the focal point of the σ holes, thus elongating the chalcogen bonds and decreasing the angles (Fig. 2b).

Contrary to the crystal structure of **1** with CHCl_3_ instead of Cl^–^ (Fig. 3a), the phenyl rings in the computed chloride complex of **1′** bend inward to catch the chloride anion in a tweezer-like motion (Fig. 2b). The result is a nicely preorganized, dynamic binding pocket with an attractive combination of (weak) anion–π interactions and (strong) chalcogen bonds that reflects the found strong anion binding very well. In models with molecular electrostatic potential (MEP) surfaces, catalyst **4′** appeared like a molecular crab, with the mouth represented by the σ holes nicely visible on selenium donors that look like eyes, and the phenyl substituents reminiscent of the flexible claws (Fig. 1).

In the *anti* conformer of disulfoxide **2′**, competition from intramolecular chalcogen bonds to sulfoxide oxygens further weakened anion binding (Fig. S8†). As a result, the preference to the *syn* conformers increased to 10.9 kcal mol^–1^ for sulfoxide **2′** (Table 1, entry 4). Inactivation by intramolecular chalcogen bonds might contribute to the comparably small increase in activity from sulfides **1** to sulfoxides **2** (binding and catalysis, below, Table 1, entries 3, 4).

Taken together, crystal structures (Fig. 3a), neutral, green MEPs on the aromatic surface of the BDS (Fig. 1),^23^,^24^ LUMO levels at –3.81 eV or higher (Table 1),^23^ and the structure of minimized computational models of chloride complexes (Fig. 2, S7 and S8†) firmly excluded significant contributions from anion–π interactions between anions and BDS. The same crystal structure and minimized chloride complexes, and the highly positive, deep blue MEP surface toward the focal point of the σ holes of the BDS (*i.e.*, the mouth of the molecular crab, Fig. 1) provided compelling support for operational chalcogen bonding. Experimental and theoretical evidence for strengthened chloride binding by deepened σ holes confirmed the validity of this conclusion. For instance, the weak sulfide donors in BDS **1** produced shallow σ holes on the Se donors, which resulted in undetectable chloride binding in THF and computed interaction energies for chloride complexes of maximal *E*_int_ = –37.2 kcal mol^–1^ (Table 1, entry 3). Sulfoxide acceptors in BDS **2** produced deeper σ holes, detectable chloride binding in THF with *K*_D_ = 530 ± 90 μM and stronger *E*_int_ = –45.2 kcal mol^–1^ in computed chloride complexes (Table 1, entry 4). One even stronger sulfone acceptor in BDS **3** further deepened the σ holes and increased chloride binding in THF to *K*_D_ = 37 ± 6 μM and *in silico* to *E*_int_ = –49.2 kcal mol^–1^ (Table 1, entry 5). The deepest σ holes of the series in BDS **4** with two strong sulfone and two strong cyano acceptors, finally, coincided with the strongest chloride binding in THF and in computed complexes (*K*_D_ = 11 ± 2 μM, *E*_int_ = –53.0 kcal mol^–1^, Table 1, entry 6).

Evidence for efficient anion stabilization in the ground state implied that chalcogen bonding with BDS could also stabilize anionic transition states in the focal point of the σ holes of their Se donors. Previous computational studies have indicated that neutral lone pairs extending from the endocyclic aromatic nitrogen in the substrate **23** are already well recognized even with weak sulfur donors.^8^ This chalcogen bonding should increase with increasing negative charge accumulation on the nitrogen atom, that is stabilize the transition states of the nucleophilic addition to nitrogen-containing aromatic heterocycles. For transfer hydrogenation of quinolines **23**, the negative charge injected by the hydride should be attracted to and, in the transition state, be located on the endocyclic nitrogen (Fig. 4b). This would enable transition-state stabilization in the focal point of the σ holes of BDS, and hence could result in rate enhancement of the transfer hydrogenation, *i.e.*, catalysis with chalcogen bonds.

**Fig. 4:**
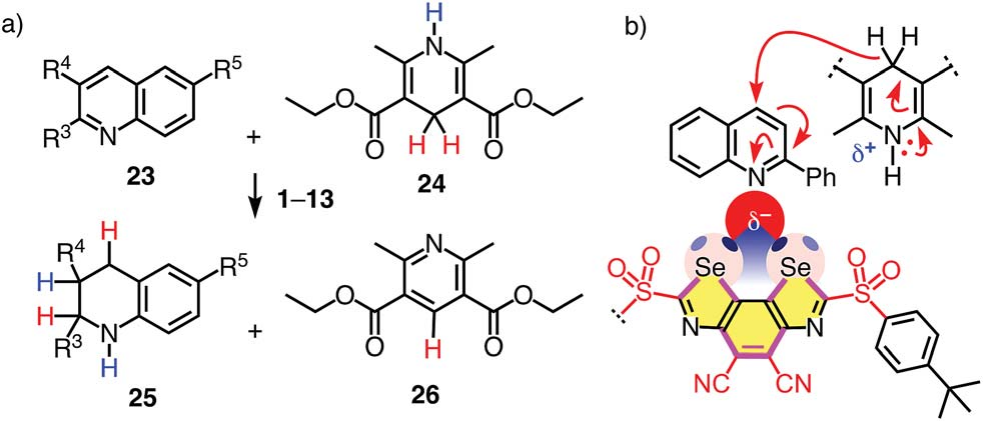
(a) Transfer hydrogenation of quinolines **23a–h** (R^3^–R^5^: Table 2) with catalysts **1–13** and (b) the expected transition-state stabilization by chalcogen bonding, exemplified for substrate **23a** and catalyst **4**.

Without catalyst, the transfer hydrogenation of quinoline **23a** with Hantzsch ester **24** affords tetrahydroquinoline **25a** and pyridine **26** with *k*_uncat_ = 3.9 × 10^–5^ M^–1^ h^–1^ (Fig. 4a).^6^,^8^ With donating substituents, BDS such as **5**, **7** and **10** were as inactive as expected for poor chalcogen bonding to the shallow σ holes of electron-rich systems (Table 1, entries 1, 7, Table S1†). Oxidation of sulfide donors to sulfoxide acceptors in **6**, **8** and **11** and sulfones in **9** and **12** gradually deepened the σ holes and turned on catalytic activity (Table S1†). Surprisingly high activities found for BDS **8** with adamantyl substituents on the sulfoxide level did not extrapolate to further increases with sulfone **9** (Table 1, entries 8, 9).

Replacement of the methyl donors by cyano acceptors in the C_2_ bridge to deepen the σ holes on the Se donors produced detectable rate enhancements *k*_cat_/*k*_uncat_ = 100 already with sulfide BDS **1** (Table 1, entry 3). Upon replacement of the sulfide donors by chiral sulfoxide acceptors in BDS **2**, activities increased up to *k*_cat_/*k*_uncat_ = 970 as expected for stronger chalcogen bonding by deepened σ holes on the Se donors (Fig. 3e□ and Table 1, entry 4). Further rate enhancements up to *k*_cat_/*k*_uncat_ = 3200 upon adding one strong sulfone acceptor in BDS **3** were consistent with deepened σ holes and strengthened chloride binding in THF and in computed chloride complexes (Fig. 3e○ and Table 1, entry 5). Finally, the highest rate enhancement of more than a hundred thousand was achieved, as expected, by BDS **4** with maximized σ holes by two strong sulfone and two strong cyano acceptors and is consistent with equally maximized chloride binding in solution and *in silico* (Fig. 3e and Table 1, entry 6). The best BDS catalyst **4** was thus more than two orders of magnitude more active than the best comparable DTT **13** (Table 1, entries 6, 10; DTT diimides: *k*_cat_/*k*_uncat_ = 1290).^8^ These consistent trends demonstrated that transition-state stabilization by chalcogen bonds in the focal point of neutral selenium donors of maximized strength (Fig. 4b) exceeds that by equally activated but weaker sulfur donors by far.

The *meso* diastereomer of sulfoxide **2** was markedly less active than its chiral counterpart (Table 1, entry 4). This diastereoselectivity confirmed that the location of both PTBP substituents on the same side of the aromatic plane (Fig. S8†) hinders chalcogen-bond activation of the substrate. The higher activity of the chiral diastereomer of sulfoxide **2** with the PTBP substituents on opposite sides of the BDS plane (Fig. S8†) suggested that the binding of the nitrogen lone pair in the focal point of the chalcogen-bond donors can occur with an angle between the quinoline and BDS planes that is <90° (Fig. 4). This likely twist in a chiral environment was however insufficient for asymmetric catalysis: enantiopure sulfoxide catalysts **2**, isolated by chiral HPLC, failed to produce significant enantioselectivity (not shown).

Having identified **4** as a potent chalcogen bonding catalyst, catalyst loading was successfully reduced to 1 mol%, which furnished the product **25a** in identical yield of 93% after 24 h reaction time (Table 1, entry 6). Under these conditions, transfer hydrogenation of a variety of substituted and unsubstituted quinolines was explored (Table 2 and Fig. 4). Both electron-rich (Table 2, entries 2, 8) and electron-poor quinolines (Table 2, entries 1, 6) were smoothly reduced. Substrates with methoxy donors were naturally less reactive (Table 2, entry 7). Substrates with chloride and nitro acceptors were essentially not converted (Table 2, entries 3, 4). This result suggested that the electron density on the nitrogen is insufficient to bind to the σ holes in the catalyst, and chalcogen bonding to the acceptors might be preferred. Catalyst **4** was further confirmed to catalyze the imine reduction in *N*-benzylidene-aniline with Hantzsch ester **24** (Scheme S6†). The amine product was obtained in quantitative yield.

**Table 2:** Substrate screening

Entry	S^*a*^	R^3^	R^4^	R^5^	*t* ^*b*^ (h)	*η* ^*c*^ (%)
1	**23a**	Ph	H	H	24	93
2	**23b**	H	H	H	24	89 (98)^*d*^
3	**23c**	Cl	H	H	48	0
4	**23d**	H	H	NO_2_	48	9
5	**23e**	Me	H	F	24	80 (88)^*d*^
6	**23f**	H	Br	H	24	98
7	**23g**	H	H	OMe	24	32 (58)^*d*^
8	**23h**	H	H	Me	24	97

^*a*^Substrates, see Fig. 4.

^*b*^Reaction time with **23a–h** (128 mM), **24** (281 mM) and 1 mol% **4** in CD_2_Cl_2_ at 20 °C.

^*c*^Yield of the reduced product, determined by ^1^H NMR signal integration against internal standard.

^*d*^Yields in brackets determined after 48 h reaction time.

Compared to hydrogen-bonding catalysis, chalcogen-bonding catalysis is expected to excel with unique directionality, that is highest precision, particularly in hydrophobic environments. In the new BDS scaffold, this directionality is maximized. Synthesized from *ortho*-xylene, the best BDS binds chloride with low micromolar *K*_D_'s in THF and enhances the rate of transfer hydrogenation by five orders of magnitude. Increasing activities with deepening σ holes are consistent with powerful chalcogen bonds at work (Table 1, clearest for entries 3–6) and encourage further development of the concept, particularly with regard to asymmetric catalysis and the integration into more complex systems.^6^,^16^,17b,^25^

## Conflicts of interest

There are no conflicts of interest to declare.

## Supplementary Material

Supplementary information

Crystal structure data
