# Supplementary material for: Catalysis with chalcogen bonds: neutral benzodiselenazole scaffolds with high-precision selenium donors of variable strength
Source: Chem Sci. 2017 Oct 16;8(12):8164–9. doi: 10.1039/c7sc03866f (PMC5855965; doi:10.1039/c7sc03866f)
Supplement: Supplementary file 1 [file SC-008-C7SC03866F-s001.pdf]

Electronic Supplementary Material (ESI) for Chemical Science.
This journal is © The Royal Society of Chemistry 2017

Supplementary Information

Catalysis with chalcogen bonds: neutral benzodiselenazole scaffolds with high-precision selenium donors of variable strength

Sebastian Benz, Jiri Mareda, Céline Besnard, Naomi Sakai, and Stefan Matile *

Department of Organic Chemistry, University of Geneva, Geneva, Switzerland

stefan.matile@unige.ch

Table of content

1. Materials and methods S2
2. Synthesis S3
3. Catalyst evaluation S17
   3.1. Rate studies S17
   3.2. Substrate scope S21
4. Anion binding S22
5. Electrochemistry S25
6. Computational studies S27
7. Crystallographic data S29
8. Supplementary references S31
9. HRMS spectra S33
10. NMR spectra S41

1. Materials and methods

As in refs. S1 and S2, Supporting Information. Reagents for synthesis were purchased from Fluka, Sigma-Aldrich, Apollo Scientific and Acros. Column chromatography was carried out on silica gel (SiliaFlash® P60, SILICYCLE, 230–400 mesh). Analytical (TLC) and preparative thin layer chromatography (PTLC) were performed on silica gel 60 F254 (Merck) and silica gel (SiliCycle, 1000 µm), respectively. Microwave reactions were performed with a CEM Discover reactor. IR spectra were recorded on a Perkin Elmer Spectrum One FT-IR spectrometer (ATR, Golden Gate) and are reported as wavenumbers v in cm^{-1} with band intensities indicated as s (strong), m (medium), w (weak). ^{1}H and ^{13}C NMR spectra were recorded (as indicated) either on a Bruker 300 MHz, 400 MHz or 500 MHz spectrometer and are reported as chemical shifts (δ) in ppm relative to TMS (δ= 0). ^{77}Se NMR spectra were recorded on a Bruker 300 MHz spectrometer. Spin multiplicities are reported as a singlet (s), doublet (d), triplet (t), quartet (q) and nonet (non), with coupling constants (J) given in Hz, or multiplet (m). Broad peaks are marked as br. ^{1}H and ^{13}C resonances were assigned with the aid of additional information from 1D and 2D NMR spectra (H,H-COSY, DEPT 135, HSQC and HMBC). ESI-MS was performed on an ESI API 150EX and are reported as mass-per-charge ratio m/z (intensity in %, [assignment]). Only the most intense ones among isotopic peaks are reported. HR ESI-MS for the characterization of new compounds were performed on a QSTAR Pulsar (AB/MDS Sciex) and are reported as mass-per-charge ratio m/z calculated and observed. Electrochemical measurements were done on an Electrochemical Analyzer with Picoamp booster and Faraday cage (CH Instruments 660C). UV-Vis spectra were recorded on a JASCO V-650 spectrophotometer equipped with a temperature controller.

Abbreviations. AcOH: Acetic acid, AIBN: 2,2′-Azobis(2-methylpropionitrile), BDS: Benzodiselenazoles, CV: Cyclic Voltammetry, DCE: 1,2-Dichloroethane, DDQ: 2,3-Dichloro-

5,6-dicyano-*p*-benzoquinone, DME: 1,2-Dimethoxyethane, DMSO; Dimethyl sulfoxide, mCPBA: 3-Chloroperbenzoic acid; NBS: *N*-Bromosuccinimide, PE: Petroleum ether; PTBP: *p*-*tert*-butyl-phenyl; rt: Room temperature; SCE: Saturated calomel electrode, TBACl: Tetrabutylammonium chloride, THF: Tetrahydrofuran, µW: Microwave reactor.

2. Synthesis

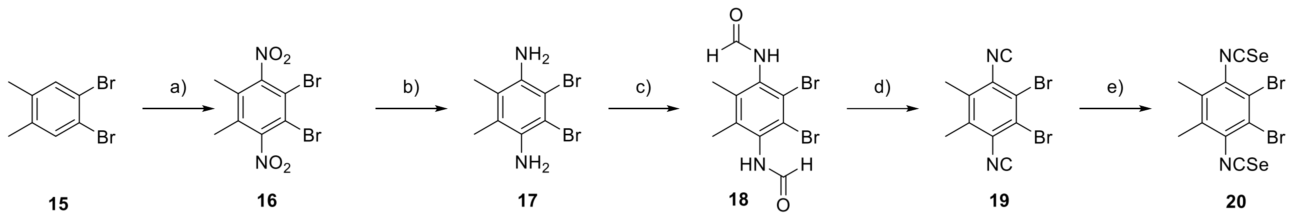

Scheme S1. Reagents and conditions: (a) HNO_3/H_2SO_4, rt, 8 h, quant; (b) Fe, AcOH/EtOH, reflux, 1 h, 55%; (c) 1. HCOOH/Ac_2O, 40 °C, 1 h. 2. **17**, 0 °C to rt, 3 h, 57%; (d) Et_3N, CH_2Cl_2, $POCl_3$, rt, 4 h, 90%; (e) Se, Et_3N, $CHCl_3$, 90 °C, 14 h, quant.

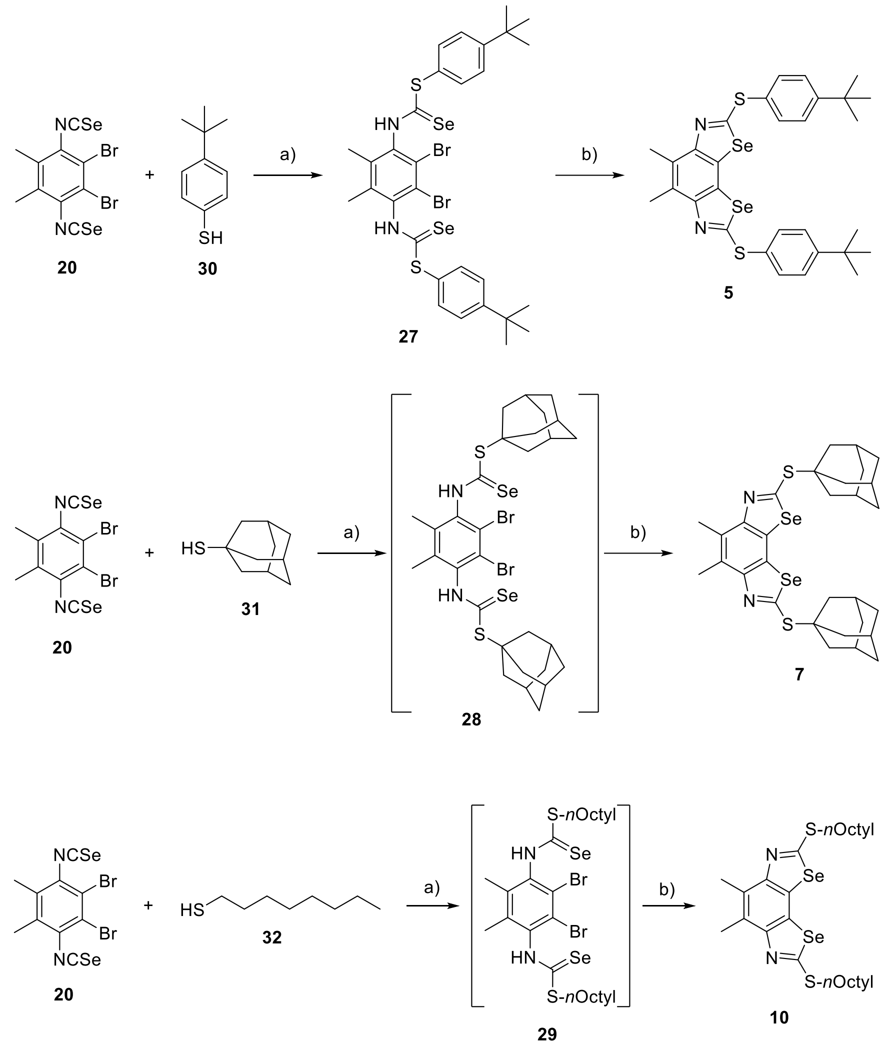

Scheme S2. Reagents and conditions: (a) NaH, THF, 0 °C, 30 min, quant; (b) CuI, 1,10-Phenanthroline, Cs_2CO_3, DME, reflux, 2 h, **5**: 68%, **7**: 58%, **10**: 60%.

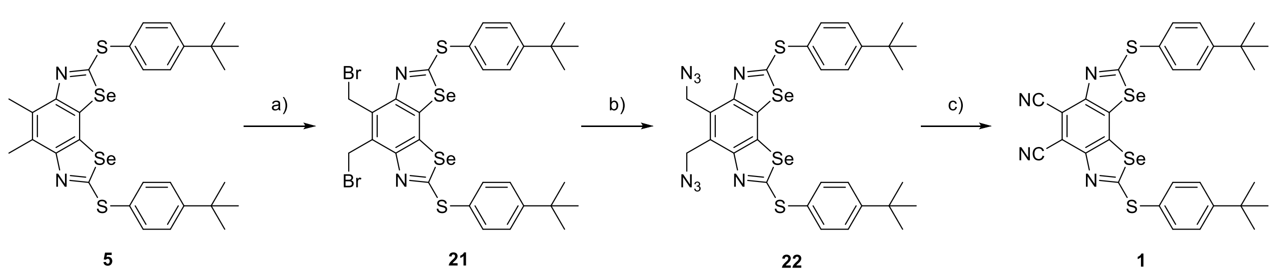

Scheme S3. (a) NBS, AIBN, DCE, reflux, 2.5 h, 75%; (b) NaN_3, THF/DMSO, rt, 14 h, quant; (c) DDQ, DCE, 150 °C, µW, 1 h, 30%.

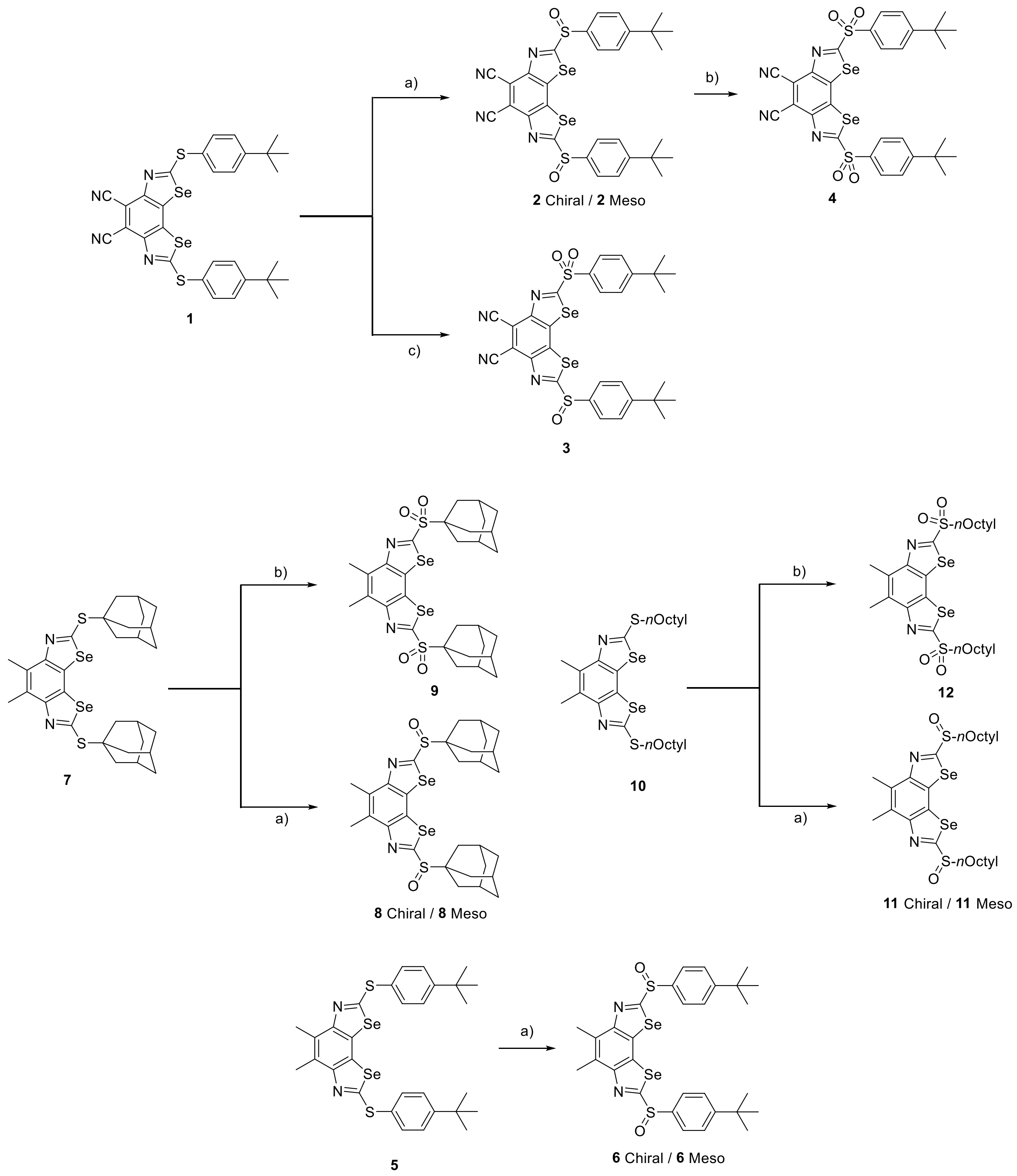

Scheme S4. Reagents and conditions: (a) 3 eq. mCPBA, CH_2Cl_2, rt, 30 min, 5 h, **2 Chiral**: 35%, **2 Meso**: 32%;1 h, **6 Chiral**: 20%, **6 Meso**: 12%; 30 min, **8 Chiral**: 30%, **8 Meso**: 45%; 2 h, **11 Chiral**: 38%, **11 Meso**: 34%; (b) 10 eq. mCPBA, CH_2Cl_2, rt, 4 h, **4**: 45%, 2 h, **9**: 46%, 1 h, **12**: 25%; (c) 3.6 eq. mCPBA, CH_2Cl_2, rt, 1 h, 15%.

Compound 15. was synthesized according to the procedure reported in reference S3.

Compound 16. To sulfuric acid (96%, 300 mL), nitric acid (90%, 300 mL) was added slowly. Then **3** (60.0 g, 227 mmol) was slowly introduced as a fine powder to this mixture and the resulting solution was stirred for 8 h at 50 °C. After cooling to rt the reaction mixture was poured on ice water, filtered and washed with water. Drying under high vacuum gave **16** as a colorless solid (80.4 g, quant), containing about 15% mono nitrated byproduct as impurity. This crude product was used without further purification in the next step. R_f (PE/EtOAc 9:1): 0.5; IR (neat): 2979 (w), 2901 (w), 1732 (m), 1537 (w), 1374 (m), 1242 (s), 1044 (s), 913 (m), 731 (s); ^{1}H NMR (400 MHz, $CDCl_3$): 2.3 (s, 6H); ^{13}C NMR (101 MHz, $CDCl_3$): 153.1 (C), 131.1 (C), 115.4 (C), 15.6 (CH_3); MS (ESI, $CHCl_3$/MeOH): 355 (100, $[M+H]^+$).

Compound 17. To a suspension of **16** (50.0 g, 141 mmol) in acetic acid (125 mL) and ethanol (500 mL) was added iron powder (125 g) and the mixture was refluxed for 1 h. After cooling to rt the reaction mixture was basified with conc. aq. KOH, diluted with DCM and filtered. The filtrate was washed with aq. 1 M KOH and water, the organic phase was dried over Na_2SO_4 and concentrated *in vacuo* to afford **17** as a yellow solid (22.8 g, 55%). R_f (PE/EtOAc 9:1): 0.2; IR (neat): 3400 (w), 3314 (w), 2919 (w), 2861 (w), 1616 (s), 1514 (s), 1453 (m), 1427 (m), 1364 (s), 1277 (w), 914 (w), 725 (w), 708 (w), 666 (w); ^{1}H NMR (400 MHz, $CDCl_3$): 3.85 (br s, 4H), 2.15 (s, 6H); ^{13}C NMR (101 MHz, $CDCl_3$): 136.1 (C), 122.5 (C), 110.3 (C), 15.0 (CH_3); MS (ESI, $CHCl_3$): 295 (100, $[M+H]^+$); HRMS (ESI, +ve) calcd for $C_8H_{10}Br_2N_2$: 292.9284, found: 292.9272.

Compound 18. To acetic anhydride (554 mL) formic acid (426 mL) was slowly added and the resulting solution was stirred for 1 h at 40 °C. After cooling to 0 °C finely ground **17** (43.0 g, 146 mmol) was added and stirred for 3 h at rt. Subsequently the formed precipitate was collected

by filtration, and after removal of the filtrate washed with methanol. Drying *in vacuo* afforded **18** (29.4 g, 57%) as a poorly soluble colorless solid. IR (neat): 2919 (s), 2851 (m), 1567 (w), 1467 (s), 1344 (m), 981 (s), 913 (m), 884 (m), 723 (m); ^{1}H NMR (400 MHz, DMSO-d_6): 9.99 (s, 2H), 8.29 (s, 2H), 2.13 (s, 2H); ^{13}C NMR (101 MHz, DMSO-d_6): 159.7 (CH), 136.2 (C), 134.5 (C), 122.8 (C), 16.4 (CH_3); MS (ESI, $CHCl_3$): 351 (100, $[M+H]^+$); HRMS (ESI, +ve) calcd for $C_{10}H_{10}Br_2N_2O_2$: 348.9182, found: 348.9177.

Compound 19. To a suspension of **18** (29.4 g, 83.8 mmol) in dry CH_2Cl_2 were added Et_3N (94 mL, 670 mmol) followed by the dropwise addition of $POCl_3$ (31.4 mL, 336 mmol). The resulting mixture was stirred under an argon atmosphere at rt for 4 h until all solid was dissolved. The reaction mixture was basified by the careful addition of sat. aq. K_2CO_3, diluted with CH_2Cl_2 and washed with water. The organic phase was dried over Na_2SO_4 and filtered through a 25 cm thick pad of silica, which was washed with CH_2Cl_2. The filtrate was concentrated *in vacuo* to yield **19** (23.6 g, 90%) as a colorless solid. R_f (PE/EtOAc 9:1): 0.5; IR (neat): 2927 (w), 2855 (w), 2115 (s), 1665 (w), 1377 (m), 1262 (m), 1111 (m), 929 (m), 819 (m), 756 (m), 701 (s); ^{1}H NMR (400 MHz, $CDCl_3$): 2.5 (s, 6H); ^{13}C NMR (101 MHz, DMSO-d_6): 173.4 (C), 135.7 (C), 128.6 (C), 121.9 (C), 17.6 (CH_3).

Compound 20. To a solution of **7** (23.0 g, 73.2 mmol) in $CHCl_3$ (500 mL) were added selenium powder (17.4 g, 220 mmol) and Et_3N (31 mL, 22 mmol). Argon was bubbled through the solution for 5 min before the pressure reaction vessel was closed and heated under stirring at 90 °C for 14 h. After cooling to rt the reaction mixture was filtered and the filtrate was extracted with CH_2Cl_2, washed with water, dried over Na_2SO_4 and concentrated *in vacuo* to yield **20** (34.5 g, quant.) as an ocher solid. R_f (PE/EtOAc 9:1): 0.3; IR (neat): 2922 (w), 2048 (s), 1607 (m), 1379

(m), 929 (m); ^{1}H NMR (400 MHz, $CDCl_3$): 2.38 (s, 6H); ^{13}C NMR (101 MHz, $CDCl_3$): 135.2 (C), 134.9 (C), 129.6 (C), 122.7 (C), 17.0 (CH_3).

Compound 27. To a solution of **20** (1.20 g, 2.54 mmol) in dry THF (29 mL) was added **30** (1.1 mL, 6.4 mmol) and argon was bubbled through the solution for 5 min. Subsequently the mixture was cooled to 0 °C and NaH (60% dispersion in mineral oil, 254 mg, 6.36 mmol) was added in portions and the mixture was stirred for 30 min under argon atmosphere. The reaction mixture was neutralized with sat. NH_4Cl, diluted with CH_2Cl_2 and washed with brine. After drying over Na_2SO_4 and concentration *in vacuo* crude product **27** along with excess thiol was obtained, which could be directly used in the next step. Flash column chromatography of the crude (SiO_2, CH_2Cl_2/PE 9:1) afforded pure **27** as a faint yellow solid (1.3 g, 66%). R_f (CH_2Cl_2/PE 3:2): 0.3; IR (neat): 3286 (m), 2957 (m), 2902 (w), 1445 (s), 1397 (m), 1309 (s), 1084 (w), 952 (s), 911 (s), 825 (s), 729 (m), 671 (m), 620 (w); ^{1}H NMR (400 MHz, $CDCl_3$): 8.33 (s, 2H), 7.64 (d, ^{3}J = 8.4 Hz, 4H), 7.59 (d, ^{3}J = 8.4 Hz, 4H), 2.16 (s, 6H), 1.37 (s, 18H); ^{13}C NMR (101 MHz, $CDCl_3$): 198.2 (C), 156.0 (C), 137.9 (C), 135.0 (CH), 128.3 (CH), 125.3 (C), 123.8 (C), 35.3 (C), 31.2 (CH_3), 16.9 (CH_3); ^{77}Se NMR (57 MHz, $CDCl_3$): 609; MS (ESI, $CHCl_3$/MeOH): 806 (100, $[M+H]^+$); HRMS (ESI, +ve) calcd for $C_{30}H_{34}Br_2N_2S_3Se_2$: 804.8961, found: 804.8953.

Compound 5. To a suspension of **27** (10.59 mmol) as a crude mixture in DME (100 mL) were added 1,10-phenanthroline (1.91 g, 10.6 mmol), CuI (1.20 g, 6.35 mmol) and Cs_2CO_3 (6.90 g, 21.2 mmol). The mixture was refluxed under an air balloon for 2 h. After cooling to rt the crude reaction mixture was diluted with CH_2Cl_2 and filtered. The filtrate was washed with water, dried over Na_2SO_4 and concentrated *in vacuo*. Purification by flash column chromatography (SiO_2, CH_2Cl_2/PE 3:2) afforded **5** as a yellow solid (3.16 g, 45%). R_f (CH_2Cl_2/PE 3:2): 0.4; IR (neat): 2958 (m), 2864 (w), 1470 (s), 1391 (w), 1343 (m), 1265 (w), 1115 (w), 995 (s), 917 (m), 886 (m),

831 (s), 675 (w); ^{1}H NMR (400 MHz, $CDCl_3$): 7.66 (d, ^{3}J = 8.4 Hz, 4H), 7.46 (d, ^{3}J = 8.4 Hz, 4H), 2.71 (s, 6H), 1.35 (s, 18H); ^{13}C NMR (101 MHz, $CDCl_3$): 170.6 (C), 154.7 (C), 152.4 (C), 135.5 (CH), 130.3 (C), 129.6 (C), 128.1 (C), 127.2 (CH), 35.1 (C), 31.3 (CH_3), 16.5 (CH_3); ^{77}Se NMR (57 MHz, $CDCl_3$): 711; MS (ESI, $CHCl_3$/MeOH 1:1): 645 (100, $[M+H]^+$); HRMS (ESI, +ve) calcd for $C_{30}H_{32}N_2S_2Se_2$: 645.0410, found: 645.0410.

Compound 21. To a solution of **5** (700 mg, 1.09 mmol) in DCE (42 mL) was added NBS (1.16 g, 6.55 mmol) and AIBN (179 mg, 1.09 mmol) and the resulting mixture was stirred at reflux for 2.5 h. Subsequently the reaction mixture was cooled to rt, silica gel was added and the solvent was removed *in vacuo*. The crude mixture was dry loaded and purified by flash column chromatography (SiO_2, CH_2Cl_2/PE 1:1) to afford **21** as a colorless solid (838 mg, 75%). R_f (CH_2Cl_2/PE 1:1): 0.3; IR (neat): 2961 (m), 1456 (s), 1354 (m), 1267 (w), 1199 (m), 1117 (w), 1010 (m), 976 (m), 899 (s), 826 (s), 698 (w); ^{1}H NMR (400 MHz, $CDCl_3$): 7.67 (d, ^{3}J = 8.4 Hz, 4H), 7.49 (d, ^{3}J = 8.4 Hz, 4H), 5.28 (s, 4H), 1.36 (s, 18H); ^{13}C NMR (101 MHz, $CDCl_3$): 174.0 (C), 155.2 (C), 151.5 (C), 135.6 (CH), 134.3 (C), 129.2 (C), 127.6 (C), 127.5 (CH), 35.2 (C), 31.3 (CH_3), 27.4 (CH_2); ^{77}Se NMR (57 MHz, $CDCl_3$): 729; MS (ESI, $CHCl_3$): 801 (100, $[M+H]^+$); HRMS (ESI, +ve) calcd for $C_{30}H_{30}Br_2N_2S_2Se_2$: 800.8620, found: 800.8618.

Compound 22. To a solution of **21** (800 mg, 1.00 mmol) in THF (25 mL) and DMSO (10 mL) was added NaN_3 (163 mg, 2.50 mmol) and the reaction mixture was stirred for 14 h at rt. The mixture was diluted with CH_2Cl_2 and washed with water, the organic phase was dried over Na_2SO_4 and concentrated under vacuum to afford **22** (725 mg, quant) as a light brown solid. R_f (CH_2Cl_2): 0.4; IR (neat): 2959 (m), 2088 (s), 2926 (m), 1460 (s), 1351 (m), 1261 (w), 1201 (w), 1002 (m), 921 (w), 894 (m), 827 (s), 733 (w); ^{1}H NMR (400 MHz, $CDCl_3$): 7.67 (d, ^{3}J = 8.4 Hz, 4H), 7.49 (d, ^{3}J = 8.4 Hz, 4H), 5.28 (s, 4H), 1.36 (s, 18H); ^{13}C NMR (101 MHz, $CDCl_3$): 174.0 (C), 155.2

(C), 151.5 (C), 135.6 (CH), 134.3 (C), 129.2 (C), 127.6 (C), 127.5 (CH), 35.2 (C), 31.3 (CH_3), 27.4 (CH_2); ^{77}Se NMR (57 MHz, $CDCl_3$): 729; MS (ESI, $CHCl_3$): 727 (100, $[M+H]^+$); HRMS (ESI, +ve) calcd for $C_{30}H_{30}N_8S_2Se_2$: 727.0438, found: 727.0452.

Compound 1. To a solution of **22** (200 mg, 0.28 mmol) in dry DCE (1 mL) in a microwave tube was added DDQ (188 mg, 0.83 mmol) and argon was bubbled through the solution during 1 min. Subsequently the tube was sealed and stirred for 1 h with μW irradiation at 150 °C. After cooling the reaction mixture was diluted with CH_2Cl_2, silica was added and the mixture was concentrated *in vacuo*. The crude mixture was dry loaded and purified by flash column chromatography (CH_2Cl_2) to afford **1** as a poorly soluble beige solid (56 mg, 30%). R_f (CH_2Cl_2): 0.1; IR (neat): 2957 (m), 2905 (w), 2872 (w), 2231 (w), 1437 (s), 1393 (w), 1351 (s), 1260 (w), 1116 (w), 1000 (s), 957 (w), 894 (m), 833 (s), 769 (m), 680 (w); ^{1}H NMR (500 MHz, $CDCl_3$): 7.71 (d, ^3J = 8.5 Hz, 4H), 7.56 (d, ^3J = 8.5 Hz, 4H), 1.39 (s, 18H); ^{13}C NMR (126 MHz, $CDCl_3$): 181.0 (C), 155.9 (C), 152.1 (C), 139.1 (C), 135.5 (CH), 127.8 (CH), 126.8 (C) 114.3 (C), 107.3 (C), 35.1 (C), 31.2 (CH_3); ^{77}Se NMR (57 MHz, $CDCl_3$): 748; MS (ESI, MeOH/$CHCl_3$ 1:1): 667 (100, $[M+H]^+$); HRMS (ESI, +ve) calcd for $C_{30}H_{26}N_4S_2Se_2$: 667.0002, found: 667.0020.

Compound 2. To a suspension of **1** (160 mg, 0.24 mmol) in CH_2Cl_2 (5 mL) mCPBA (162 mg, 0.72 mmol) was added, and the mixture was stirred at rt for 5 h. The mixture was diluted with CH_2Cl_2 and washed with aq. sat. $NaHCO_3$, dried over Na_2SO_4 and concentrated *in vacuo*. The crude product was purified by flash column chromatography (CH_2Cl_2/EtOAc 50:1) to afford **2** as two separate diastereomers, **2 Chiral** (58 mg, 35%) and **2 Meso** (53 mg, 32%). **2 Chiral**: R_f (CH_2Cl_2/EtOAc 20:1): 0.5; IR (neat): 2962 (m), 2876 (w), 1554 (w), 1467 (m), 1395 (m), 1344 (m), 1265 (m), 1079 (s), 1047 (m), 1010 (m), 967 (m), 888 (m), 829 (s), 733 (m), 688 (w); ^{1}H NMR (400 MHz, $CDCl_3$): 7.80 (d, ^3J = 8.6 Hz, 4H), 7.58 (d, ^3J = 8.6 Hz, 4H), 1.31 (s, 18H); ^{13}C

NMR (101 MHz, $CDCl_3$): 194.3 (C), 156.8 (C), 152.1 (C), 142.1 (C), 137.9 (C), 127.4 (CH), 124.4 (CH), 113.4 (C), 111.7 (C), 35.4 (C), 31.2 (CH_2); ^{77}Se NMR (57 MHz, $CDCl_3$): 764; MS (ESI, MeOH/$CHCl_3$ 1:1): 699 (100, $[M+H]^+$); HRMS (ESI, +ve) calcd for $C_{30}H_{26}N_4O_2S_2$Se2: 716.0166, found: 716.0145. **2 Meso**: R_f (CH_2Cl_2/EtOAc 20:1): 0.1; IR (neat): 2962 (m), 2876 (w), 1554 (w), 1467 (m), 1395 (m), 1344 (m), 1265 (m), 1079 (s), 1047 (m), 1010 (m), 967 (m), 888 (m), 829 (s), 733 (m), 688 (w); ^{1}H NMR (400 MHz, $CDCl_3$): 7.77 (d, ^3J = 8.8 Hz, 4H), 7.55 (d, ^3J = 8.8 Hz, 4H), 1.29 (s, 18H); ^{13}C NMR (101 MHz, $CDCl_3$): 194.4 (C), 156.7 (C), 152.1 (C), 142.2 (C), 138.1 (C), 127.3 (CH), 124.4 (CH), 113.4 (C), 111.7 (C), 35.3 (C), 31.2 (CH_2); ^{77}Se NMR (57 MHz, $CDCl_3$): 764; MS (ESI, MeOH/$CHCl_3$ 1:1): 699 (100, $[M+H]^+$).

Compound 3. To a solution of **1** (286 mg, 0.430 mmol) in CH_2Cl_2 (10 mL), mCPBA (289 mg, 1.29 mmol) was added, and the mixture was stirred at rt for 1.5 h before another portion of mCPBA (58 mg, 0.26 mmol) was added. After subsequent stirring for 1 h the mixture was diluted with CH_2Cl_2 and washed with aq. sat. $NaHCO_3$, dried over Na_2SO_4 and concentrated *in vacuo*. The crude product was purified by flash column chromatography (CH_2Cl_2/EtOAc 50:1) to afford **3** (47 mg, 15%) as a faint yellow solid together with **2 Chiral** (57 mg, 19%) and **2 Meso** (65 mg, 22%). R_f (CH_2Cl_2/EtOAc 20:1) 0.6; IR (neat): 2961 (m), 2924 (m), 2854 (w), 1590 (w), 1466 (m), 1398 (w), 1340 (m), 1265 (w), 1159 (s), 1080 (s), 1054 (m), 832 (m), 735 (m), 630 (s); ^{1}H NMR (400 MHz, $CDCl_3$): 8.08 (d, ^3J = 8.6 Hz, 2H), 7.79 (d, ^3J = 8.6 Hz, 2H), 7.64 (d, ^3J = 8.6 Hz, 2H), 7.58 (d, ^3J = 8.6 Hz, 2H), 1.34 (s, 9H), 1.30 (s, 9H); ^{13}C NMR (101 MHz, $CDCl_3$): 195.2 (C), 178.0 (C), 160.2 (C), 156.9 (C), 153.0 (C), 150.4 (C), 143.7 (C), 141.9 (C), 137.7 (C), 133.3 (C), 129.6 (CH), 127.4 (CH), 127.2 (CH), 124.4 (CH), 113.1 (C), 113.0 (C), 113.0 (C), 112.7 (C), 35.7 (C), 35.3 (C), 31.2 (CH_3), 31.1 (CH_3); ^{77}Se NMR (57 MHz, $CDCl_3$): 772, 770; MS (ESI, MeOH/$CHCl_3$ 1:1): 717 (100, $[M+H]^+$); HRMS (ESI, +ve) calcd for $C_{30}H_{26}N_4O_3S_2Se_2$: 732.0115, found: 732.0120

Compound 4. To a solution of **2 Meso** (90 mg, 0.13 mmol) in CH_2Cl_2 (2 mL) mCPBA (287 mg, 1.29 mmol) was added, and the mixture was stirred at rt for 4 h. Subsequently the reaction mixture was directly loaded on to a silica column and purified by flash column chromatography (CH_2Cl_2/EtOAc 30:1) to afford **4** as a faint yellow solid (43 mg, 45%). R_f (CH_2Cl_2/EtOAc 30:1): 0.5; IR (neat): 2966 (w), 1591 (w), 1474 (s), 1402 (w), 1337 (s), 1267 (w), 1158 (s), 1082 (m), 986 (w), 831 (w), 754 (m), 633 (s); ^{1}H NMR (300 MHz, $CDCl_3$): 8.11 (d, ^3J = 8.6 Hz, 4H), 7.66 (d, ^3J = 8.6 Hz, 4H), 1.35 (s, 18H); ^{13}C NMR (101 MHz, $CDCl_3$): 178.8 (C), 160.3 (C), 151.4 (C), 143.4 (C), 133.1 (C), 129.7 (CH), 127.2 (CH), 114.0 (C), 112.8 (C), 35.7 (C), 31.1 (CH_3); ^{77}Se NMR (57 MHz, $CDCl_3$): 776; MS (ESI, MeOH/$CHCl_3$ 1:1): 731 (100, $[M+H]^+$); HRMS (ESI, +ve) calcd for $C_{30}H_{26}N_4O_4S_2Se_2$: 748.0064, found: 748.0090.

Compound 7. To a solution of **20** (1.00 g, 2.12 mmol) in dry THF (20 mL) was added **31** (0.89 g, 5.3 mmol) and argon was bubbled through the solution for 5 min. Subsequently the mixture was cooled to 0 °C and NaH (60% dispersion in mineral oil, 0.21 g, 5.3 mmol) was added in portions and the mixture was stirred for 30 min under argon atmosphere. The reaction mixture was neutralized with sat. NH_4Cl, diluted with CH_2Cl_2 and washed with brine. After drying over Na_2SO_4 and concentration *in vacuo* crude product **28** is obtained and directly used in the next step. To a suspension of crude **28** (2.12 mmol) in DME (50 mL) 1,10-phenanthroline (76 mg, 0.42 mmol), CuI (40 mg, 0.21 mmol) and Cs_2CO_3 (2.07 g, 6.35 mmol) was added and refluxed under an air balloon for 2 h. After cooling to rt the crude reaction mixture was diluted with CH_2Cl_2 and filtered. The filtrate was washed with water, dried over Na_2SO_4 and concentrated *in vacuo*. Purification by flash column chromatography (SiO_2, CH_2Cl_2/PE 1:4) afforded **7** as a colorless solid (0.79 g, 58%). R_f (CH_2Cl_2/PE 1:4): 0.3; IR (neat): 2901 (s), 2846 (m), 1474 (s), 1450 (m), 1344 (m), 1295 (m), 1100 (w), 1037 (w), 976 (m), 905 (m), 884 (s), 822 (w), 671 (m); ^{1}H NMR (400 MHz, $CDCl_3$): 2.76 (s, 6H), 2.29 (m, 12H), 2.12 (br s, 6H), 1.75 (br s, 12H); ^{13}C NMR (101 MHz,

$CDCl_3$): 161.4 (C), 151.7 (C), 131.0 (C), 129.5 (CH), 54.2 (C), 43.1 (CH_2), 36.3 (CH_2), 30.2 (CH), 16.6 (CH_3); ^{77}Se NMR (57 MHz, $CDCl_3$): 737; MS (ESI, MeOH/$CHCl_3$ 1:1): 649 (100, $[M+H]^+$); HRMS (ESI, +ve) calcd for $C_{30}H_{36}N_2S_2$Se2: 649.0723, found: 649.0727.

Compound 10. To a solution of **20** (0.500 g, 1.06 mmol) in dry THF (10 mL) was added **32** (0.46 mL, 2.65 mmol) and argon was bubbled through the solution for 5 min. Subsequently the mixture was cooled to 0 °C and NaH (60% dispersion in mineral oil, 106 mg, 2.65 mmol) was added in portions and the mixture was stirred for 30 min under argon atmosphere. The reaction mixture was neutralized with sat. NH_4Cl, diluted with CH_2Cl_2 and washed with brine. After drying over Na_2SO_4 and concentration *in vacuo* crude product **29** is obtained and directly used in the next step. To a suspension of crude **29** (1.06 mmol) in DME (20 mL) 1,10-phenanthroline (38 mg, 0.21 mmol), CuI (20 mg, 0.11 mmol) and Cs_2CO_3 (1.04 g, 3.18 mmol) was added and refluxed under an air balloon for 2 h. After cooling to rt the crude reaction mixture was diluted with CH_2Cl_2 and filtered. The filtrate was washed with water, dried over Na_2SO_4 and concentrated *in vacuo*. Purification by flash column chromatography (SiO_2, CH_2Cl_2/PE 1:4) afforded **10** as a colorless solid (0.38 g, 60%). R_f (CH_2Cl_2/PE 1:4): 0.3; IR (neat): 2920 (s), 2851 (m), 1466 (s), 1424 (w), 1345 (w), 1310 (w), 982 (s), 913 (s), 884 (m), 723 (m), 683 (w), 672 (w); ^{1}H NMR (400 MHz, $CDCl_3$): 3.32 (t, ^3J = 7.0 Hz, 4H), 2.72 (s, 6H), 1.86 (p, ^3J = 7.3 Hz, 4H), 1.53 – 1.43 (m, 4H), 1.41 – 1.20 (m, 18H), 0.88 (t, ^3J = 6.8 Hz, 6H); ^{13}C NMR (101 MHz, $CDCl_3$): 165.1 (C), 151.2 (C), 129.8 (C), 129.3 (C), 34.8 (CH_2), 31.9 (CH_2), 29.3 (CH_2), 29.2 (CH_2), 29.0 (CH_2), 22.8 (CH_2), 16.5 (CH_3), 14.3 (CH_3); ^{77}Se NMR (57 MHz, $CDCl_3$): 701; MS (ESI, MeOH/$CHCl_3$ 1:1): 605 (100, $[M+H]^+$); HRMS (ESI, +ve) calcd for $C_{26}H_{40}N_2S_2Se_2$: 605.1036, found: 605.1032.

Compound 8. To a solution of **7** (100 mg, 0.155 mmol) in CH_2Cl_2 (10 mL) mCPBA (104 mg, 0.465 mmol) was added in portions, and the mixture was stirred at rt for 30 min. The mixture

was diluted with CH_2Cl_2 and washed with aq. sat. $NaHCO_3$, dried over Na_2SO_4 and concentrated *in vacuo*. The crude product was purified by flash column chromatography (CH_2Cl_2/EtOAc 9:1) to afford **8** as two separate diastereomers, **8 Chiral** (32 mg, 30%) and **8 Meso** (47 mg, 45%). **8 Chiral**: R_f (CH_2Cl_2/EtOAc 9:1): 0.2; IR (neat): 2906 (s), 2850 (m), 1475 (m), 1451 (m), 1342 (w), 1295 (w), 1054 (s), 1025 (s), 887 (m), 737 (m), 703 (w), 682 (w); ^1H NMR (400 MHz, $CDCl_3$): 2.81 (s, 6H), 2.26 – 2.08 (m, 12H), 1.94 – 1.83 (m, 6H), 1.80 – 1.62 (m, 12H); ^{13}C NMR (101 MHz, $CDCl_3$): 180.5 (C), 153.7 (C), 132.7 (C), 131.9 (C), 62.1 (C), 36.1 (CH_2), 35.1 (CH), 29.0 (CH_2), 16.4 (CH_3); ^{77}Se NMR (57 MHz, $CDCl_3$): 765; MS (ESI, MeOH/$CHCl_3$ 1:1): 681 (100, $[M+H]^+$); HRMS (ESI, +ve) calcd for $C_{30}H_{36}N_2O_2S_2Se_2$: 681.0621, found: 681.0604. **8 Meso**: R_f (CH_2Cl_2/EtOAc 9:1): 0.1; IR (neat): 2906 (s), 2850 (m), 1475 (m), 1452 (m), 1342 (w), 1297 (w), 1033 (s), 1025 (s), 886 (m), 683 (w); ^1H NMR (400 MHz, $CDCl_3$): 2.83 (s, 6H), 2.24 – 2.08 (m, 12H), 1.95 – 1.84 (m, 6H), 1.80 – 1.63 (m, 12H); ^{13}C NMR (101 MHz, $CDCl_3$): 180.8 (C), 153.9 (C), 32.8 (C), 132.0 (C), 62.1 (C), 36.2 (CH_2), 35.2 (CH), 29.1 (CH_2), 16.5 (CH_3); ^{77}Se NMR (57 MHz, $CDCl_3$): 766; MS (ESI, MeOH/$CHCl_3$ 1:1): 681 (100, $[M+H]^+$).

Compound 9. To a solution of **7** (100 mg, 0.155 mmol) in CH_2Cl_2 (5 mL) mCPBA (347 mg, 1.55 mmol) was added in portions, and the mixture was stirred at rt for 2 h. The mixture was diluted with CH_2Cl_2 and washed with aq. sat. $NaHCO_3$, dried over Na_2SO_4 and concentrated *in vacuo*. The crude product was purified by flash column chromatography (CH_2Cl_2/EtOAc 100:1) to afford **9** as a colorless solid (50 mg, 46%). R_f (CH_2Cl_2/EtOAc 100:1): 0.2; IR (neat): 2910 (m), 2853 (w), 1476 (w), 1453 (w), 1304 (s), 1139 (s), 1103 (w), 1045 (m), 1006 (w), 890 (m), 727 (m), 697 (m), 646 (w); ^1H NMR (400 MHz, $CDCl_3$): 2.87 (s, 6H), 2.20 (br s, 18H), 1.80 – 1.61 (m, 12H); ^{13}C NMR (101 MHz, $CDCl_3$): 167.1 (C), 153.9 (C), 134.8 (C), 134.2 (C), 63.5 (C), 35.7 (CH_2), 35.0 (CH), 28.3 (CH_2), 16.6 (CH_3); ^{77}Se NMR (57 MHz, $CDCl_3$): 776; MS (ESI,

MeOH/$CHCl_3$ 1:1): 713 (100, $[M+H]^+$); HRMS (ESI, +ve) calcd for $C_{30}H_{36}N_2O_4S_2Se_2$: 713.0520, found: 713.0538.

Compound 11. To a solution of **10** (50 mg, 83 µmol) in CH_2Cl_2 (2 mL) mCPBA (56 mg, 25 µmol) was added in portions, and the mixture was stirred at rt for 2 h. The mixture was diluted with CH_2Cl_2 and washed with aq. sat. $NaHCO_3$, dried over Na_2SO_4 and concentrated *in vacuo*. The crude product was purified by flash column chromatography (CH_2Cl_2/EtOAc 9:1) to afford **11** as two, not completely separable, diastereomers, **11 Chiral** (21 mg, 38%) and **11 Meso** (19 mg, 34%). **11 Chiral**: R_f (CH_2Cl_2/EtOAc 9:1): 0.3; IR (neat): 2955 (w), 2922 (m), 2853 (m), 1489 (m), 1470 (m), 1312 (m), 1047 (s), 993 (m), 888 (w), 723 (w), 680 (w); ^{1}H NMR (400 MHz, $CDCl_3$): 3.28 – 3.06 (m, 4H), 2.78 (s, 6H), 2.03 – 1.89 (m, 2H), 1.83 – 1.70 (m, 2H), 1.53 – 1.38 (m, 4H), 1.38 – 1.24 (m, 16H), 0.85 (t, ^3J = 7.1 Hz, 6H); ^{13}C NMR (101 MHz, $CDCl_3$): 183.0 (C), 153.7 (C), 132.9 (C), 132.3 (C), 57.3 (CH_2), 31.8 (CH_2), 29.2 (CH_2), 29.1 (CH_2), 28.8 (CH_2), 22.7 (CH_2), 21.7 (CH_2), 16.4 (CH_3), 14.3 (CH_3); ^{77}Se NMR (57 MHz, $CDCl_3$): 734; MS (ESI, MeOH/$CHCl_3$ 1:1): 637 (100, $[M+H]^+$); HRMS (ESI, +ve) calcd for $C_{26}H_{40}N_2O_2S_2Se_2$: 637.0934, found: 637.0926. **11 Meso**: R_f (CH_2Cl_2/EtOAc 9:1): 0.25; IR (neat): 2957 (w), 2922 (m), 2853 (w), 1486 (m), 1469 (w), 1313 (m), 1047 (s), 994 (m), 889 (w), 724 (w); ^{1}H NMR (400 MHz, $CDCl_3$): 3.30 – 3.06 (m, 4H), 2.80 (s, 6H), 2.04 – 1.92 (m, 2H), 1.84 – 1.71 (m, 2H), 1.57 – 1.41 (m, 4H), 1.37 – 1.26 (m, 16H), 0.87 (t, ^3J = 6.9 Hz, 6H); ^{13}C NMR (101 MHz, $CDCl_3$): 182.9 (C), 153.7 (C), 132.9 (C), 132.3 (C), 57.2 (CH_2), 31.8 (CH_2), 29.2 (CH_2), 29.1 (CH_2), 28.8 (CH_2), 22.7 (CH_2), 21.6 (CH_2), 16.4 (CH_3), 14.3 (CH_3); ^{77}Se NMR (57 MHz, $CDCl_3$): 735; MS (ESI, MeOH/$CHCl_3$ 1:1): 637 (100, $[M+H]^+$).

Compound 12. To a solution of **10** (50 mg, 83 µmol) in CH_2Cl_2 (2 mL) mCPBA (347 mg, 830 µmol) was added in portions, and the mixture was stirred at rt for 1 h. The mixture was diluted with CH_2Cl_2 and washed with aq. sat. $NaHCO_3$, dried over Na_2SO_4 and concentrated *in vacuo*. The

crude product was purified by flash column chromatography (CH_2Cl_2) to afford **12** as a colorless solid (14 mg, 25%). R_f (CH_2Cl_2): 0.5; IR (neat): 2923 (m), 2854 (w), 1483 (w), 1464 (w), 1318 (s), 1235 (s), 1011 (m), 889 (w), 760 (w), 724 (w), 615 (s), 556 (m), 546 (m); ^{1}H NMR (400 MHz, $CDCl_3$): 3.62 – 3.44 (m, 4H), 2.86 (s, 6H), 2.02 – 1.79 (m, 4H), 1.53 – 1.38 (m, 4H), 1.37 – 1.19 (m, 16H), 0.85 (t, ^3J = 6.9 Hz, 6H); ^{13}C NMR (101 MHz, $CDCl_3$): 169.4 (C), 153.6 (C), 134.5 (C), 134.4 (C), 54.3 (CH_2), 31.8 (CH_2), 29.0 (CH_2), 28.0 (CH_2), 28.5 (CH_2), 22.7 (CH_2), 22.5 (CH_3), 16.6 (CH_2), 14.2 (CH_3); ^{77}Se NMR (57 MHz, $CDCl_3$): 738; MS (ESI, MeOH/$CHCl_3$ 1:1): 669 (100, $[M+H]^+$); HRMS (ESI, +ve) calcd for $C_{26}H_{40}N_2O_4S_2Se_2$: 669.0833, found: 669.0860.

Compound 6. To a solution of **5** (100 mg, 156 mmol) in CH_2Cl_2 (2 mL) mCPBA (105 mg, 467 µmol) was added in portions, and the mixture was stirred at rt for 1 h. The mixture was diluted with CH_2Cl_2 and washed with aq. sat. $NaHCO_3$, dried over Na_2SO_4 and concentrated *in vacuo*. The crude product was purified by flash column chromatography (CH_2Cl_2/EtOAc 30:1) to afford **6** as two separate diastereomers, **6 Chiral** (21 mg, 20%) and **6 Meso** (13 mg, 12%). **6 Chiral**: R_f (CH_2Cl_2/EtOAc 30:1): 0.3; IR (neat): 2959 (m), 2870 (w), 1476 (m), 1461 (w), 1392 (m), 1316 (w), 1267 (w), 1081 (s), 1055 (s), 1008 (m), 884 (m), 828 (s), 732 (m), 678 (w); ^{1}H NMR (400 MHz, $CDCl_3$): 7.78 (d, ^3J = 8.6 Hz, 4H), 7.54 (d, ^3J = 8.6 Hz, 4H), 2.71 (s, 6H), 1.30 (s, 18H); ^{13}C NMR (101 MHz, $CDCl_3$): 183.7 (C), 155.8 (C), 153.5 (C), 139.8 (C), 132.6 (C), 132.4 (C), 126.9 (CH), 124.4 (CH), 35.2 (C), 31.3 (CH_3), 16.4 (CH_3); ^{77}Se NMR (57 MHz, $CDCl_3$): 707; MS (ESI, MeOH/$CHCl_3$ 1:1): 677 (100, $[M+H]^+$); HRMS (ESI, +ve) calcd for $C_{30}H_{32}N_2O_2S_2Se_2$: 677.0308, found: 677.0304. **6 Meso**: R_f (CH_2Cl_2/EtOAc 30:1): 0.25; IR (neat): 2960 (m), 2924 (w), 2853 (w), 1488 (m), 1461 (w), 1391 (m), 1311 (m), 1264 (w), 1077 (s), 1051 (s), 1008 (m), 884 (m), 828 (s), 732 (m), 690 (w); ^{1}H NMR (400 MHz, $CDCl_3$): 7.76 (d, ^3J = 8.6 Hz, 4H), 7.50 (d, ^3J = 8.6 Hz, 4H), 2.70 (s, 6H), 1.28 (s, 18H); ^{13}C NMR (101 MHz, $CDCl_3$): 183.7 (C), 155.7 (C), 153.5 (C),

139.9 (C), 132.6 (C), 132.4 (C), 126.8 (CH), 124.4 (CH), 35.2 (C), 31.2 (CH_3), 16.4 (CH_3); ^{77}Se NMR (57 MHz, $CDCl_3$): 707; MS (ESI, MeOH/$CHCl_3$ 1:1): 677 (100, $[M+H]^+$).

3. Catalyst evaluation

3.1. Rate studies

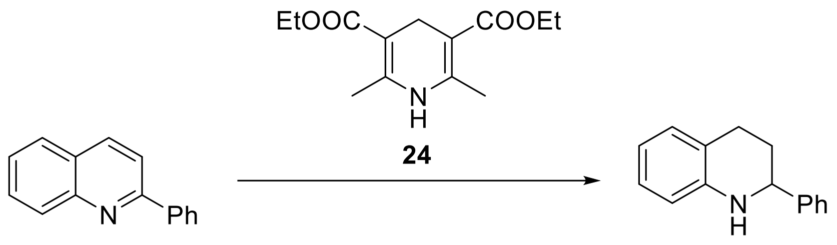

Scheme S5. Reactions with substrates **23** (128 mM) and **24** (281 mM) together with 1-30 mol% catalysts **1-12** and mesitylene as internal standard were conducted in dry CD_2Cl_2 at 20 ºC under argon atmosphere.

As previously described in reference S1, catalyst and substrates were weighted in to a screw cap vial and suspended in excess dry CD_2Cl_2. The mixture was degassed by bubbling of argon, until the desired final solvent volume was reached; comprising substrate **23** (128 mM), Hantzsch ester **24** (281 mM), desired catalyst (i.e. 30 mol%, 38 mM) and internal standard (43 mM). The vial was tightly sealed and stirred at 20 °C. ^{1}H NMR spectra of aliquots of the reaction mixture (5-10 μL) diluted in $CDCl_3$ (0.5 mL) were recorded at varying time intervals. Integrals associated with the protons in quinoline **23** at positions 4 and 5 (δ 7.89 ppm; d, 1H and δ 7.84 ppm; d, 1H) and of the hydroquinoline product **25** at position 8 (δ 6.59 ppm; d, 1H) were observed as depicted in Fig. S1. The concentration of the products was determined by comparing the integration of the

above mentioned resonances with those of the internal standard mesitylene (δ 6.80 ppm; s, 3H) in the crude proton NMR.

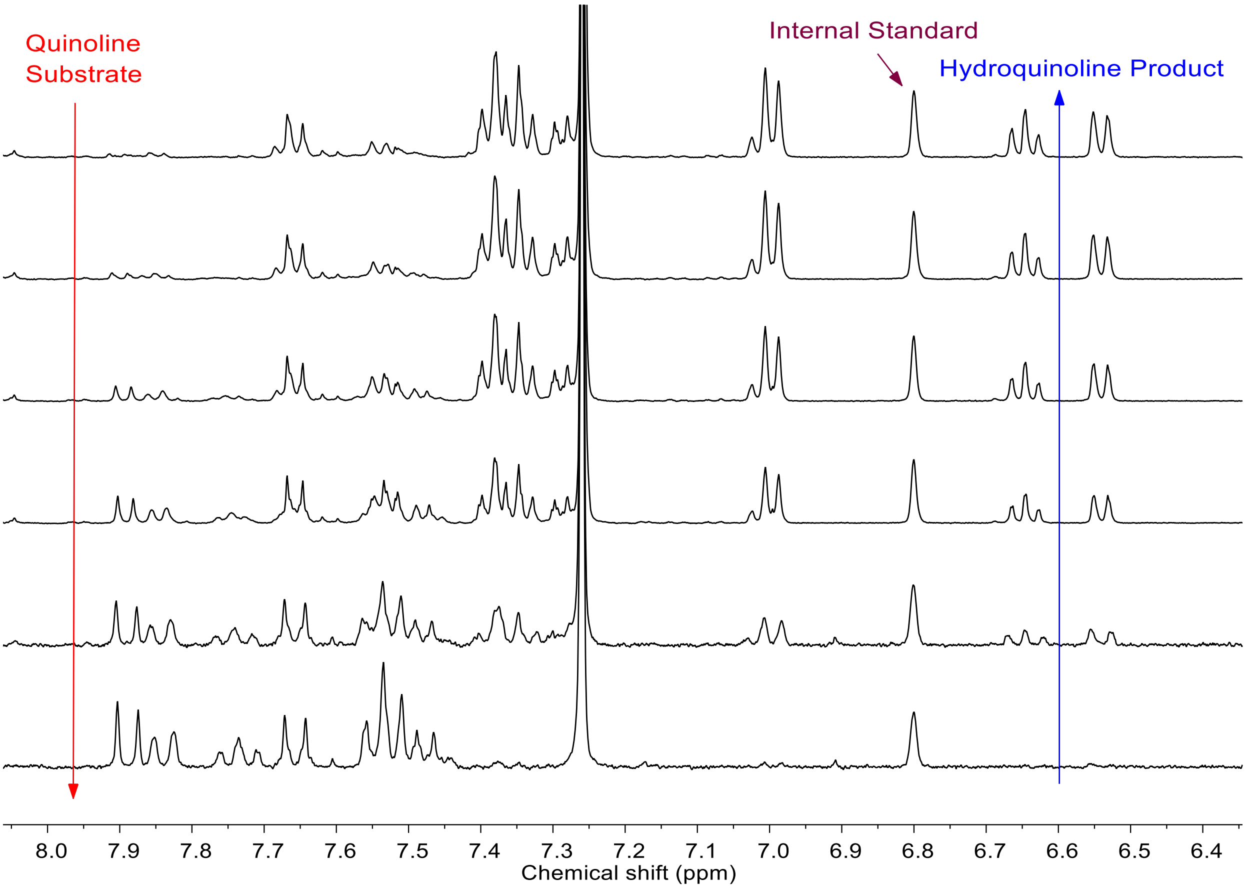

Fig. 1 Representative ^{1}H NMR showing the reaction evolution of a mixture of quinoline substrate **23** (128 mM), Hantzsch ester **24** (281 mM), internal standard (43 mM) and catalyst **4** (38 mM) in CD_2Cl_2 at 20 ºC. Samples (10 µL) were taken in appropriate intervals and diluted in $CDCl_3$ (0.5 mL). Relevant substrate signals for integration are shown with a red arrow, product signals with a blue arrow. Signals were normalized versus the internal standard, indicated in purple.

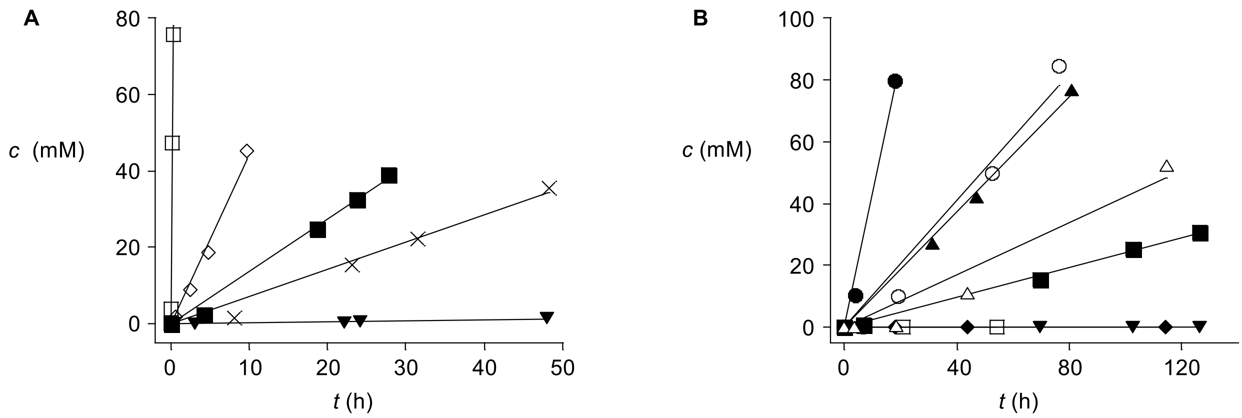

Fig. S2 (A) Formation of product **25** with time for bis cyano BDS catalysts **1** (□), **2 Chiral** (■), **3** (○) and **4** (●); (B) and for bis methyl BDS catalysts **5** (□), **6 Chiral** (▲), **7** (♦), **8 Chiral** (●), **9** (△), **10** (▼), **11 Chiral / 11 Meso** (■) and **12** (○).

The concentration of product **25** was plotted against reaction time, and the initial velocities were determined from linear fitting (Fig. S2). Apparent second-order rate constants were determined from Equation (S1).

$$k_{\mathrm{app}} = v_{\mathrm{ini}} \,/\, ([\mathbf{23}]_0\ [\mathbf{24}]_0) \qquad \text{(S1)}$$

ΔE_{a} was approximated by Equation (S2).

$$k_{\mathrm{cat}}/k_{\mathrm{uncat}} = \exp\,(-\Delta E_{\mathrm{a}} \,/\, RT) \qquad \text{(S2)}$$

Where k_{cat} corresponds to the second-order rate constant of a reaction with catalysts **1-12** and k_{uncat} to the second-order rate constant of the uncatalyzed reaction.

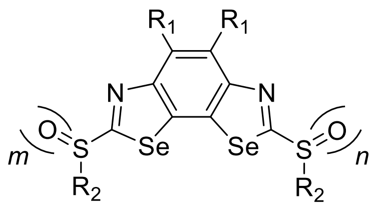

Fig. S3 General structure for BDS Catalysts.

Table S1 Characteristics of chalcogen-bonding catalysts

Cat[a]	R^1	n	m	R^2	η (%)[b]	k_{cat}/k_{uncat}[c]	ΔE_a (kJ mol^{-1})[d]	E_{int} (kcal mol^{-1})[e]
**4**	CN	2	2	PTBP	93	15000	–29.1	–53.0 (–41.6)
**4**	CN	2	2	PTBP	88[f]	n.d.	n.d.	–53.0 (–41.6)
**4**	CN	2	2	PTBP	93[g]	n.d.	n.d.	–53.0 (–41.6)
**3**	CN	1	2	PTBP	88	3200	–19.6	–49.2 (–38.0)
**2**	CN	1	1	PTBP	78	970[h]	–16.8	–45.2 (–34.3)[i]
**2**	CN	1	1	PTBP	79	500[j]	–15.1	n.d.
**1**	CN	0	0	PTBP	n.d.	100	–11.3	–37.2 (–32.4)[i]
**6**	CH_3	1	1	PTBP	93	660[h]	–15.8	–34.1
**5**	CH_3	0	0	PTBP	n.d.	≥10	n.d.	–25.7
**9**	CH_3	2	2	Ad	47	300	–13.9	n.d.
**8**	CH_3	1	1	Ad	97	3100[h]	–19.6	n.d.
**7**	CH_3	0	0	Ad	n.d.	≥10	n.d.	n.d.
**12**	CH_3	2	2	Oct	90	730	–16.1	n.d.
**11**	CH_3	1	1	Oct	68	170[k]	–12.5	n.d.
**10**	CH_3	0	0	Oct	n.d.	≥10	n.d.	n.d.
**13**[l]	-	-	-	-	96	490	–17.5	–36.1

[a]For structures see Scheme S3, S4 and Fig. 3 in the main text. [b]Yield of the reduced product, determined by ^{1}H NMR integration. [c]Rate enhancement for product formation from **23** (128 mM) and **24** (281 mM) in CD_2Cl_2 at 20 ºC with 30 mol% catalyst, compared to k_{uncat} = 3.9 x 10^{-5} M^{-1} h^{-1} (taken from reference S1), monitored by ^{1}H NMR spectroscopy. [d]Transition state stabilization calculated from Equation S2. [e]Calculated interaction energies for a 1:1 chloride complex, on the M062X/6-311G** level. [f]Yield with 5 mol% catalyst loading determined after 12 h. [g]Yield with 1 mol% [h] Data obtained with chiral diastereomer. [i]*syn* (*anti*) conformer. [j]Data obtained with meso compound. [k]Data obtained with a mixture of diastereomers. [l]Data from reference S1. n.d.: not determined.

3.2. Substrate scope

A

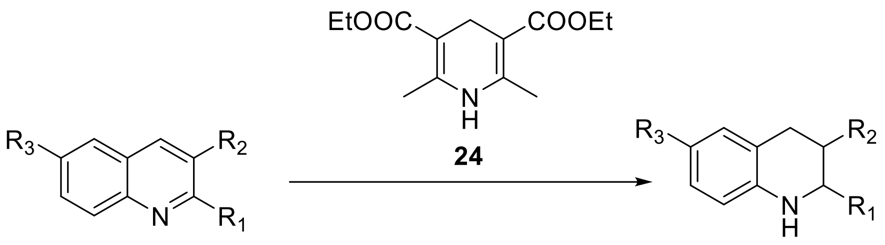

23a-h **25a-h**

23a: R_1 = Ph, R_2 = R_3 = H
23b: R_1 = R_2 = R_3 = H
23c: R_1 = Cl, R_2 = R_3 = H
23d: R_1 = R_2 = H, R_3 = NO_2
23e: R_1 = Me, R_2 = H, R_3 = F
23f: R_1 = R_3 = H, R_2 = Br
23g: R_1 = R_2 = H, R_3 = OMe
23h: R_1 = R_2 = H, R_3 = Me

25a: R_1 = Ph, R_2 = R_3 = H
25b: R_1 = R_2 = R_3 = H
25c: R_1 = Cl, R_2 = R_3 = H
25d: R_1 = R_2 = H, R_3 = NO_2
25e: R_1 = Me, R_2 = H, R_3 = F
25f: R_1 = R_3 = H, R_2 = Br
25g: R_1 = R_2 = H, R_3 = OMe
25h: R_1 = R_2 = H, R_3 = Me

B

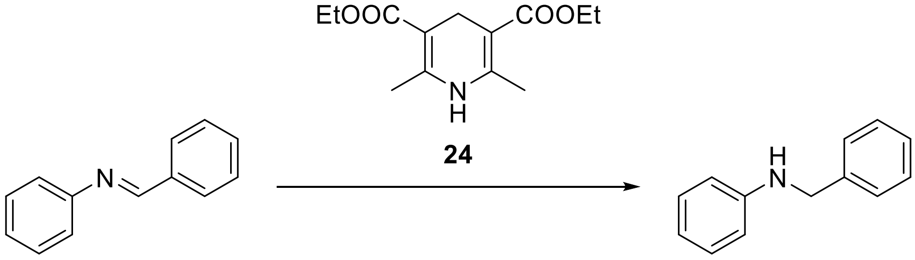

33 **34**

Scheme S6. (A) Reactions with quinoline substrates **23a-h** (128 mM) and Hantzsch ester **24** (281 mM) together with 1 mol% catalysts **4** and mesitylene as internal standard were conducted in dry CD_2Cl_2 at 20 ºC under argon atmosphere. (B) Reaction with *N*-benzylidene-aniline **33** (250 mM) and Hantzsch ester **10** (375 mM) together with 1 mol% catalyst **4** and mesytilene as internal standard were conducted in dry CD_2Cl_2 at 20 ºC under argon atmosphere.

As previously described in section 3.1, catalyst and substrates were weighted in to a screw cap vial and suspended in excess dry CD_2Cl_2. The mixture was degassed by bubbling of argon,

until the desired final solvent volume was reached; comprising quinoline substrates **23a-h** (128 mM), Hantzsch ester **24** (281 mM), 1 mol% catalyst **4** and internal standard (43 mM). The vial was tightly sealed and stirred at 20 °C. ^{1}H NMR spectra of aliquots of the reaction mixture (5-10 μL) diluted in $CDCl_3$ (0.5 mL) were recorded after 24 h and 48 h. The concentration of the products was determined by comparing the integration of product resonances with those of the internal standard mesitylene in the crude proton NMR.

Table S2 Substrate scope

S^{a}	R_1	R_2	R_3	*t* (h)^{b}	*η* (%)^{c}
**23a**	Ph	H	H	24	93
**23b**	H	H	H	24	89 (98)^{d}
**23c**	Cl	H	H	48	0
**23d**	H	H	NO_2	48	9
**23e**	Me	H	F	24	80 (88)^{d}
**23f**	H	Br	H	24	98
**23g**	H	H	OMe	24	32 (58)^{d}
**23h**	H	H	Me	24	97
**33**	-	-	-	24	99

^{a}For structures see Scheme S6. ^{b}Reaction time. ^{c}Yield of the reduced product, determined by ^{1}H NMR integration against internal standard. ^{d}Yields in brackets determined after 48 h reaction time.

4. Anion binding

As in reference S1, absorption spectra were recorded of the catalysts **1**, **2 Chiral**, **3** and **4** in THF in the presence of TBACl (0 – 20 mM). Differences in absorbances at λ_{max}, ΔI were plotted versus acceptor concentration and curve-fitted to a 1:1 binding isotherm in order to determine dissociation constants (K_D’s) according to Equation (S3), as reported in reference S4:

$$\Delta I = (\Delta I_{max} / [T]_0) \times (0.5 \times [A] + 0.5 \times ([T]_0 + K_D) - (0.5 \times (([A]^2) + (2 \times ([A]) \times (K_D - [T]_0)) + (K_D + [T]_0)^2)^{0.5})) \qquad (S3)$$

where $\Delta I = |I - I_0|$, [A] = concentration of TBACl, and $[T]_0$ = concentration of catalysts **1**, **2 Chiral**, **3** and **4**.

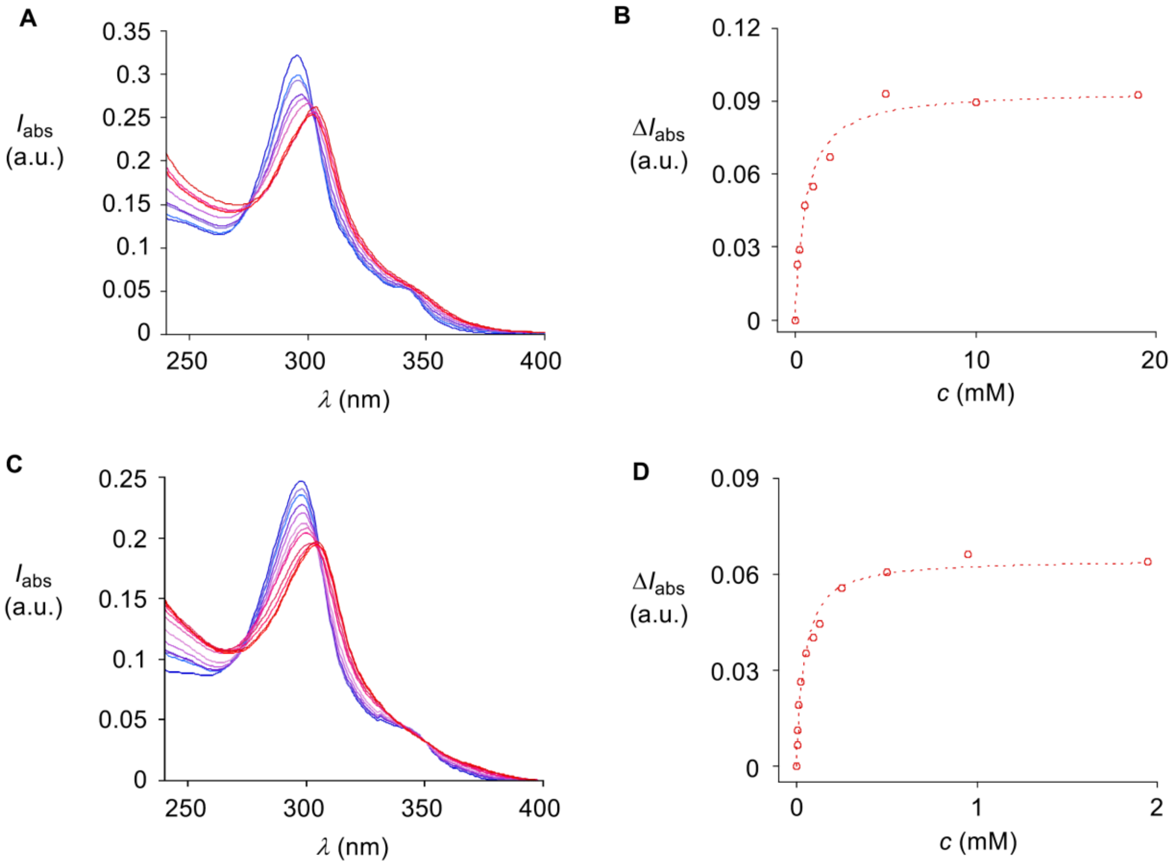

Fig. S4 Changes in absorption with increasing concentration of TBACl (blue to red) for catalysts **2 Chiral** (A) and catalyst **3** (C). Nonlinear fitting of changes in absorbance taken at λ_{max} versus TBACl concentration to Equation (S3) for catalyst **2 Chiral** (B) and for catalyst **3** (D).

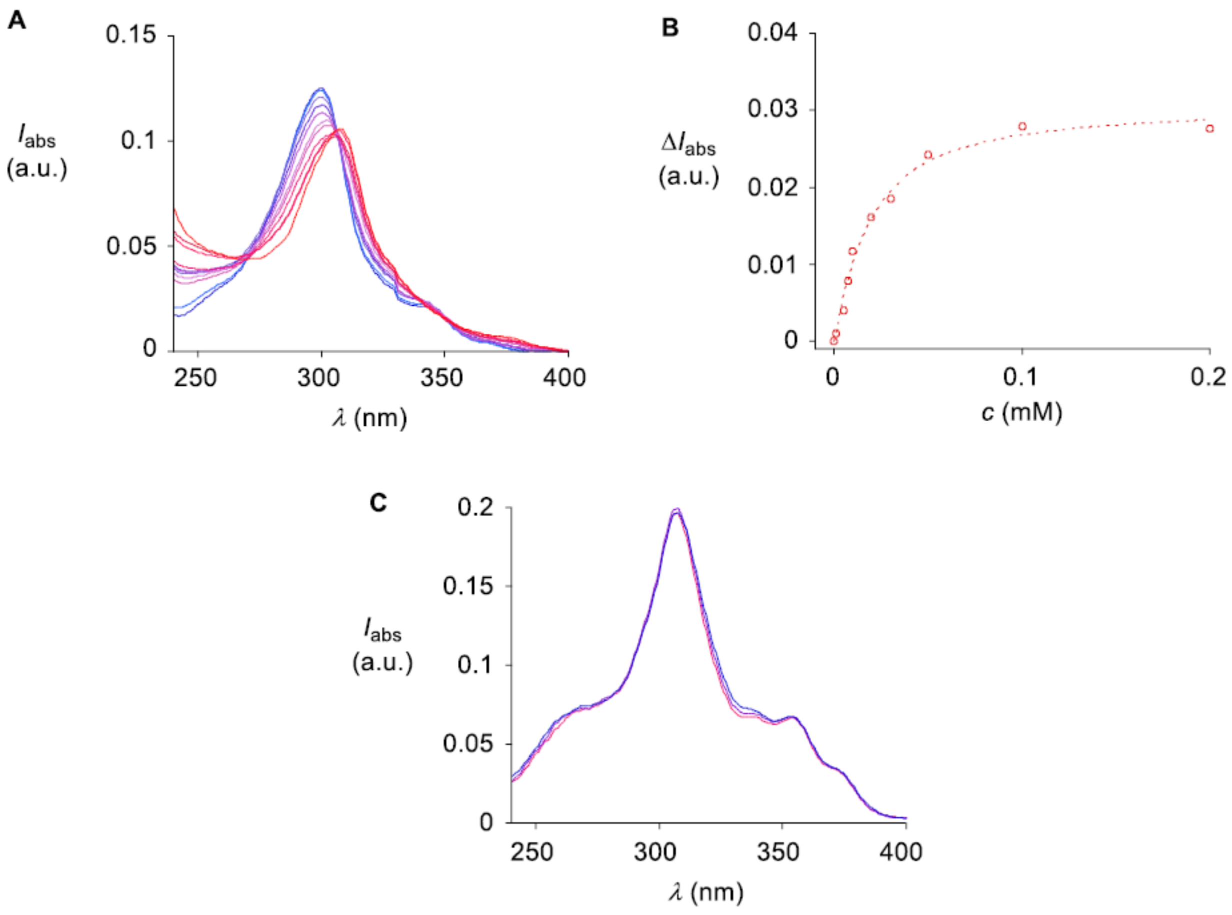

Fig. S5 Changes in absorption with increasing concentration of TBACl (cyan to red) for catalysts **4** (A) and catalyst **1** (C). (B) Nonlinear fitting of changes in absorption taken at λ_{max} versus TBACl concentration to equation (S3) for catalyst **4**.

5. Electrochemistry

The reduction potentials of catalysts **1**, **2 Chiral, 3**, and **4** were determined using cyclic voltammetry (CV, scan rate 100 mV/s) vs Fc^+/ Fc in CH_2Cl_2 (supporting electrolyte: 100 mM $TBAPF_6$, working electrode: Glassy carbon, counter electrode: Pt wire, reference electrode: SCE). No reduction was visible for **5** within the solvent limit of CH_2Cl_2. LUMO energies vs vacuum were calculated from first reduction peaks in CV using Equation S4.

$$E_{\mathrm{LUMO}} = -\ 5.1\ \mathrm{eV} - E_{\mathrm{CV}}\ \mathrm{vs}\ (\mathrm{Fc}^+/\ \mathrm{Fc}) \qquad \text{(S4)}$$

where E_{CV} is the average potential of the first redox couple, relative to the internal standard ferrocene / ferrocenium.[S5]

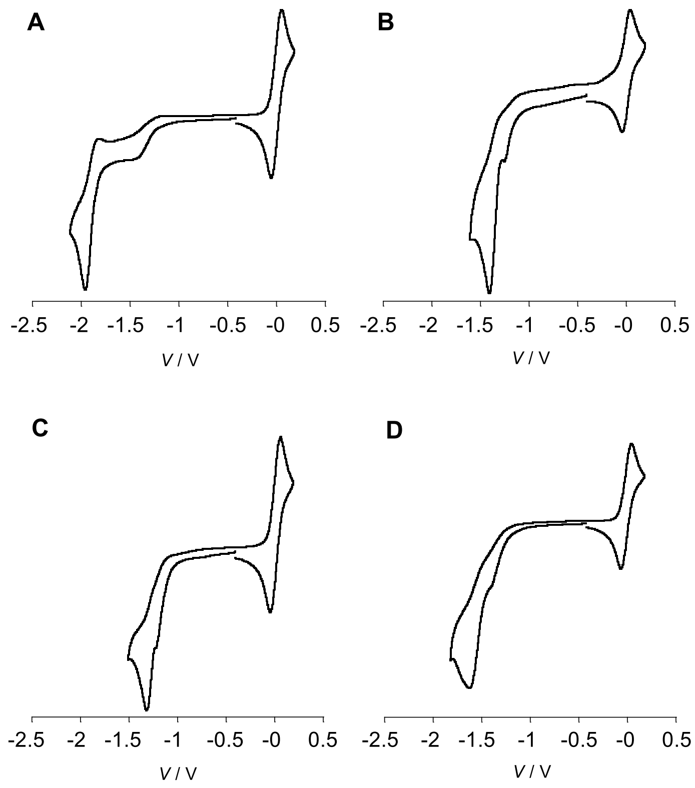

Fig. S6 The cyclic voltammograms with internal Fc^+/Fc standard, for catalysts (A) **1**, (B) **2 Chiral**, (C) **3** and (D) **12**.

Table S3 Characteristics of chalcogen-bonding catalysts

Cat[a]	R^1	n	m	R^2	K_D (μM)[b]	E_{LUMO} (eV)[c]	λ_{max} (nm)[d]	ε ($mM^{-1}cm^{-1}$)[e]
**4**	CN	2	2	PTBP	11 ± 2	–3.81	300	60.3
**3**	CN	1	2	PTBP	37 ± 6	–3.74	298	69.5
**2**	CN	1	1	PTBP	530 ± 90[i]	–3.54[i]	295[i]	62.5[i]
**1**	CN	0	0	PTBP	n.d.[g]	–3.21	307	79.1
**5**	CH_3	0	0	PTBP	n.d.	n.d.[h]	332	27.2.
**13**[f]	-	-	-	-	1130 ± 30	–3.70	376	n.d.

[a]For structures see Scheme S4 and Fig. 3 in the main text. [b]Dissociation constant measured by absorption spectroscopy with TBACl in THF (Fig. S4 and S5). [c]Energy of the lowest unoccupied molecular orbital (LUMO) in eV against vacuum, with -5.1 eV for Fc^+/Fc as reference, determined by CV. [d]Absorption maxima in THF. [e]Extinction coefficient at λ_{max}. [f]Data from reference S1. [g]Only minimal changes in the UV absorption spectrum were observed upon the addition of TBACl. [h]No reduction was observable within the solvent limit of CH_2Cl_2. [i]Data obtained with a chiral diastereomer. n.d.: not determined.

6. Computational studies

As in ref. S6: Calculations were performed using the Gaussian09.D01 program,[S7] geometry optimizations the M062X functional[S8] and 6-311G** basis set. Minima for chloride complexes were confirmed by vibrational analysis. Representations of optimized geometries were obtained with GausView program.[S9] Hi-res structures were produced with the drawing program Mercury CSD 2.0.[S10]

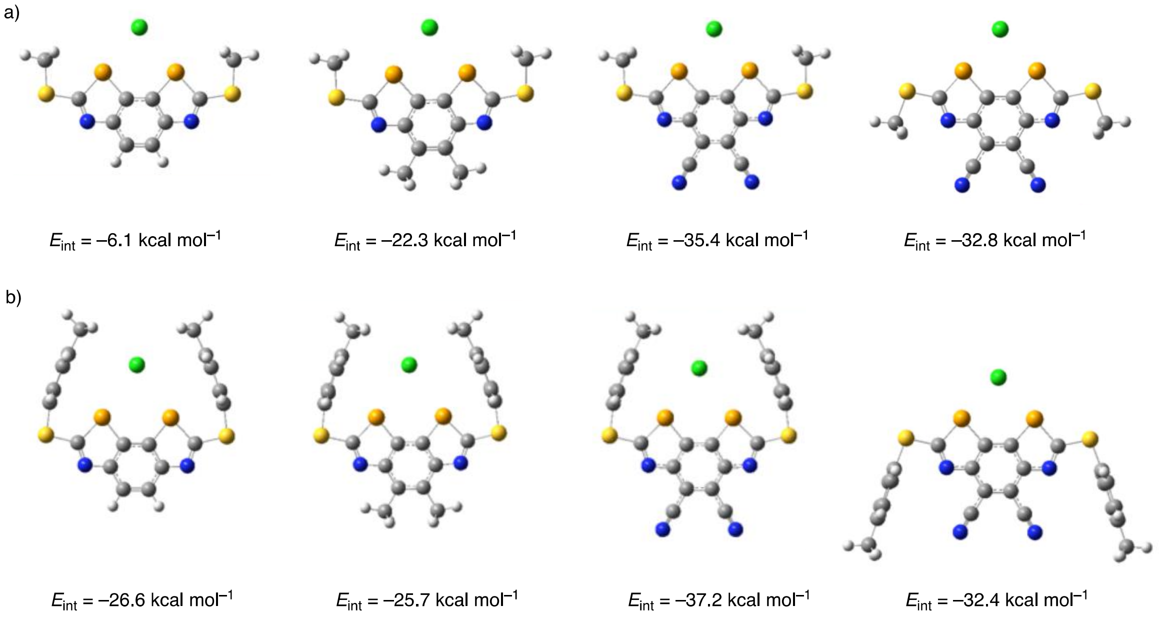

Fig. S7 DFT-M062X/6-311G** models of chloride (green) bound to BDS with (a) R^1 = H, R^2 = Me, *syn* conformer (left), R^1 = Me, R^2 = Me, *syn* conformer (middle left), R^1 = CN, R^2 = Me, *syn* conformer (middle right), R^1 = CN, R^2 = Me, *anti* conformer (right), and (b) R^1 = H, R^2 = pMePh, *syn* conformer (left), R^1 = Me, R^2 = pMePh, *syn* conformer (middle left), R^1 = CN, R^2 = pMePh, *syn* conformer **1'** (middle right), R^1 = CN, R^2 = pMePh, *anti* conformer **1'** (right).

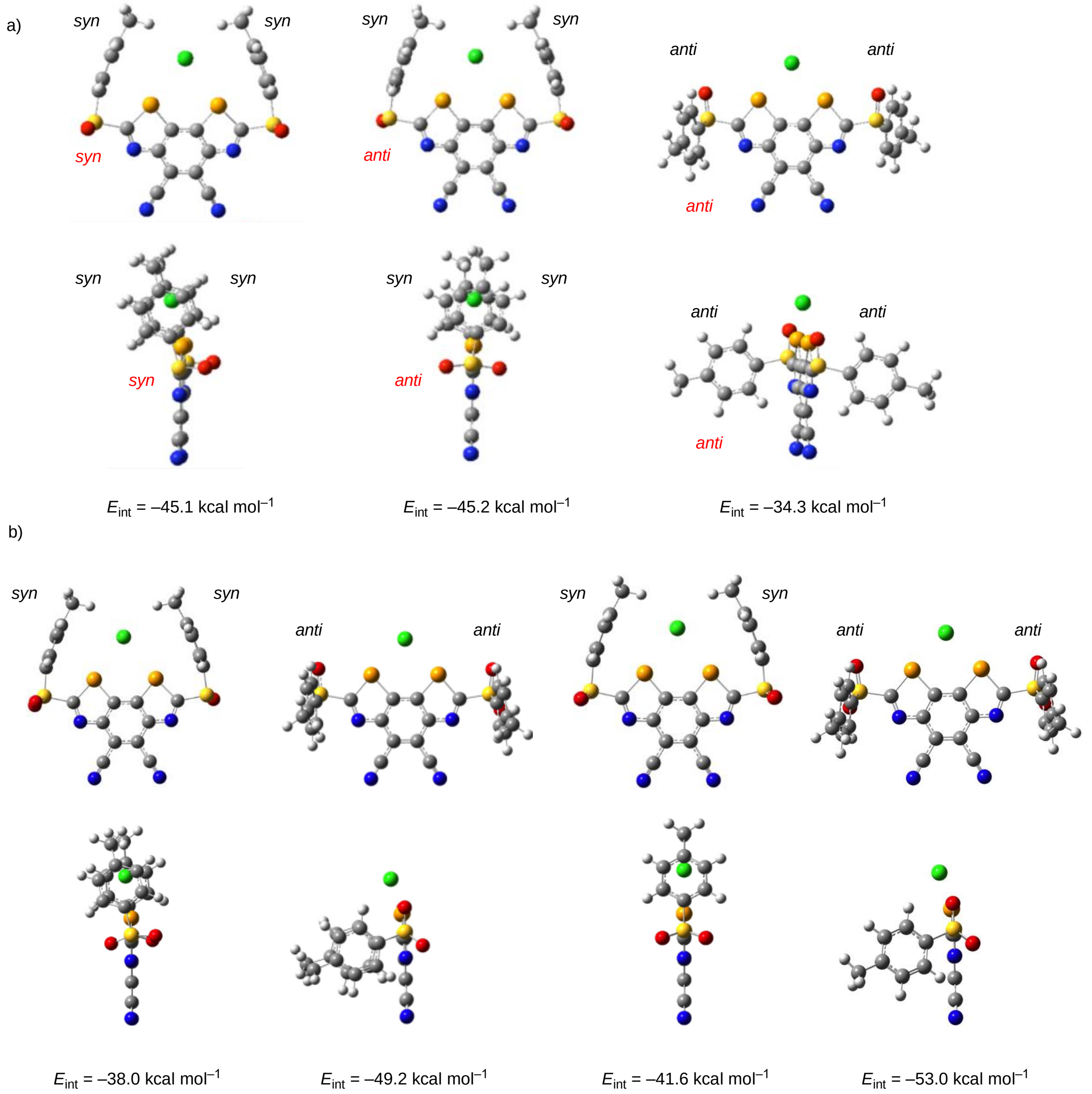

Fig. S8 Front (top) and side view (bottom) of DFT-M062X/6-311G** models of chloride (green) bound to a) BDS **2'** (R^2 = pMePh) as *syn* conformer with *syn* sulfoxides (**2' Meso**, left), as *syn* conformer with *anti* sulfoxides (**2' Chiral**, middle) and as *anti* conformer with *anti* sulfoxides (**2' Chiral**, right), and to b) BDS **3'** (R^2 = pMePh) as *syn* (left) and *anti* conformer (middle left) and BDS **4'** (R^2 = pMePh) as *syn* (middle right) and *anti* conformer (right).

7. Crystallographic data

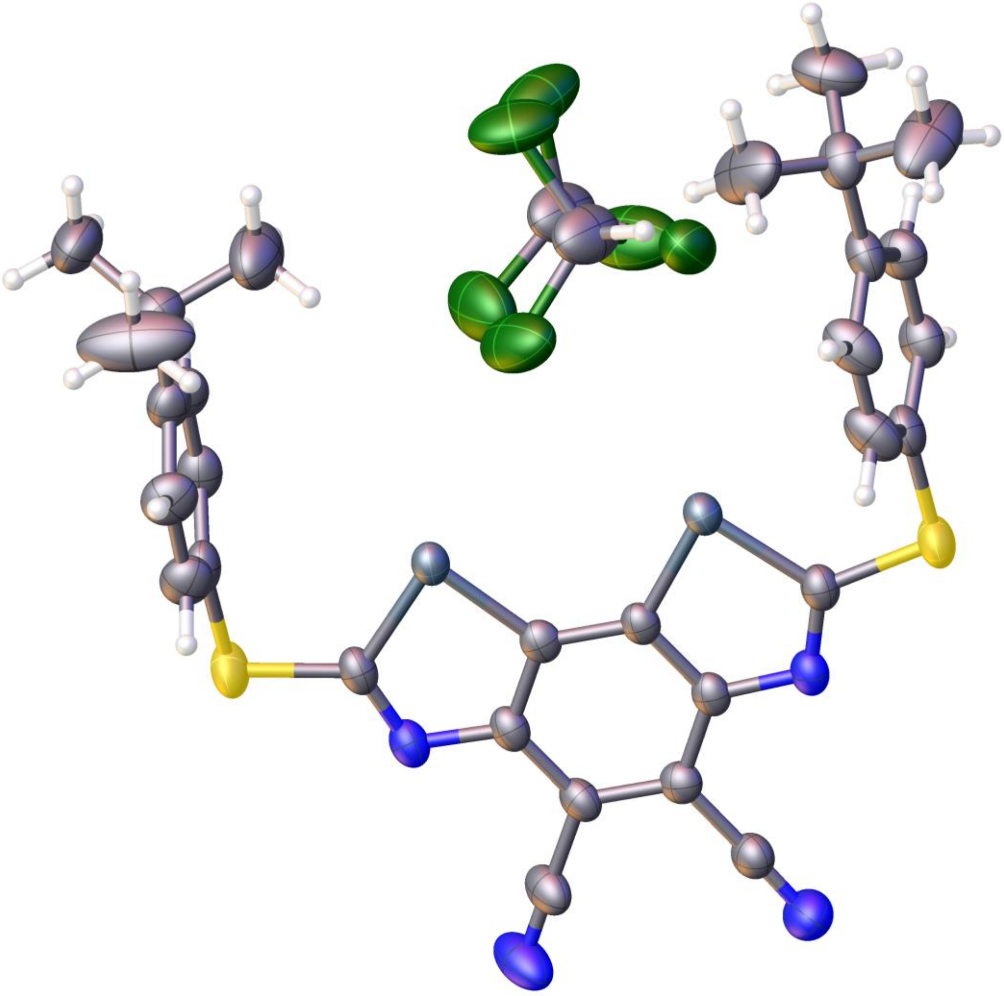

Fig. S9 Crystal structure of catalyst **1** with an encapsulated solvent molecule ($CHCl_3$), depicted with displacement ellipsoids drawn at 50 percent probability. CCDC 1568432 contains the supplementary crystallographic data which can be obtained free of charge from the Cambridge Crystallographic Data Centre via: www.ccdc.cam.ac.uk/data_request/cif.

Table S3 Crystallographic data[a]

Parameter	Data
Empirical formula	$C_{31}H_{27}Cl_3N_4S_2Se_2$
Formula weight	783.95
Temperature/K	180
Crystal system	monoclinic
Space group	$P2_1/c$
a/Å	17.9134(2)
b/Å	11.53190(10)
c/Å	18.2586(2)
α/°	90
β/°	117.2300(10)
γ/°	90
Volume/Å^3	3353.78(7)
Z	4
ρ_{calc}g/cm^3	1.553
μ/mm^{-1}	6.34
F(000)	1568
Crystal size/mm^3	0.205 × 0.127 × 0.077
Radiation	CuKα (λ = 1.54184)
2Θ range for data collection/°	9.406 to 148.58
Index ranges	-22 ≤ h ≤ 22, -14 ≤ k ≤ 13, -22 ≤ l ≤ 22
Reflections collected	55832
Independent reflections	6780 [R_{int} = 0.0313, R_{sigma} = 0.0141]
Data/restraints/parameters	6780/36/404
Goodness-of-fit on F^2	1.039
Final R indexes [I>=2σ (I)]	R_1 = 0.0395, wR_2 = 0.1109
Final R indexes [all data]	R_1 = 0.0423, wR_2 = 0.1146
Largest diff. peak/hole / e Å^{-3}	1.03/-1.17

[a]All parameters for crystal structure determination and resolving.

8. Supplementary references

S1 S. Benz, J. Lopez-Andarias, J. Mareda, N. Sakai and S. Matile, *Angew. Chem. Int. Ed.*, 2017, **56**, 812–815.

S2 S. Benz, M. Macchione, Q. Verolet, J. Mareda, N. Sakai, and S. Matile, *J. Am. Chem. Soc.*, 2016, **138**, 9093–9096.

S3 P. R. Ashton, U. Girreser, D. Giuffrida, F. H. Kohnke, J. P. Mathias, F. M. Raymo, A. M. Z. Slawin, J. F. Stoddart and D. J. Williams, *J. Am. Chem. Soc.*, 1993, **115**, 5422–5429.

S4 W. S. Li, D. L. Jiang, Y. Suna and T. Aida, *J. Am. Chem. Soc.*, 2005, **127**, 7700–7702.

S5 Y. Zhao, G. Huang, C. Besnard, J. Mareda, N. Sakai and S. Matile, *Chem. Eur. J.*, 2015, **21**, 6202–6207.

S6 Y. Zhao, C. Beuchat, J. Mareda, Y. Domoto, J. Gajewy, A. Wilson, N. Sakai, and S. Matile, *J. Am. Chem. Soc.*, 2014, **136**, 2101–2111.

S7 Gaussian 09, Revision **D.01**, M. J. Frisch, G. W. Trucks, H. B. Schlegel, G. E. Scuseria, M. A. Robb, J. R. Cheeseman, G. Scalmani, V. Barone, B. Mennucci, G. A. Petersson, H. Nakatsuji, M. Caricato, X. Li, H. P. Hratchian, A. F. Izmaylov, J. Bloino, G. Zheng, J. L. Sonnenberg, M. Hada, M. Ehara, K. Toyota, R. Fukuda, J. Hasegawa, M. Ishida, T. Nakajima, Y. Honda, O. Kitao, H. Nakai, T. Vreven, J. A. Montgomery, Jr., J. E. Peralta, F. Ogliaro, M. Bearpark, J. J. Heyd, E. Brothers, K. N. Kudin, V. N. Staroverov, T. Keith, R. Kobayashi, J. Normand, K. Raghavachari, A. Rendell, J. C. Burant, S. S. Iyengar, J. Tomasi, M. Cossi, N. Rega, J. M. Millam, M. Klene, J. E. Knox, J. B. Cross, V. Bakken, C. Adamo, J. Jaramillo, R. Gomperts, R. E. Stratmann, O. Yazyev, A. J. Austin, R. Cammi, C. Pomelli, J. W. Ochterski, R. L. Martin, K. Morokuma, V. G. Zakrzewski, G. A. Voth, P. Salvador, J. J. Dannenberg, S. Dapprich, A. D. Daniels, O. Farkas, J. B. Foresman, J. V. Ortiz, J. Cioslowski and D. J. Fox, Gaussian, Inc., Wallingford CT, **2013**.

S8 (*a*) Y. Zhao and D. G. Truhlar, *J. Chem. Phys.,* 2006*,* **125***,* 194101–19411; (*b*) Y. Zhao and D. G. Truhlar, *Theor. Chem. Acc.*, 2008, **120**, 215–241.

S9 W. Humphrey, A. Dalke and K. Schulten, *J. Mol. Graphics Modell.*, 1996, **14**, 33–38.

S10 Mercury CSD 2.0, C. F. Macrae, I. J. Bruno, J. A. Chisholm, P. R. Edgington, P. McCabe, E. Pidcock, L. Rodriguez-Monge, R. Taylor, J. van de Streek and P. A. Wood, *J. Appl. Cryst*., 2008, **41**, 466-470.

9. HRMS spectra

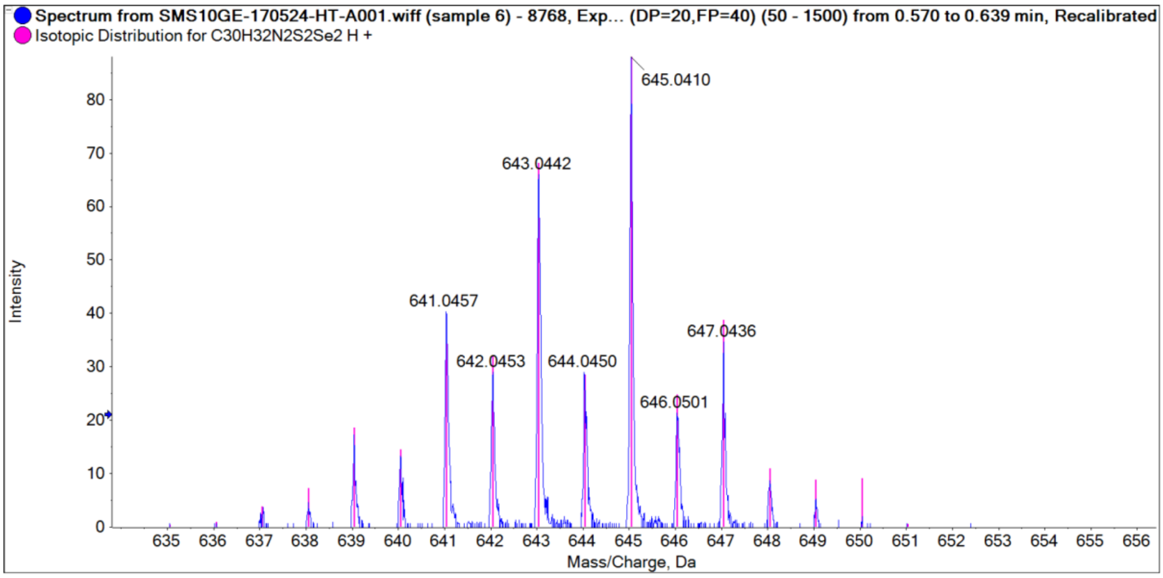

Fig. S10 Measured HRMS (ESI, $CHCl_3$/MeOH 1:1, +ve) spectra of **5** (blue), and calculated spectra (pink) for $[M+H]^+$.

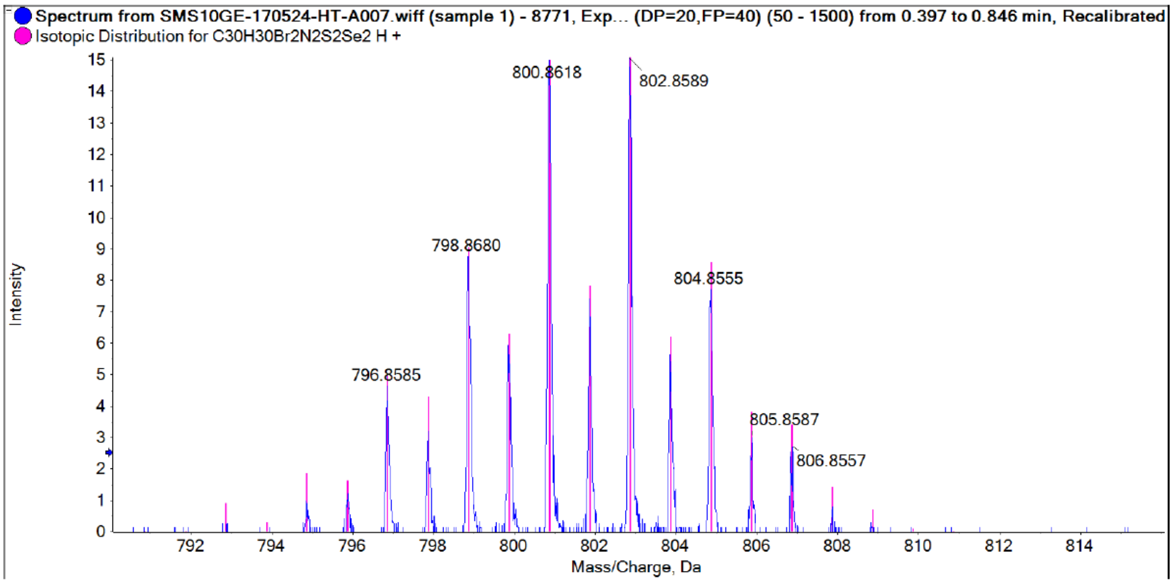

Fig. S11 Measured HRMS (ESI, $CHCl_3$/MeOH 1:1, +ve) spectra of **21** (blue), and calculated spectra (pink) for $[M+H]^+$.

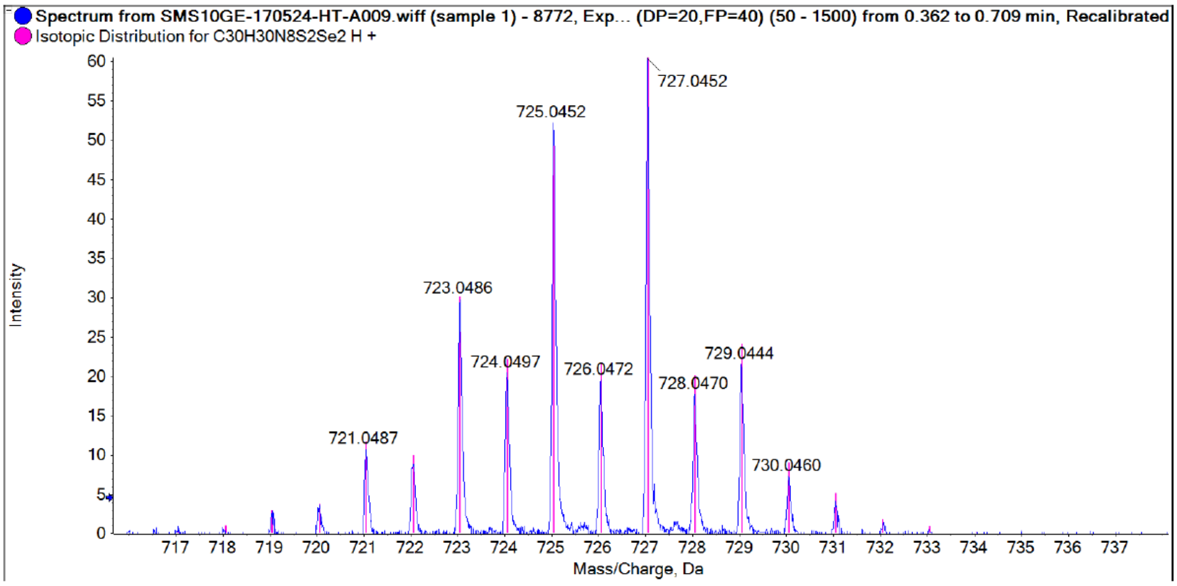

Fig. S12 Measured HRMS (ESI, $CHCl_3$/MeOH 1:1, +ve) spectra of **22** (blue), and calculated spectra (pink) for $[M+H]^+$.

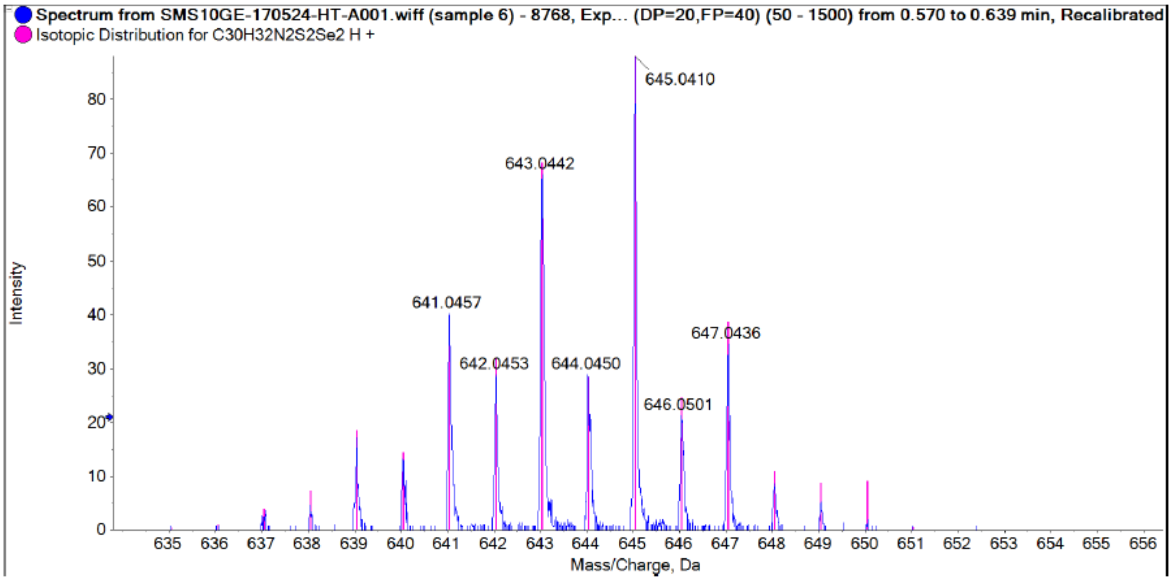

Fig. S13 Measured HRMS (ESI, $CHCl_3$/MeOH 1:1, +ve) spectra of **1** (blue), and calculated spectra (pink) for $[M+H]^+$.

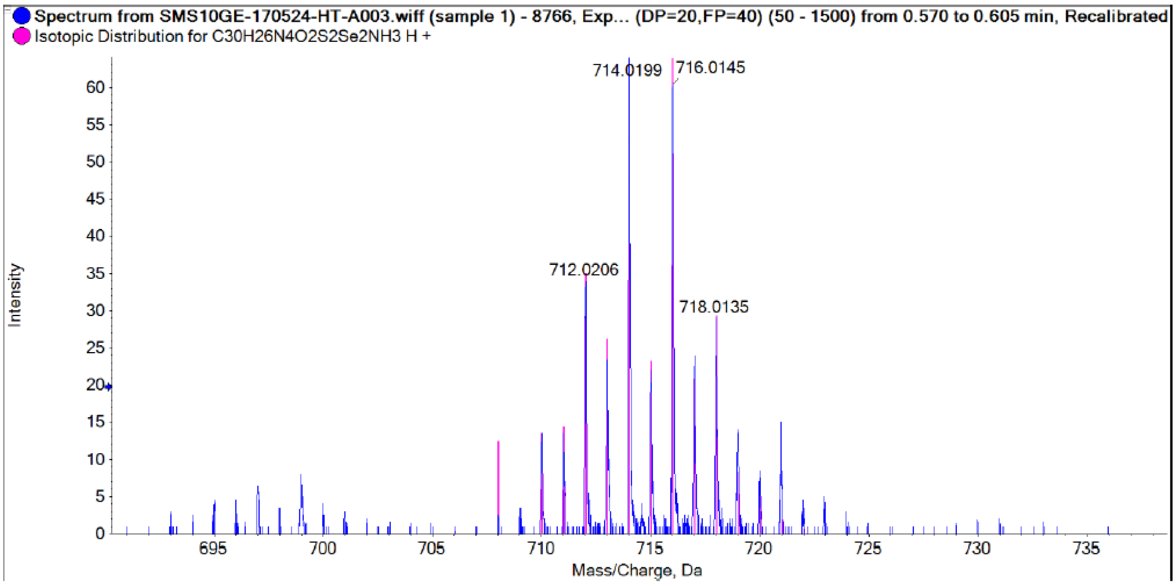

Fig. S14 Measured HRMS (ESI, $CHCl_3$/MeOH 1:1, +ve) spectra of **2 Chiral** (blue), and calculated spectra (pink) for $[M+H]^+$.

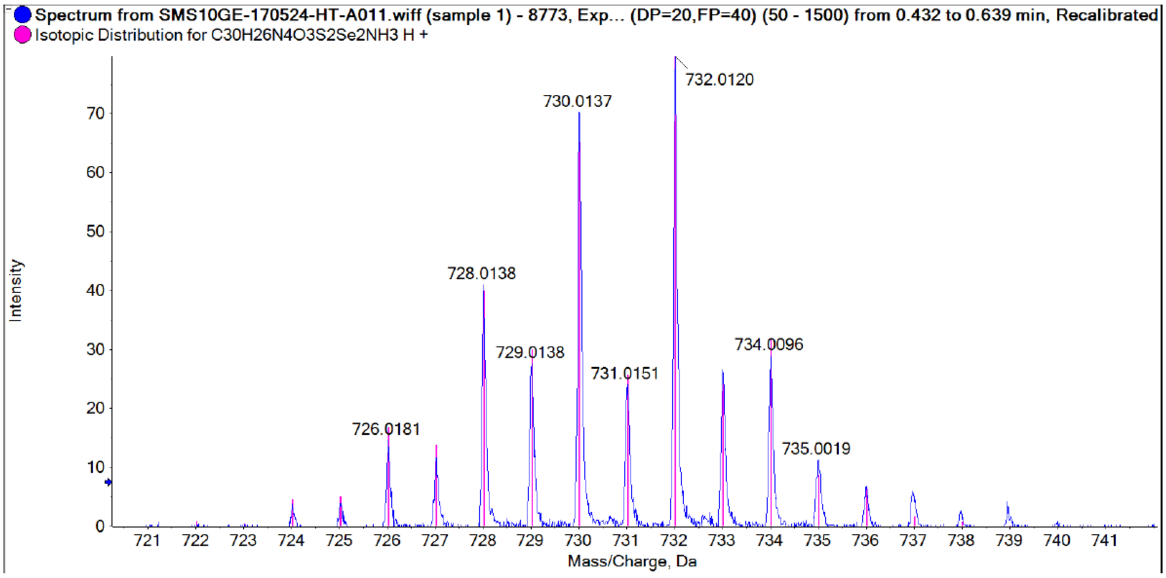

Fig. S15 Measured HRMS (ESI, $CHCl_3$/MeOH 1:1, +ve) spectra of **3** (blue), and calculated spectra (pink) for $[M+H]^+$.

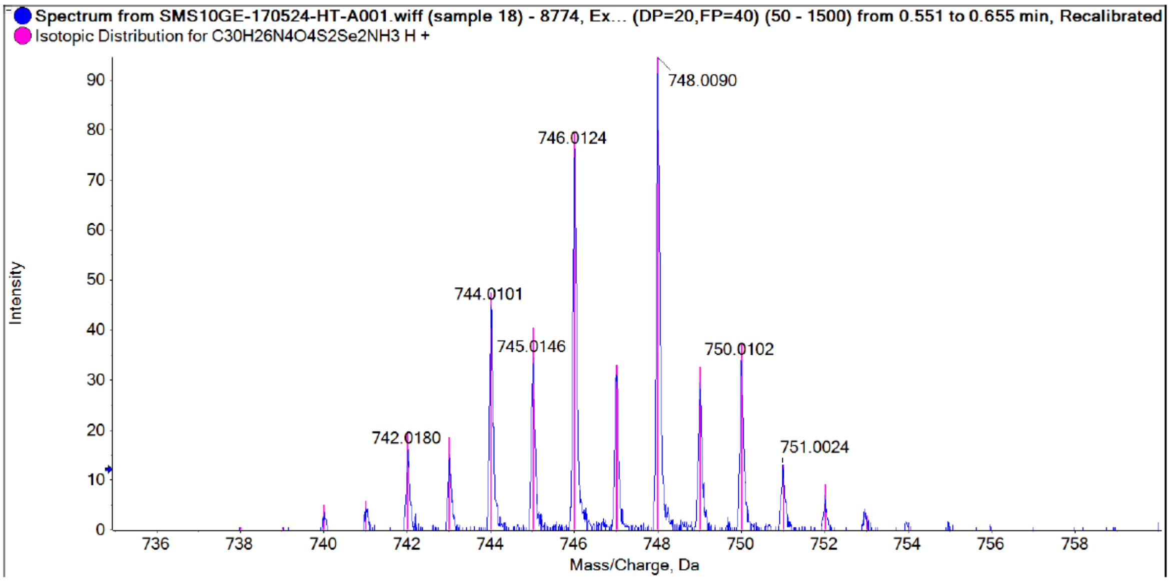

Fig. S16 Measured HRMS (ESI, $CHCl_3$/MeOH 1:1, +ve) spectra of **4** (blue), and calculated spectra (pink) for $[M+H]^+$.

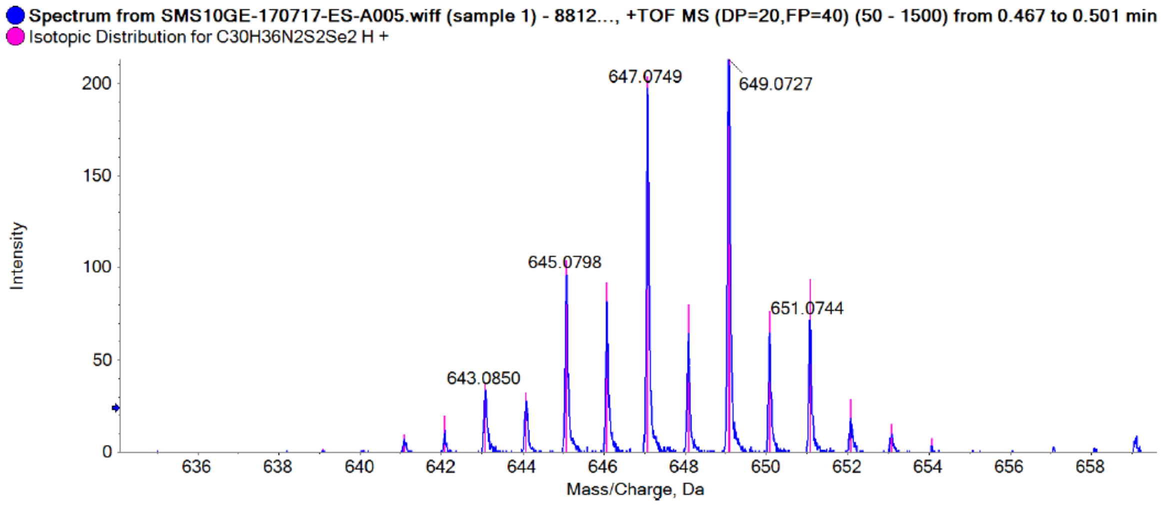

Fig. S17 Measured HRMS (ESI, $CHCl_3$/MeOH 1:1, +ve) spectra of **7** (blue), and calculated spectra (pink) for $[M+H]^+$.

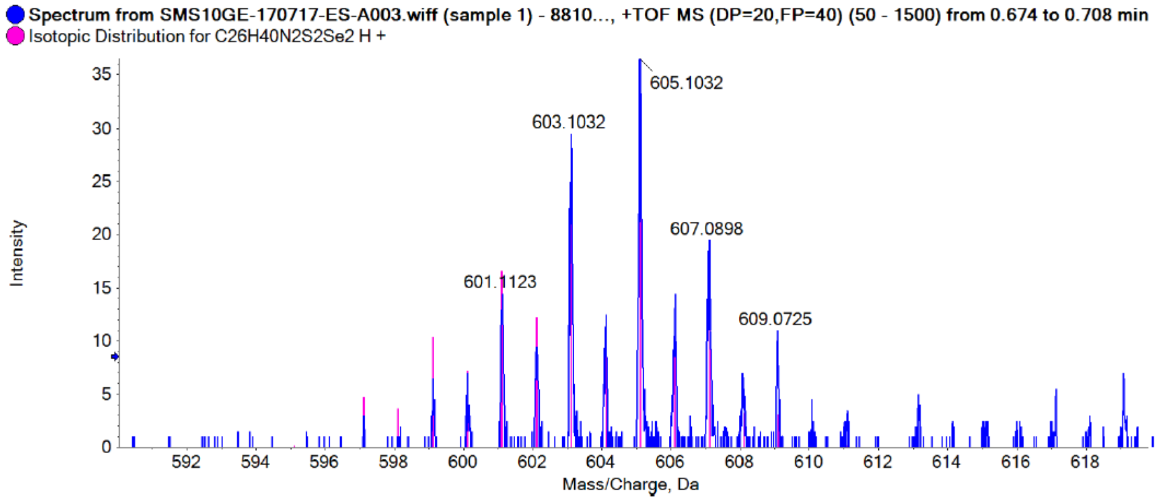

Fig. S18 Measured HRMS (ESI, $CHCl_3$/MeOH 1:1, +ve) spectra of **10** (blue), and calculated spectra (pink) for $[M+H]^+$.

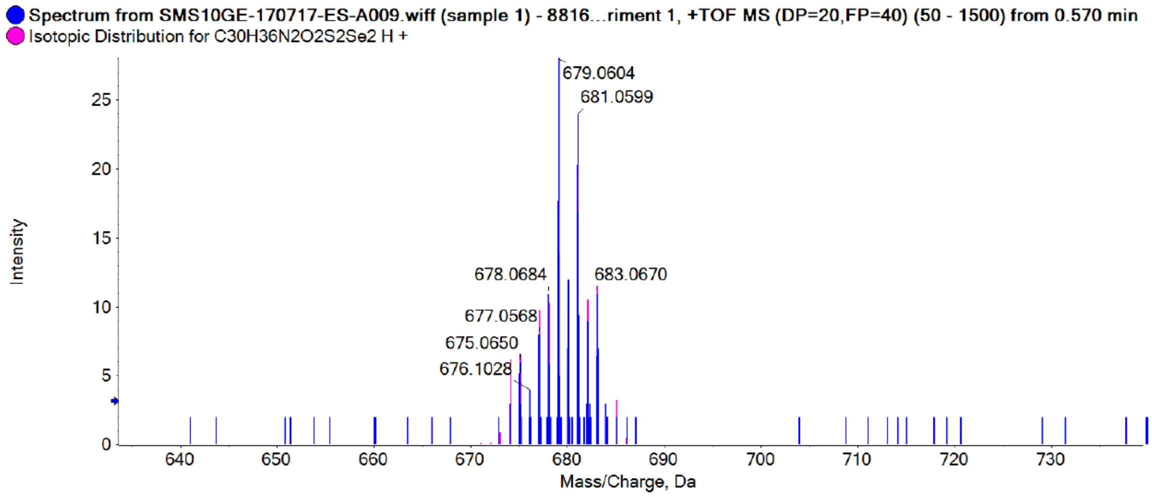

Fig. S19 Measured HRMS (ESI, $CHCl_3$/MeOH 1:1, +ve) spectra of **8** (blue), and calculated spectra (pink) for $[M+H]^+$.

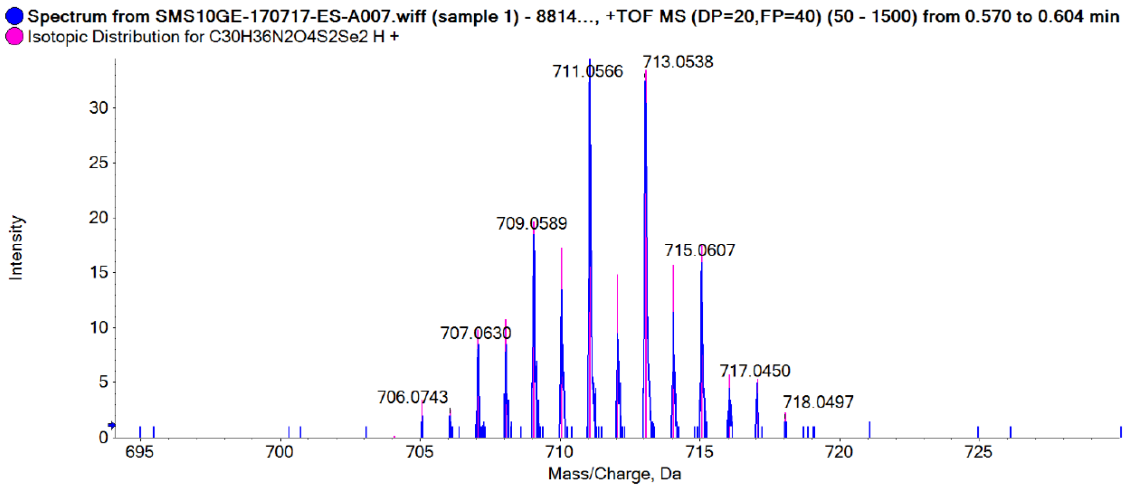

Fig. S20 Measured HRMS (ESI, $CHCl_3$/MeOH 1:1, +ve) spectra of **9** (blue), and calculated spectra (pink) for $[M+H]^+$.

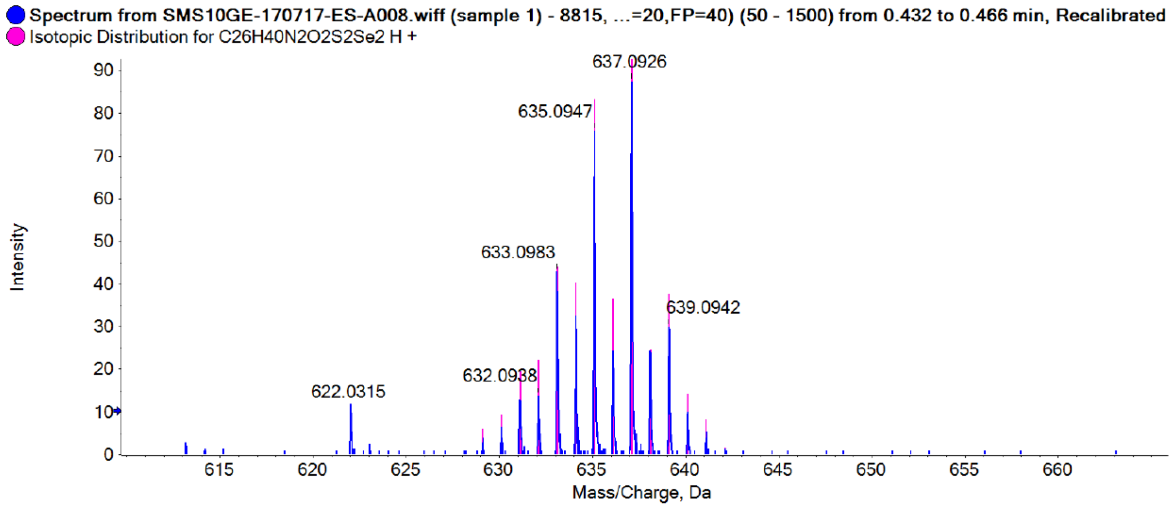

Fig. S21 Measured HRMS (ESI, $CHCl_3$/MeOH 1:1, +ve) spectra of **11** (blue), and calculated spectra (pink) for $[M+H]^+$.

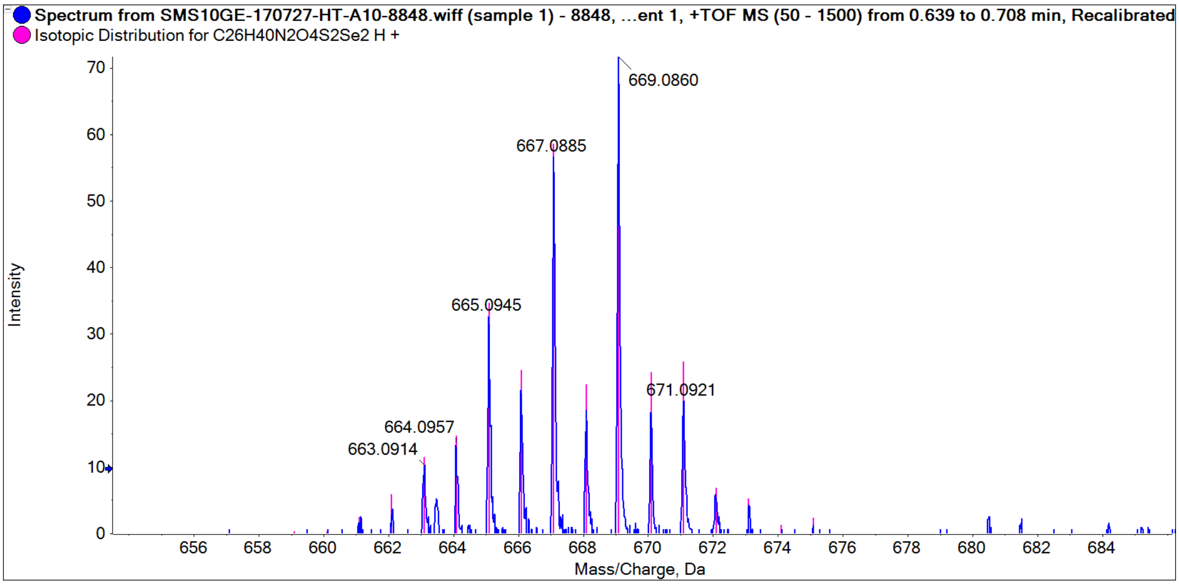

Fig. S22 Measured HRMS (ESI, $CHCl_3$/MeOH 1:1, +ve) spectra of **12** (blue), and calculated spectra (pink) for $[M+H]^+$.

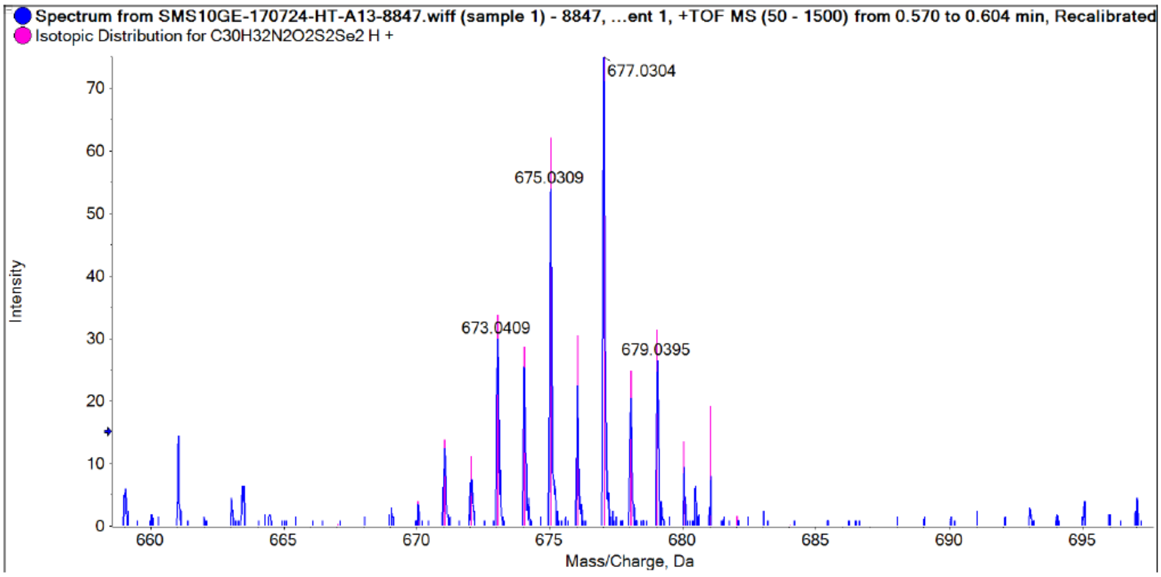

Fig. S23 Measured HRMS (ESI, $CHCl_3$/MeOH 1:1, +ve) spectra of **6** (blue), and calculated spectra (pink) for $[M+H]^+$.

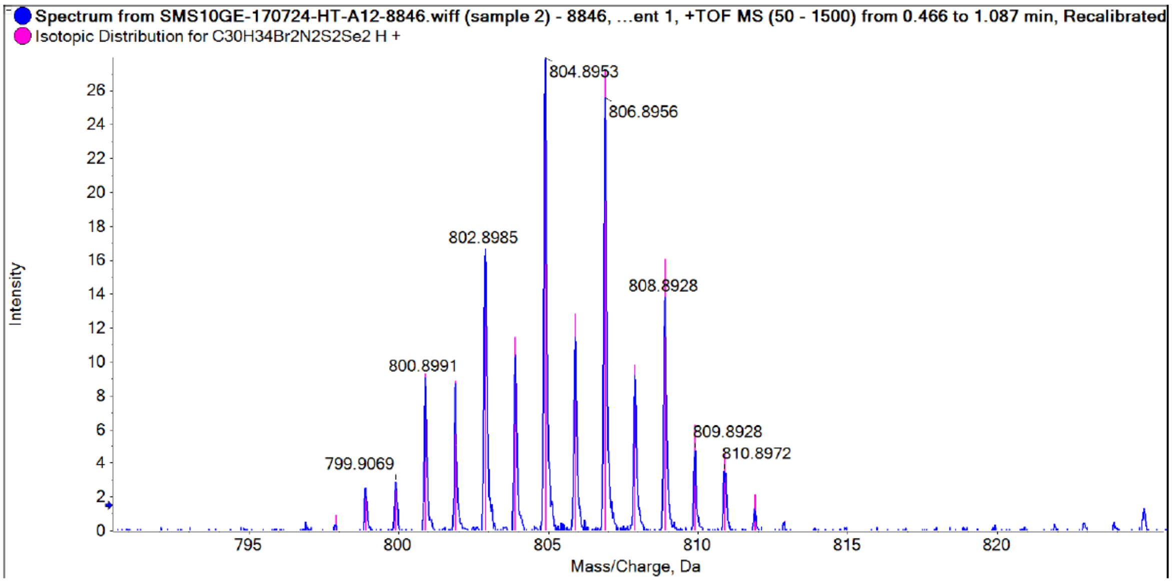

Fig. S24 Measured HRMS (ESI, $CHCl_3$/MeOH 1:1, +ve) spectra of **27** (blue), and calculated spectra (pink) for $[M+H]^+$.

10. NMR spectra

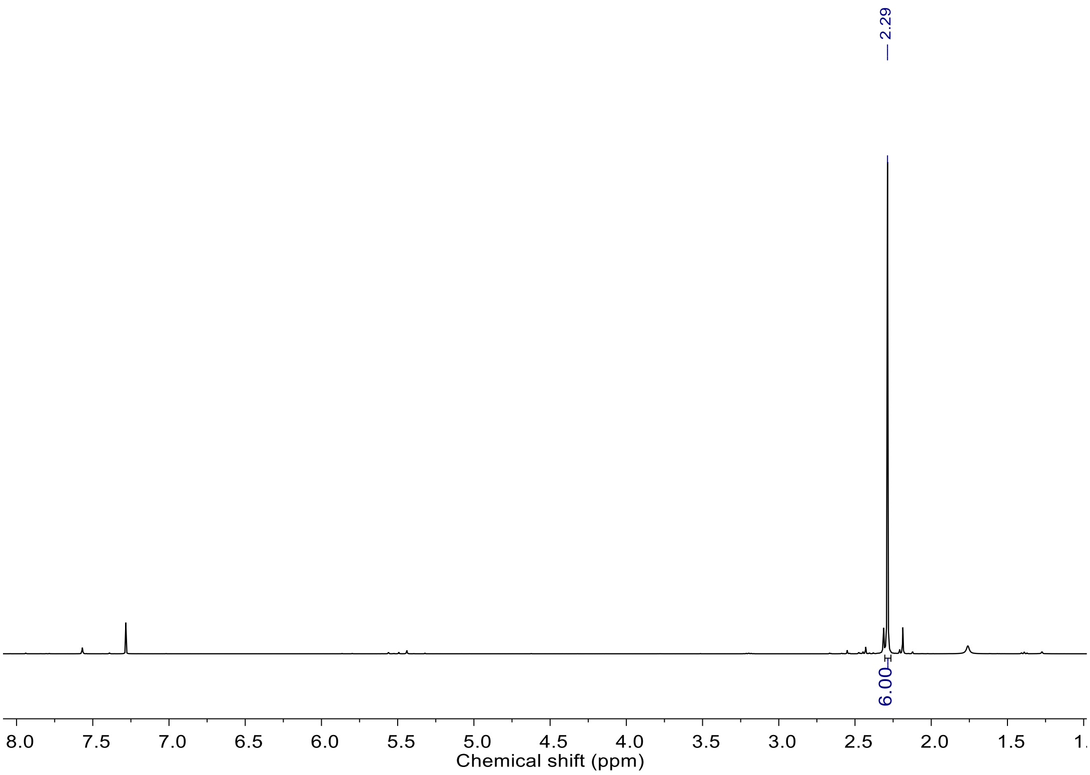

Fig. S25 ^{1}H NMR spectrum of **16** in $CDCl_3$.

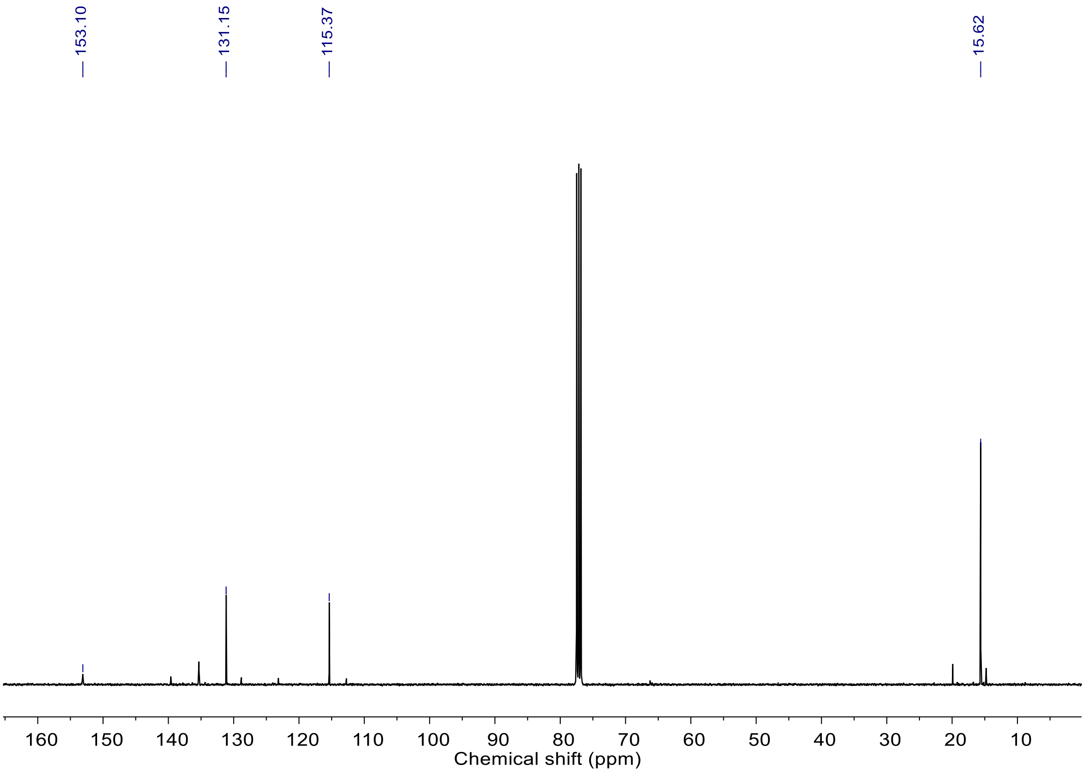

Fig. S26 ^{13}C NMR spectrum of **16** in $CDCl_3$.

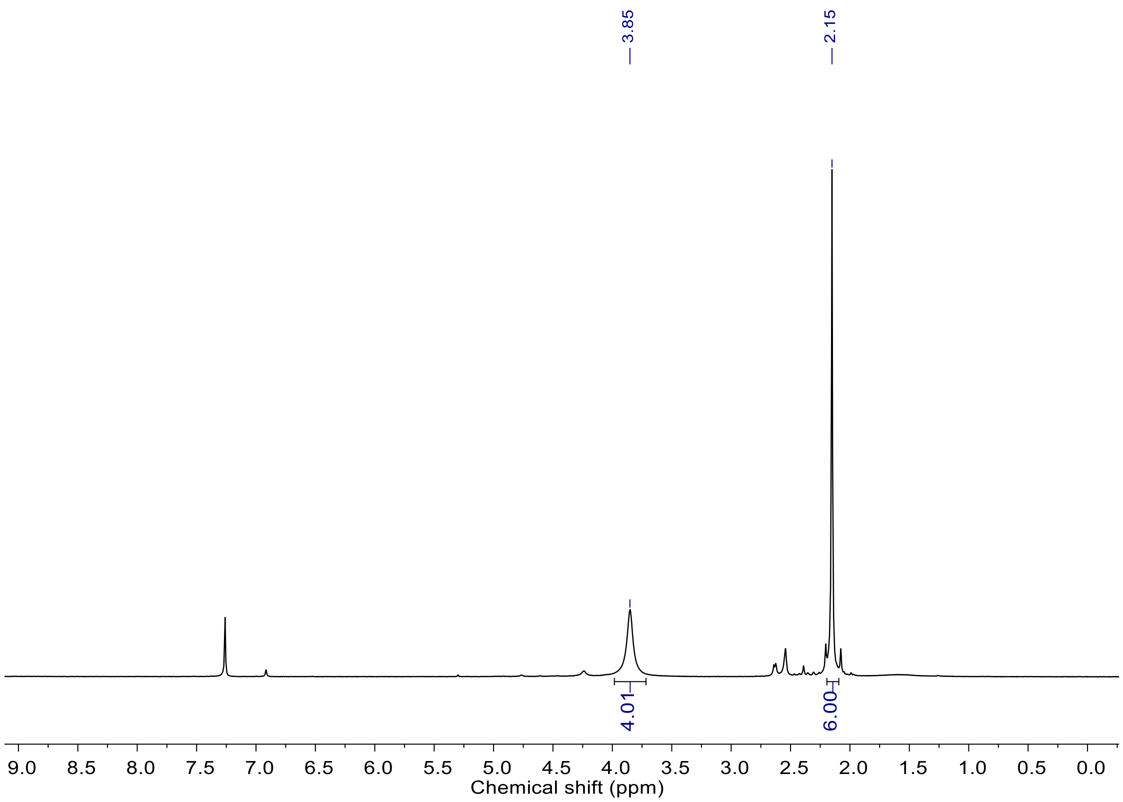

Fig. S27 ^{1}H NMR spectrum of **17** in $CDCl_3$.

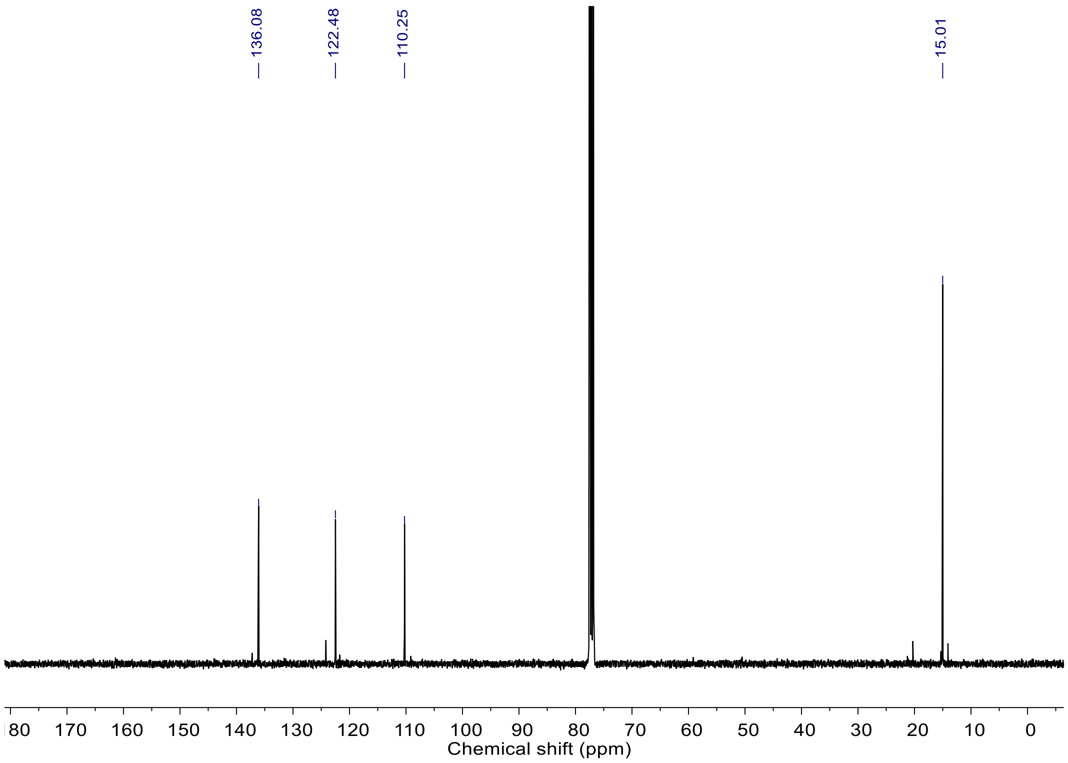

Fig. S28 ^{13}C NMR spectrum of **17** in $CDCl_3$.

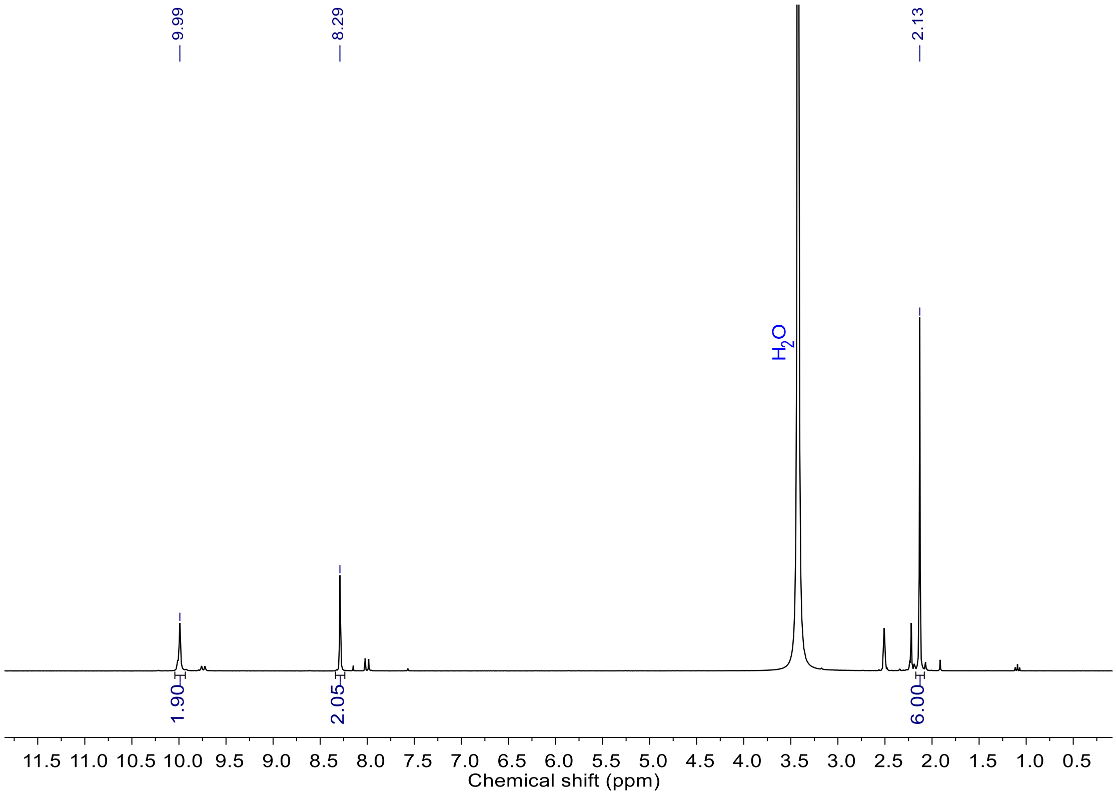

Fig. S29 ^{1}H NMR spectrum of **18** in DMSO-d_6.

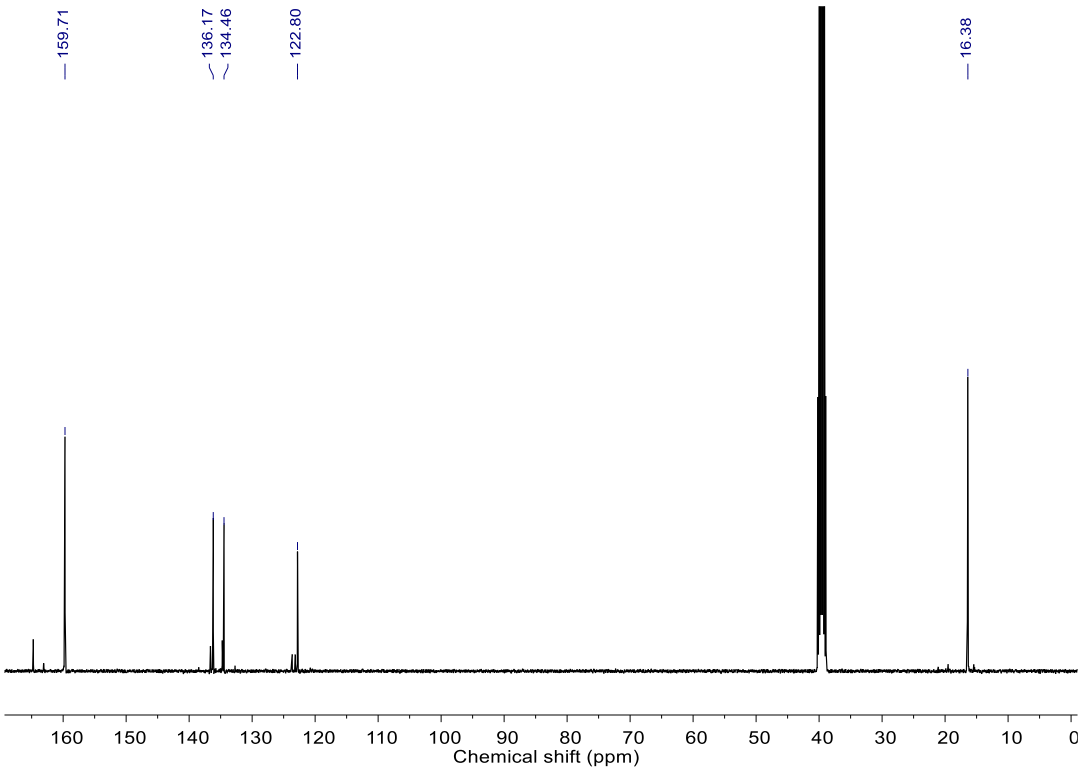

Fig. S30 ^{13}C NMR spectrum of **18** in DMSO-d_6.

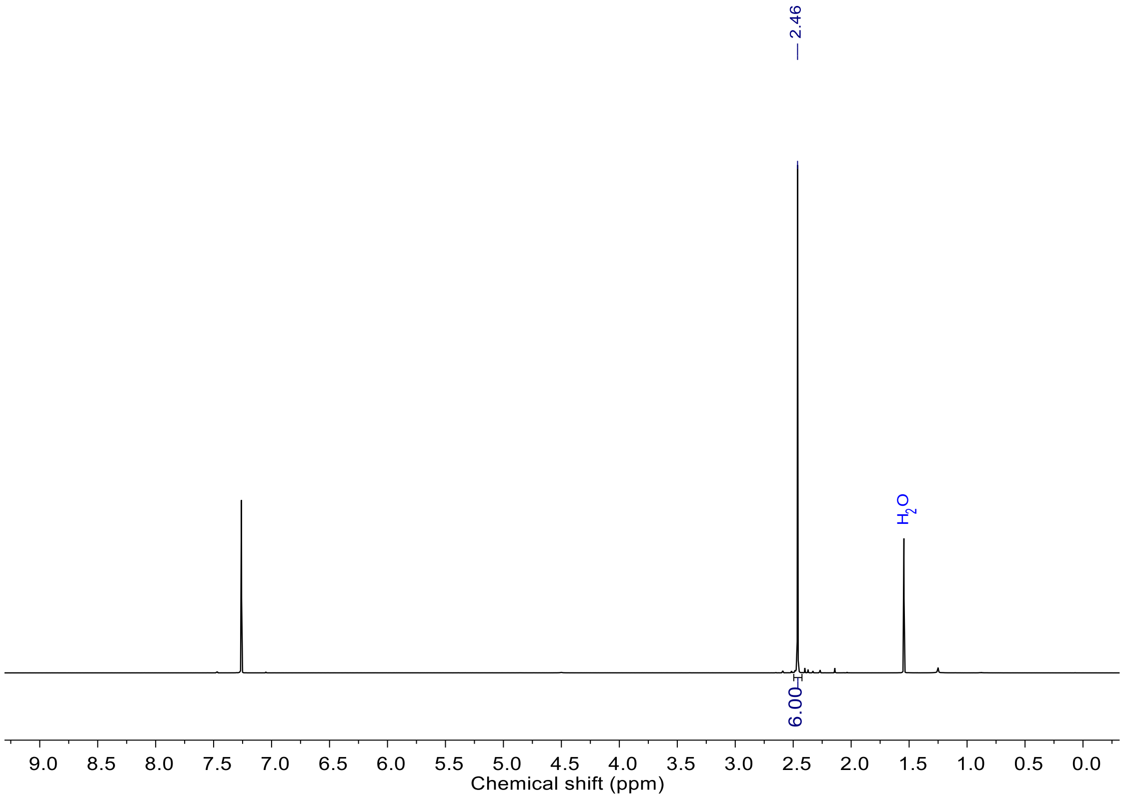

Fig. S31 ^{1}H NMR spectrum of **19** in $CDCl_3$.

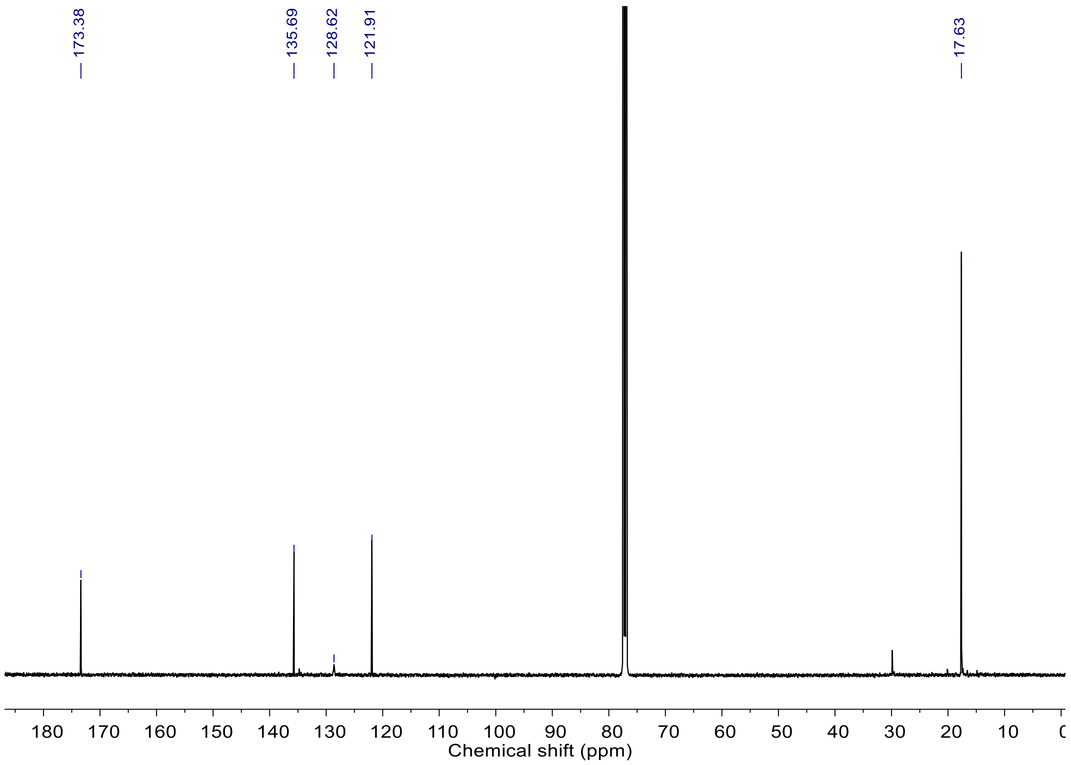

Fig. S32 ^{13}C NMR spectrum of **19** in $CDCl_3$.

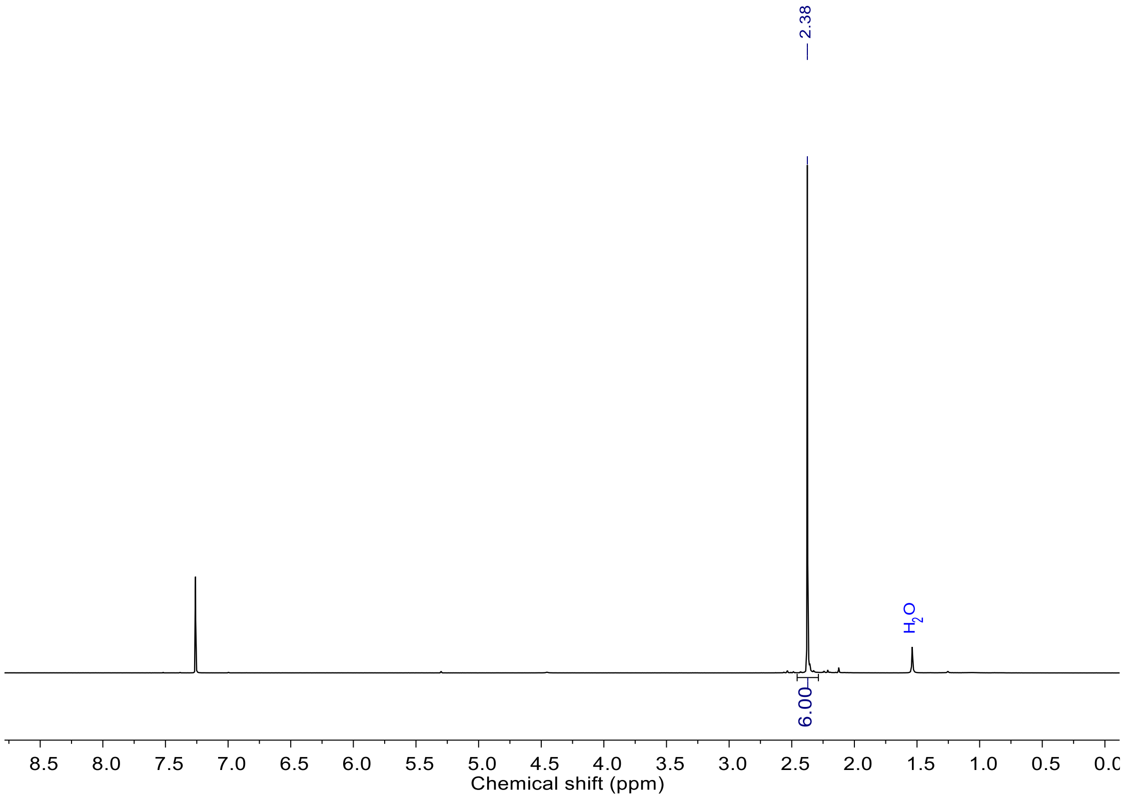

Fig. S33 ^{1}H NMR spectrum of **20** in $CDCl_3$.

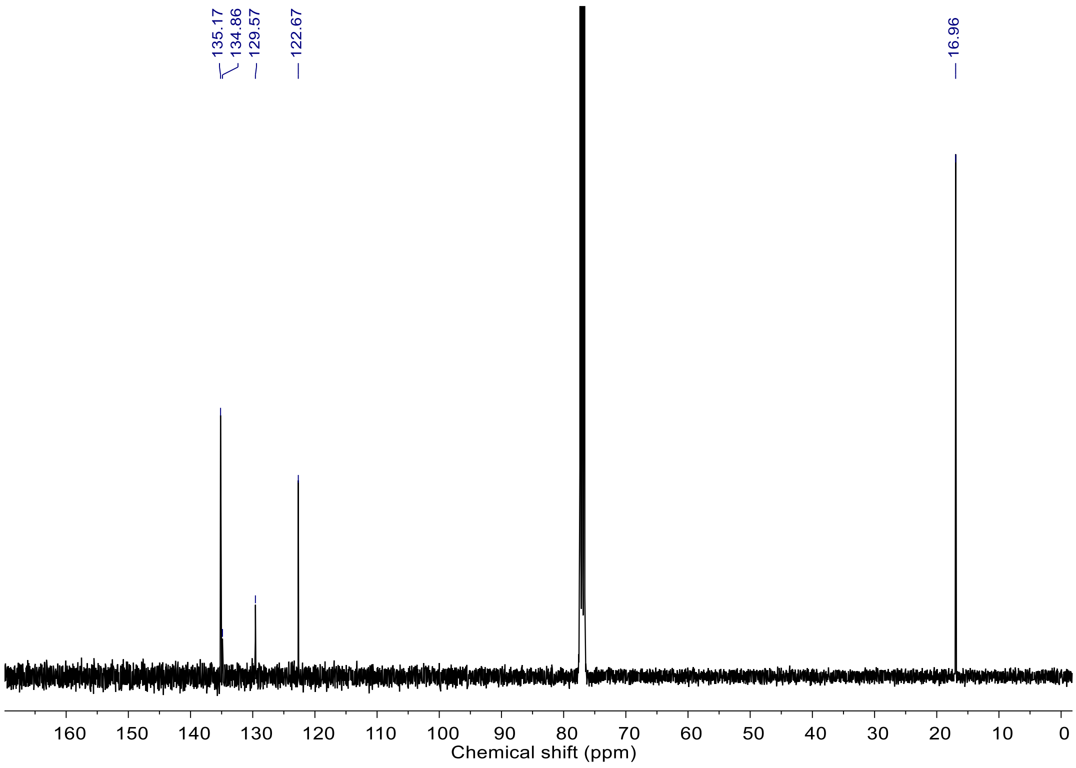

Fig. S34 ^{13}C NMR spectrum of **20** in $CDCl_3$.

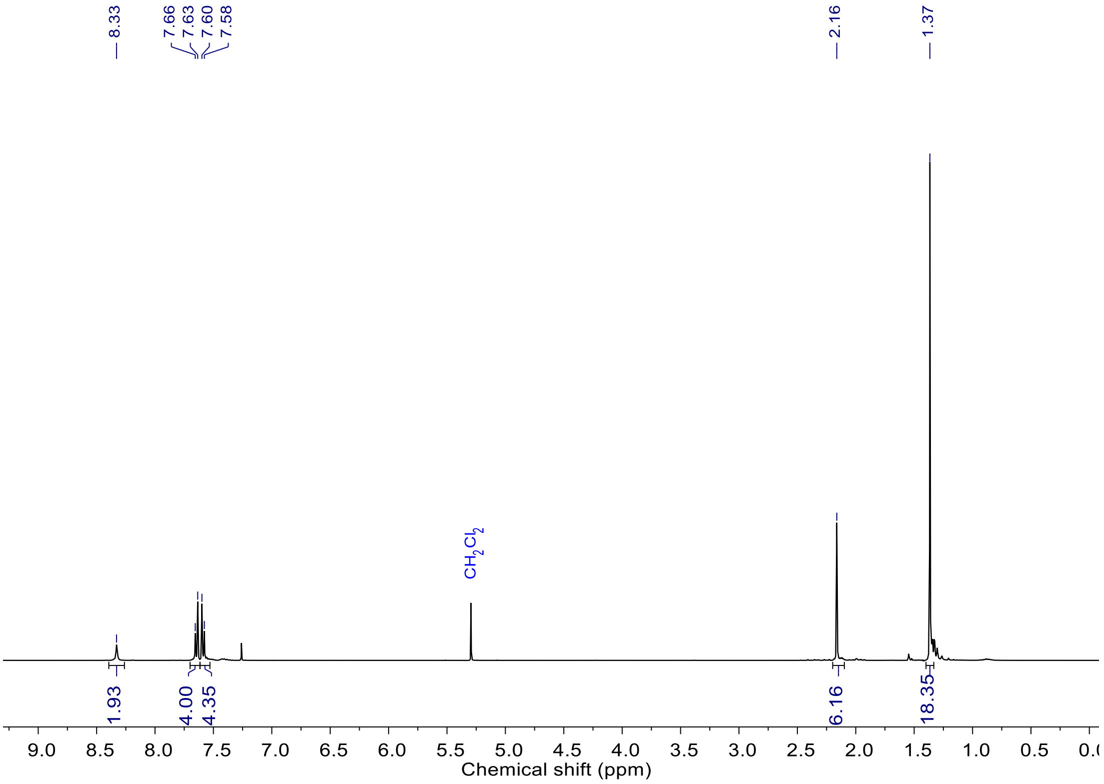

Fig. S35 ^{1}H NMR spectrum of **27** in $CDCl_3$.

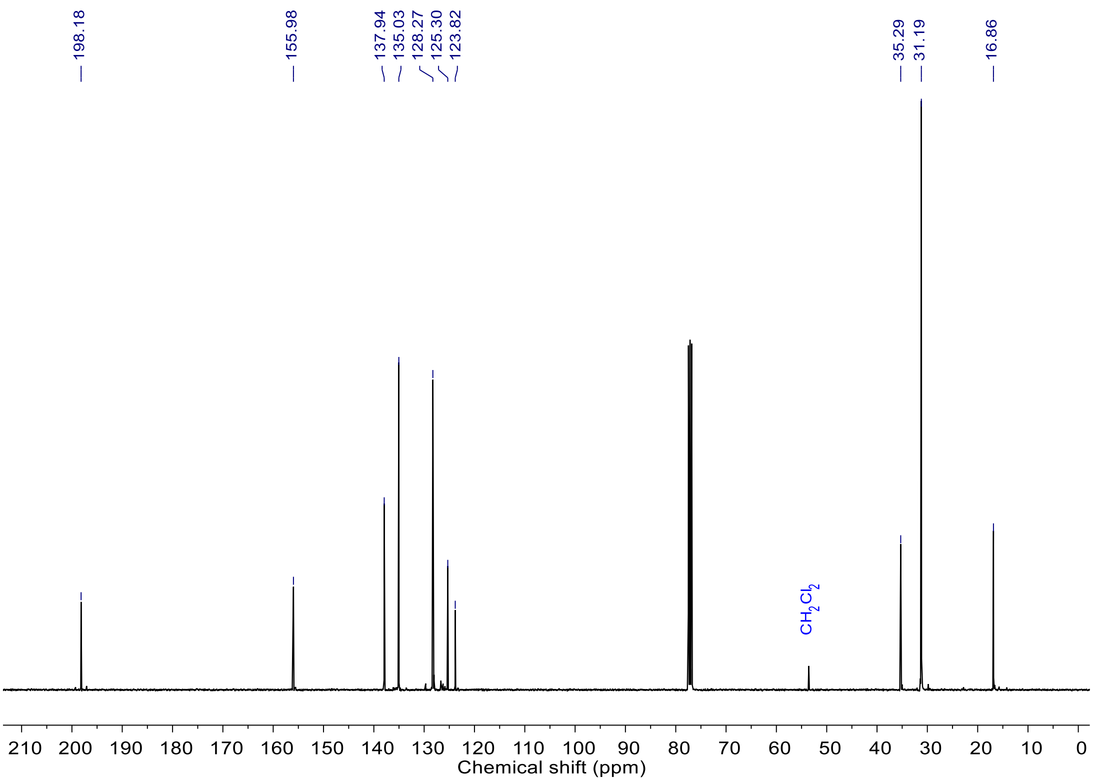

Fig. S36 ^{13}C NMR spectrum of **27** in $CDCl_3$.

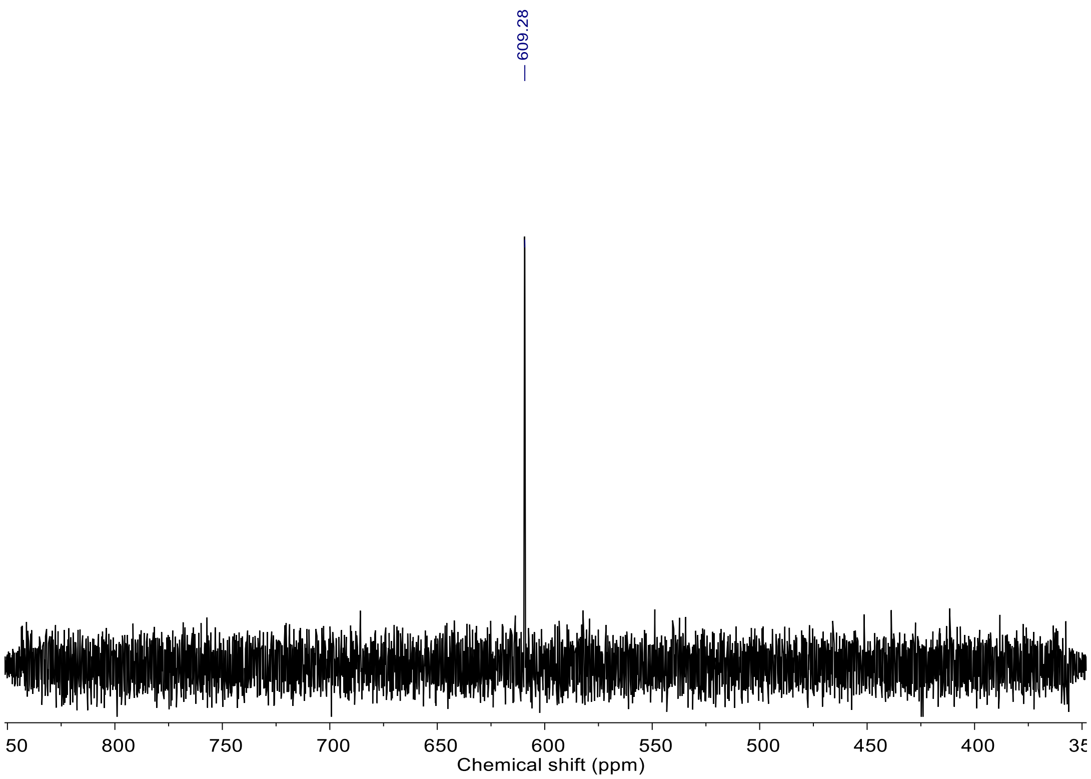

Fig. S37 ^{77}Se NMR spectrum of **27** in $CDCl_3$.

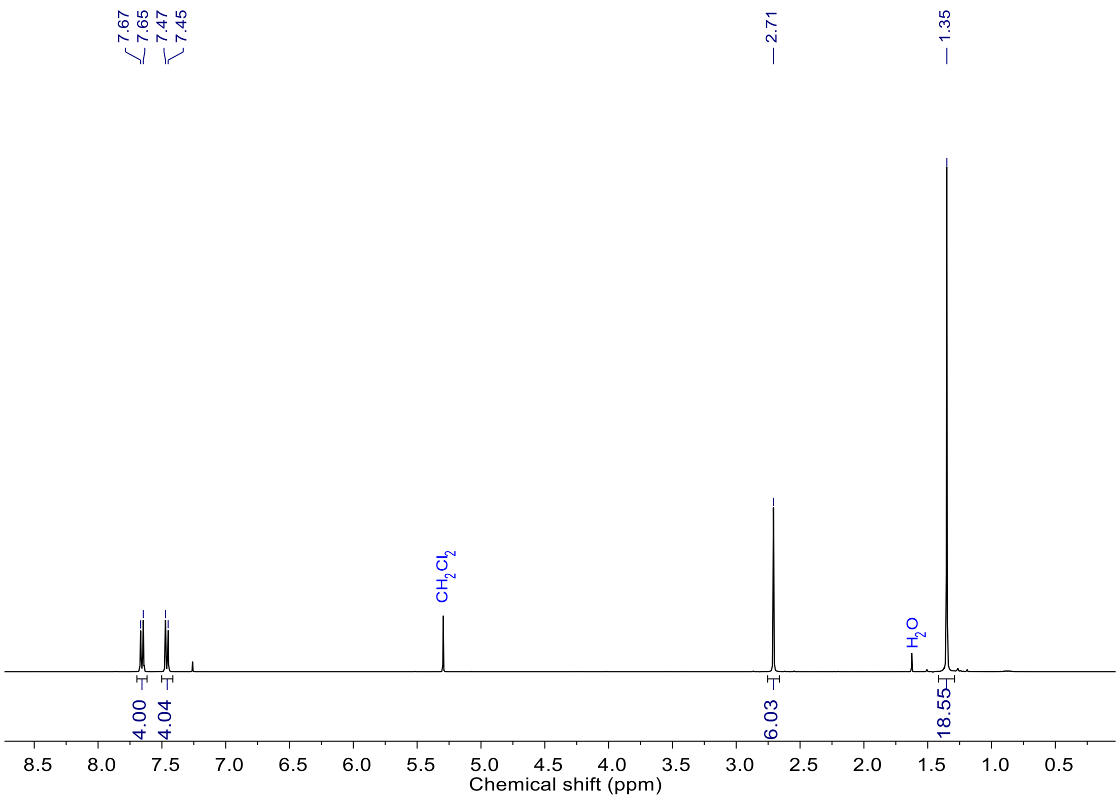

Fig. S38 ^{1}H NMR spectrum of **5** in $CDCl_3$.

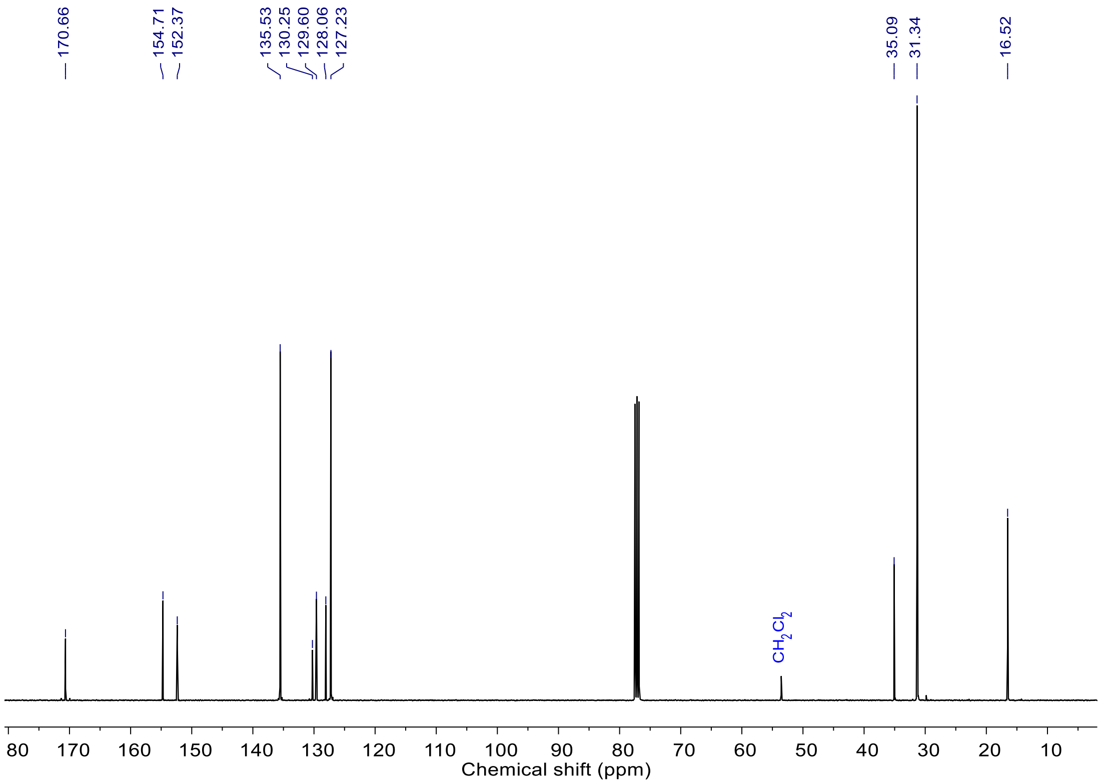

Fig. S39 ^{13}C NMR spectrum of **5** in $CDCl_3$.

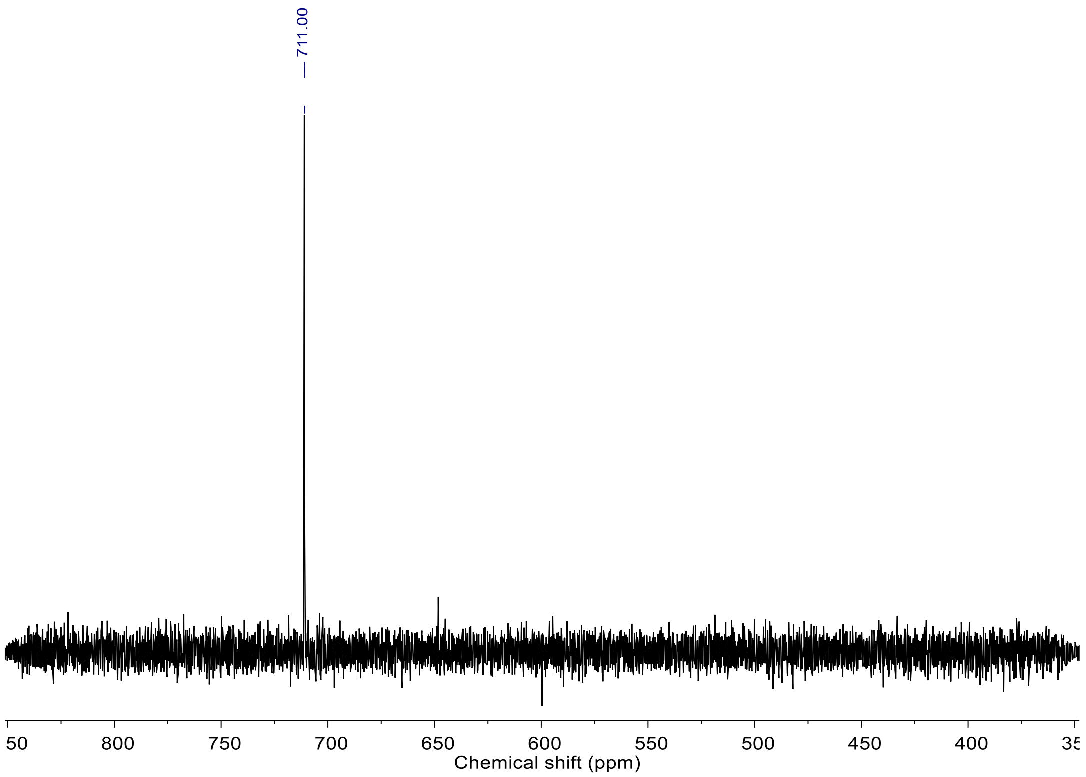

Fig. S40 ^{77}Se NMR spectrum of **5** in $CDCl_3$.

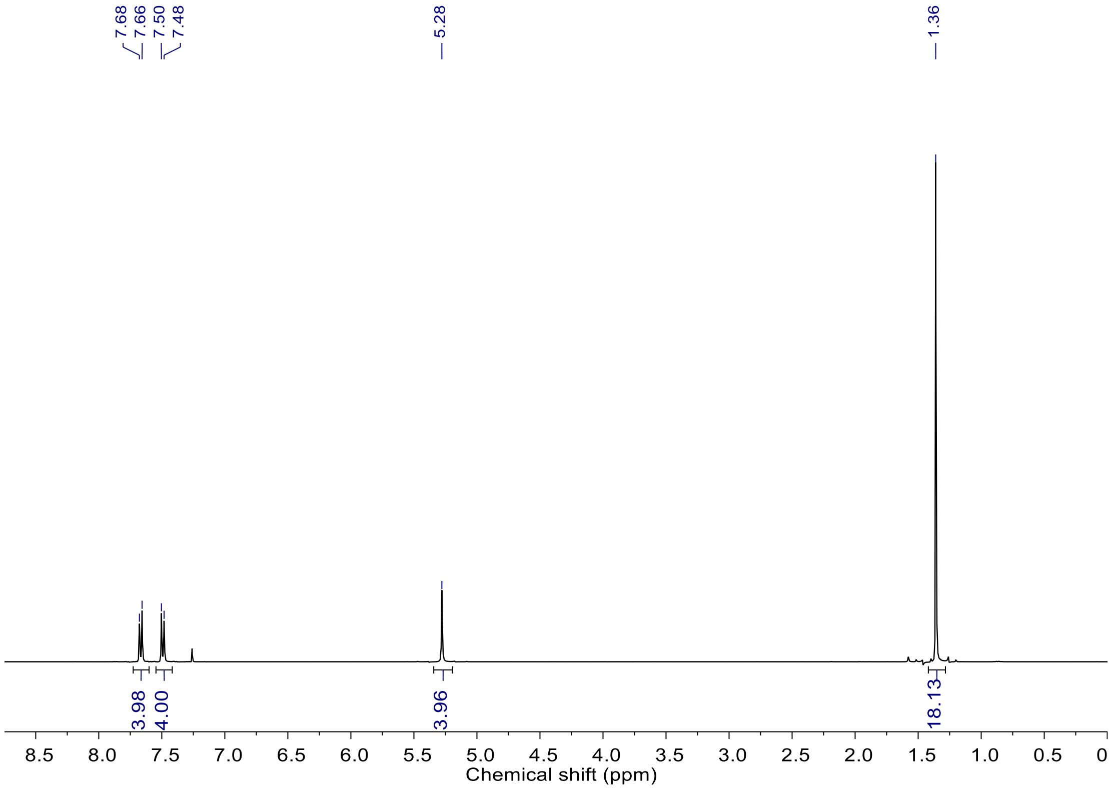

Fig. S41 ¹H NMR spectrum of **21** in $CDCl_3$.

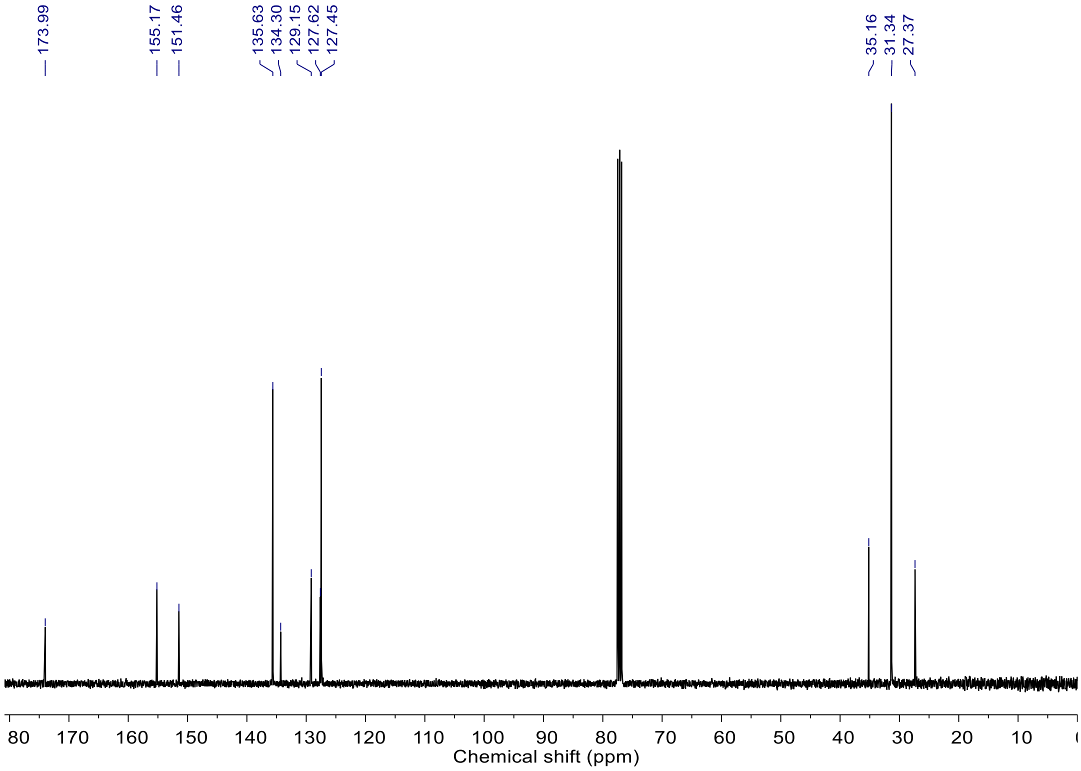

Fig. S42 ¹³C NMR spectrum of **21** in $CDCl_3$.

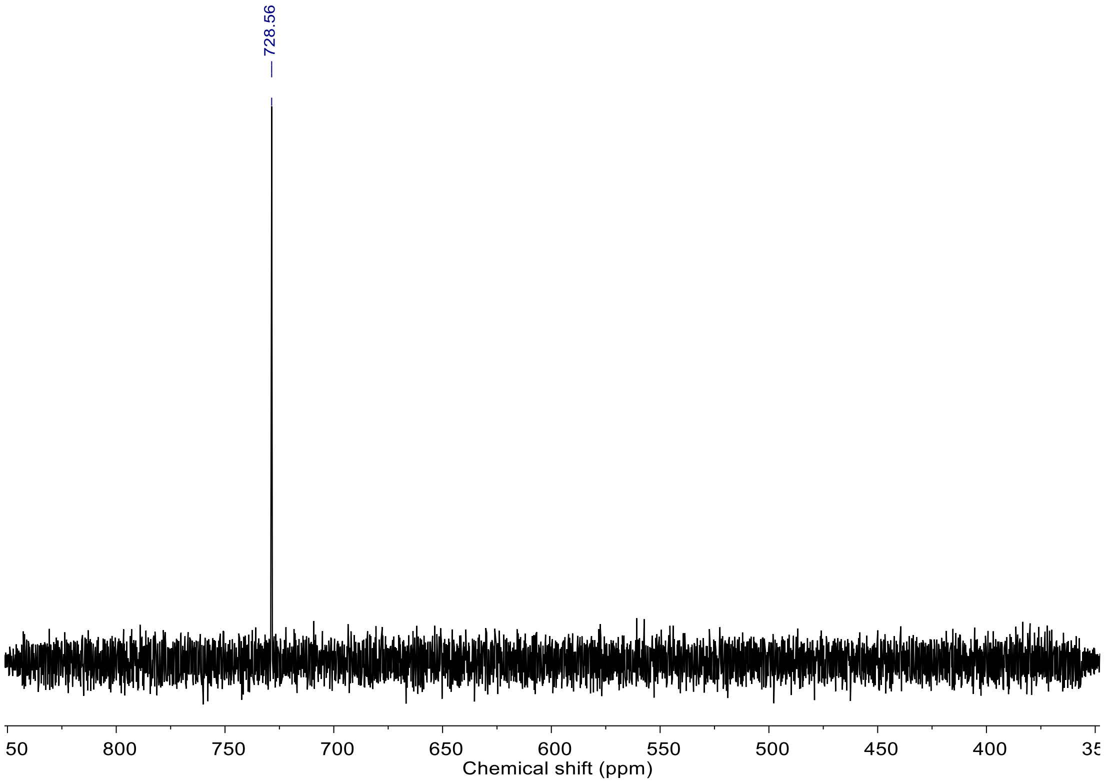

Fig. S43 ^{77}Se NMR spectrum of **21** in $CDCl_3$.

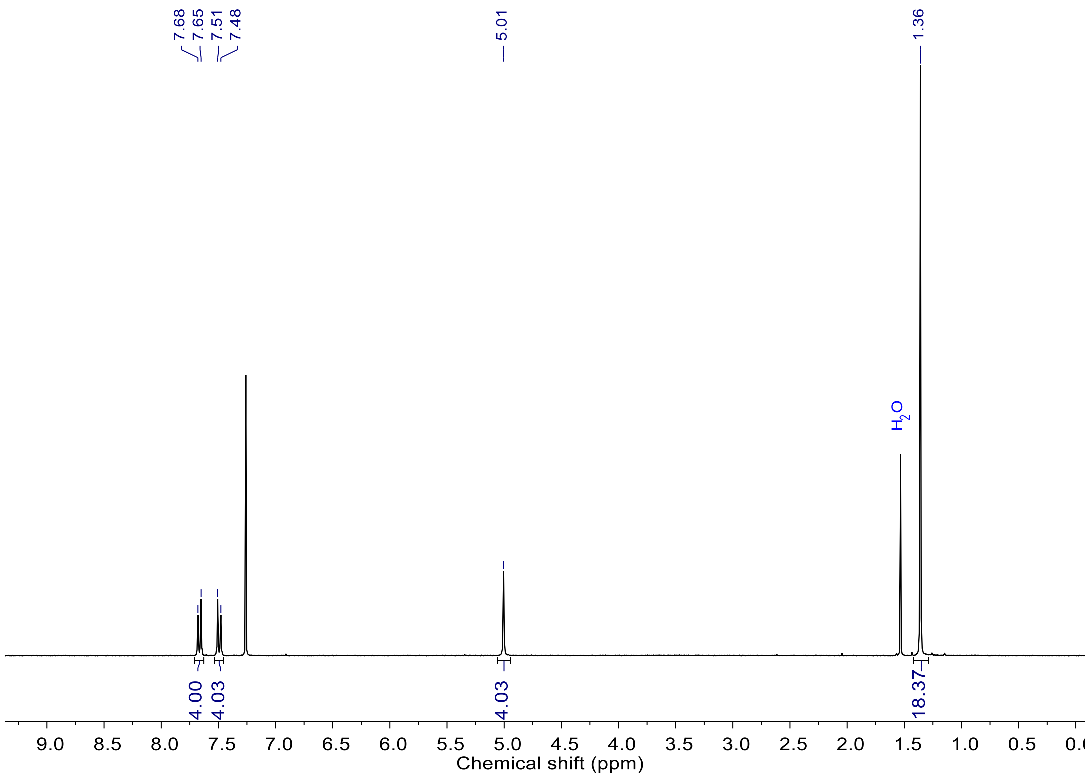

Fig. S44 ^{1}H NMR spectrum of **22** in $CDCl_3$.

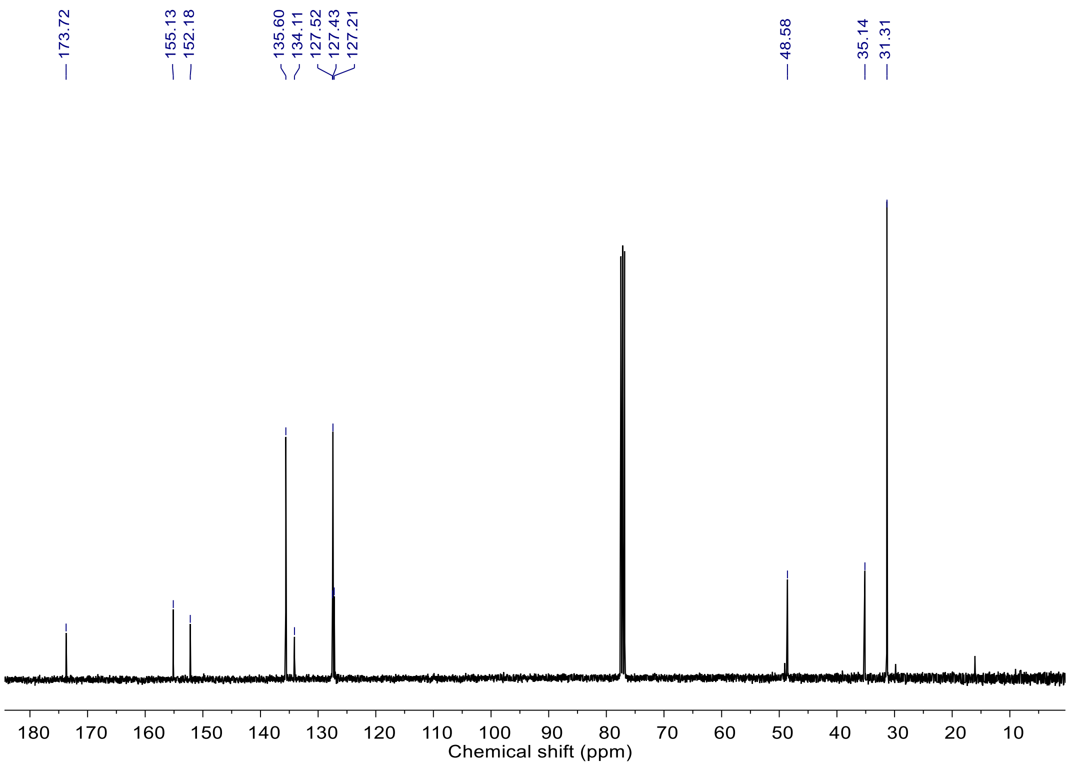

Fig. S45 ^{13}C NMR spectrum of **22** in $CDCl_3$.

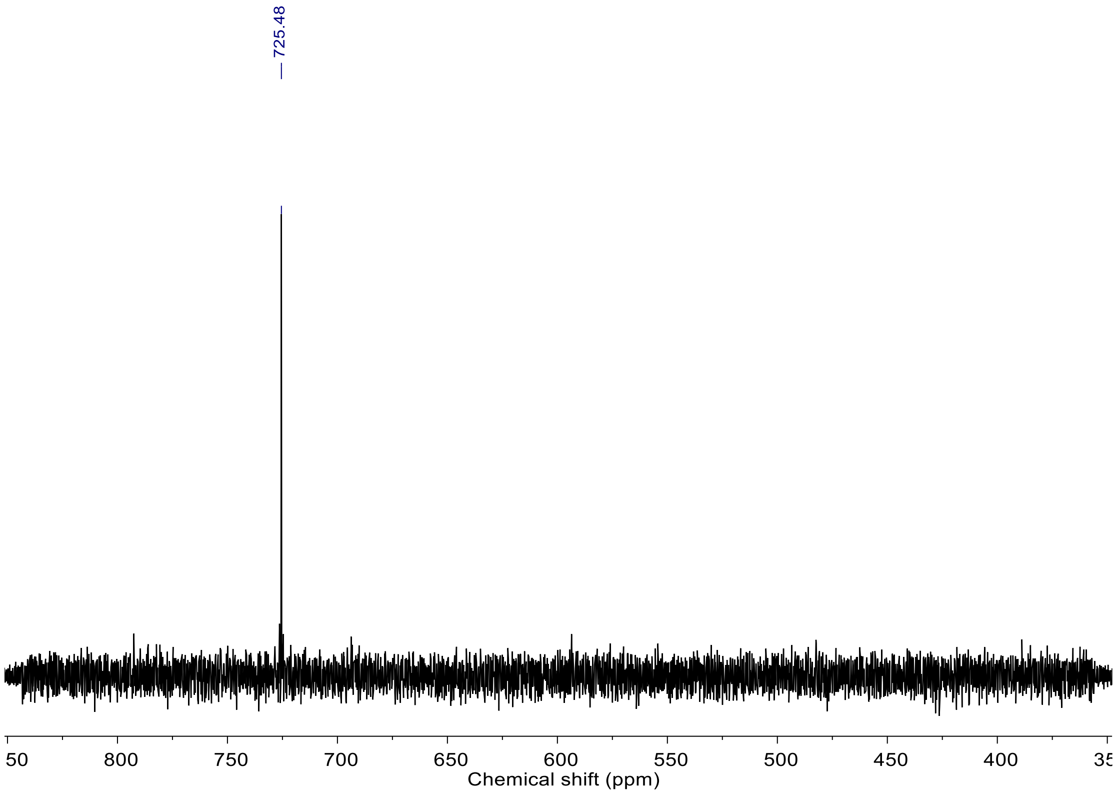

Fig. S46 ^{77}Se NMR spectrum of **22** in $CDCl_3$.

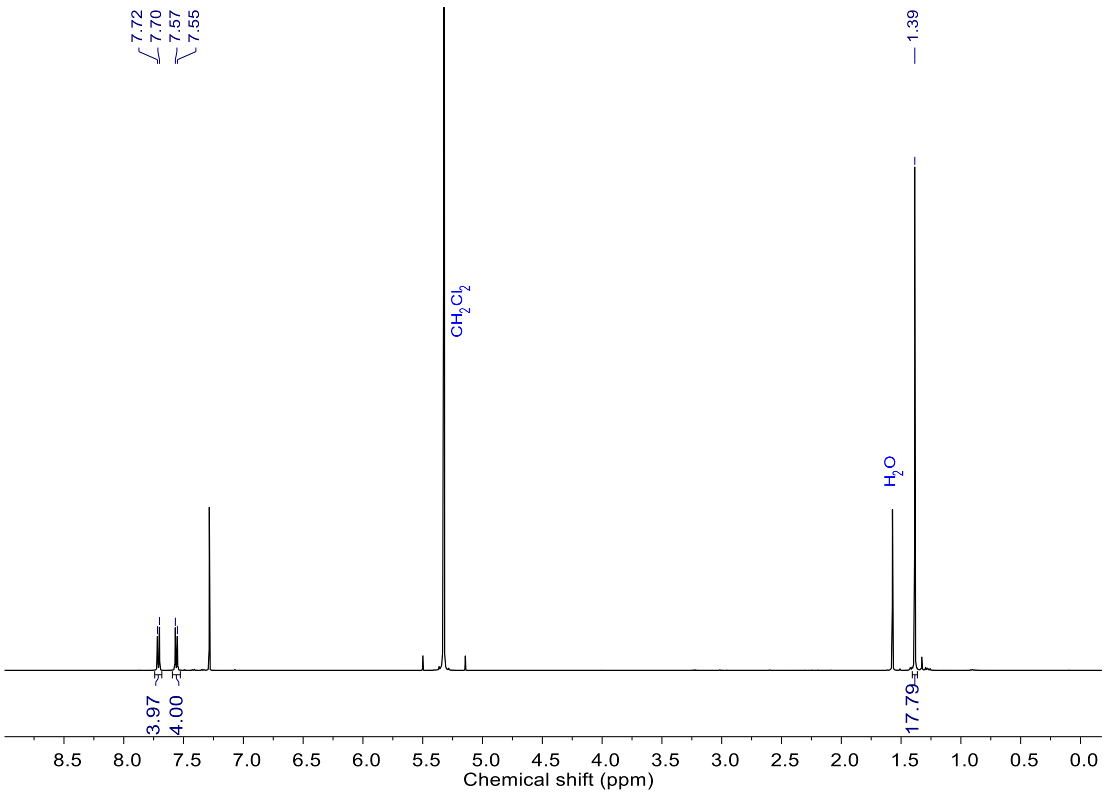

Fig. S47 ^{1}H NMR spectrum of **1** in $CDCl_3$.

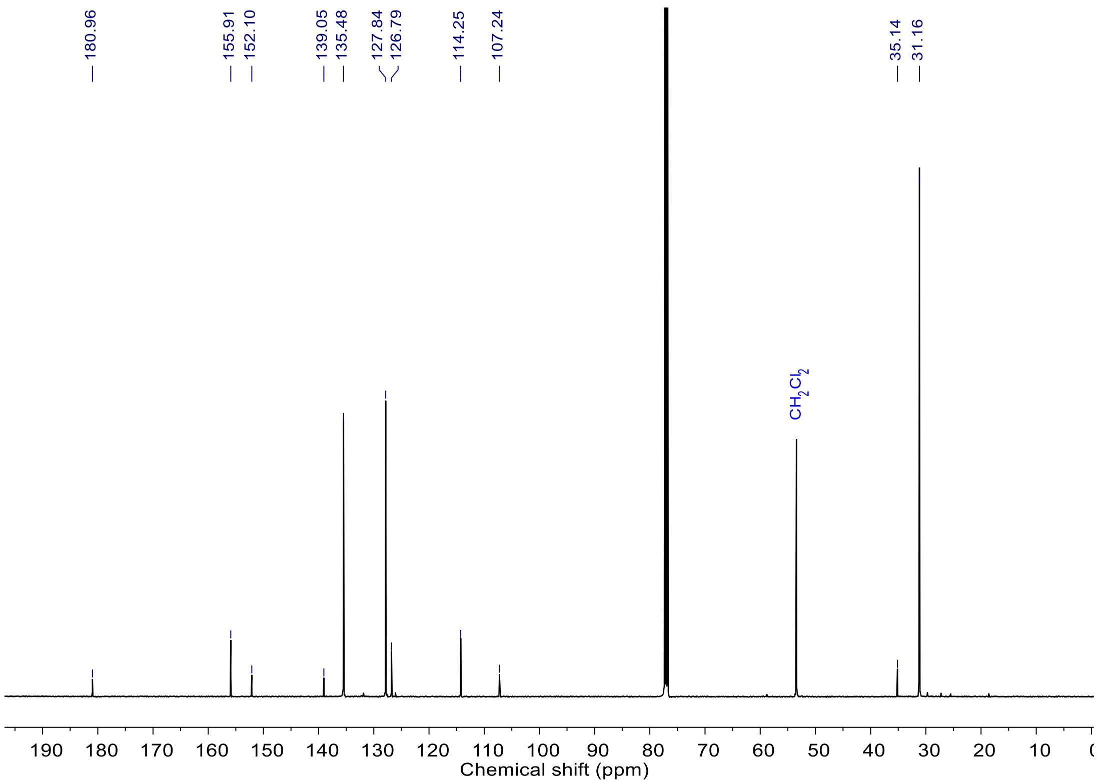

Fig. S48 ^{13}C NMR spectrum of **1** in $CDCl_3$.

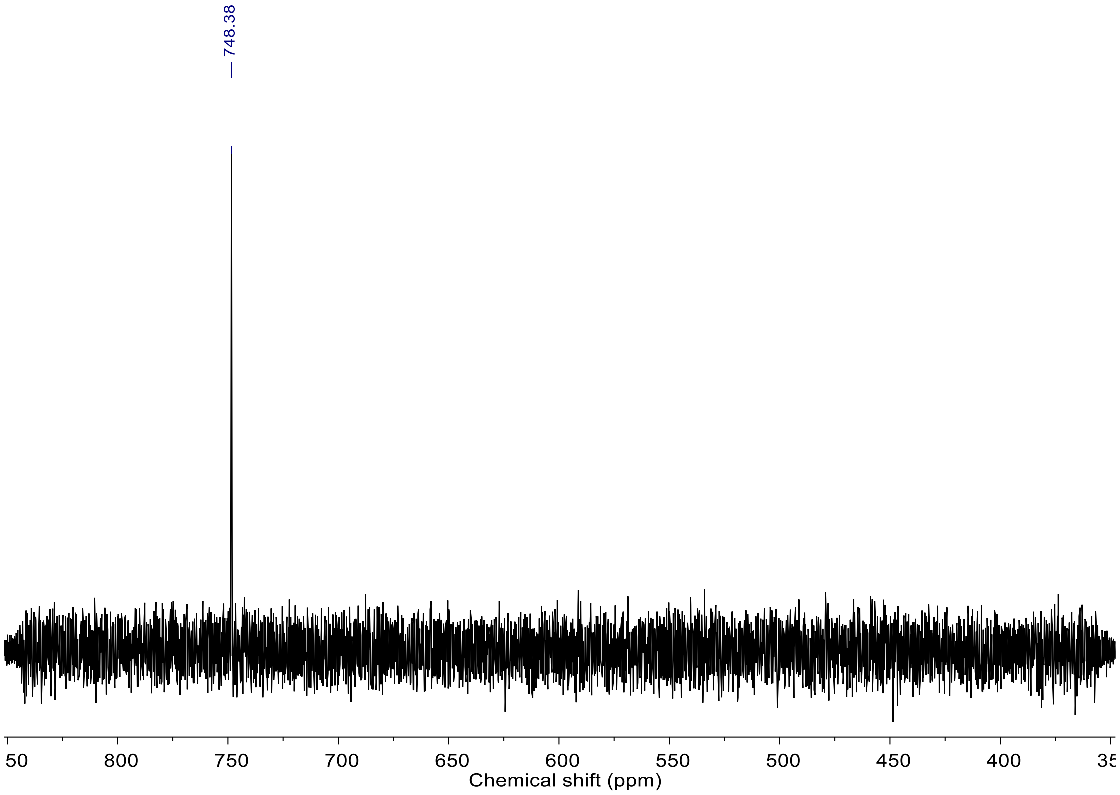

Fig. S49 ^{77}Se NMR spectrum of **1** in $CDCl_3$.

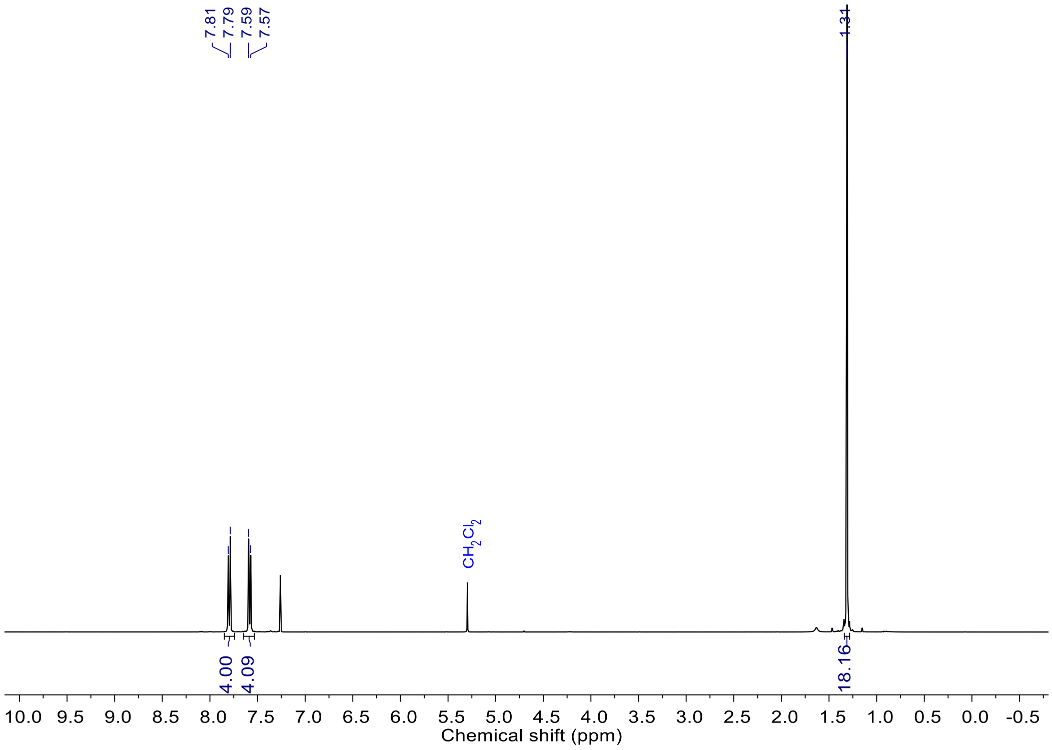

Fig. S50 ^{1}H NMR spectrum of **2 Chiral** in $CDCl_3$.

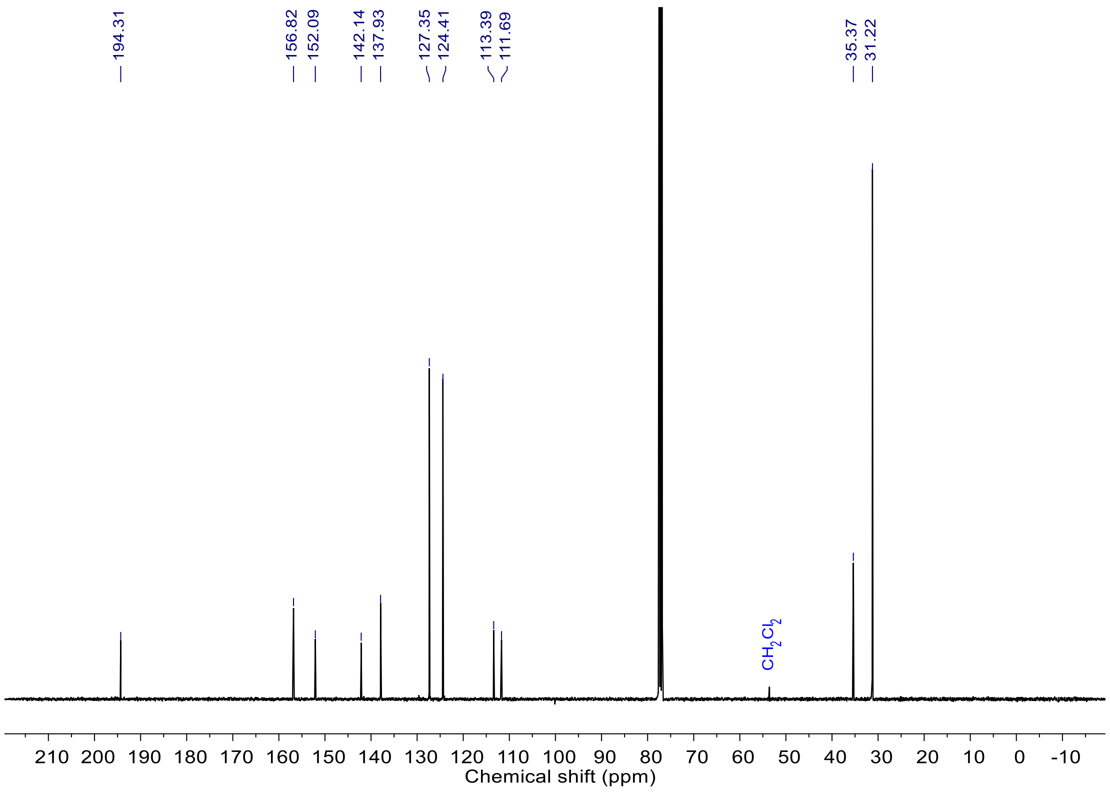

Fig. S51 ^{13}C NMR spectrum of **2 Chiral** in $CDCl_3$.

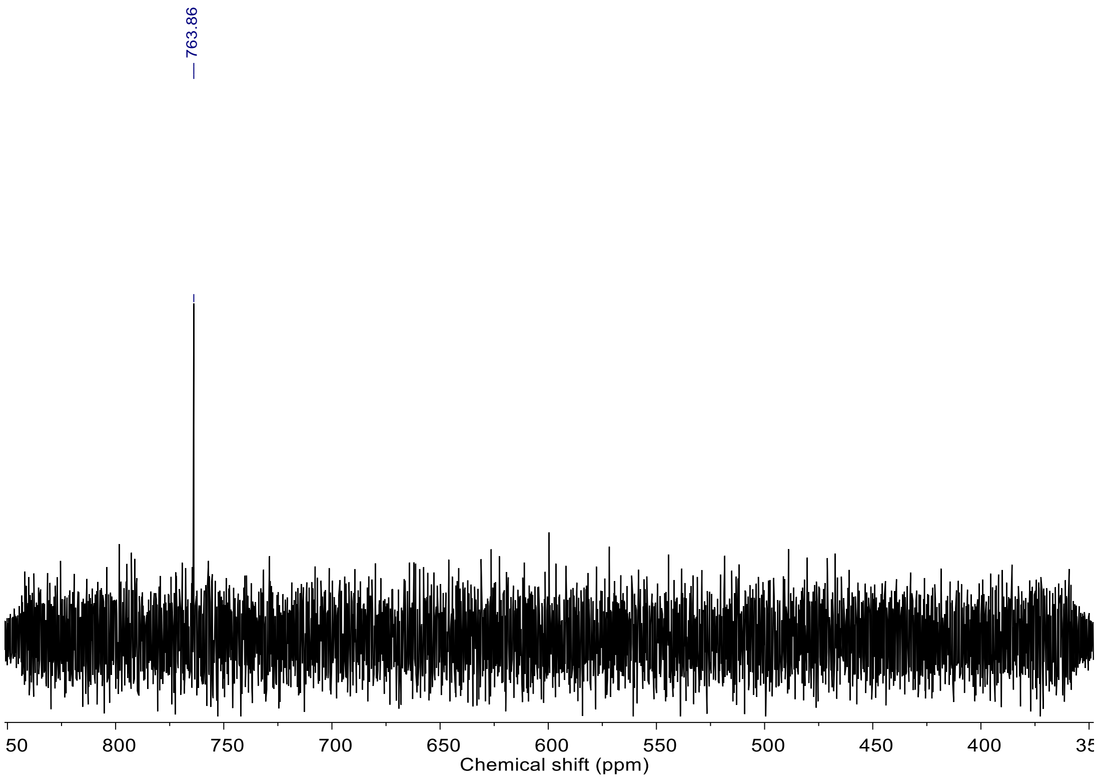

Fig. S52 ^{77}Se NMR spectrum of **2 Chiral** in $CDCl_3$.

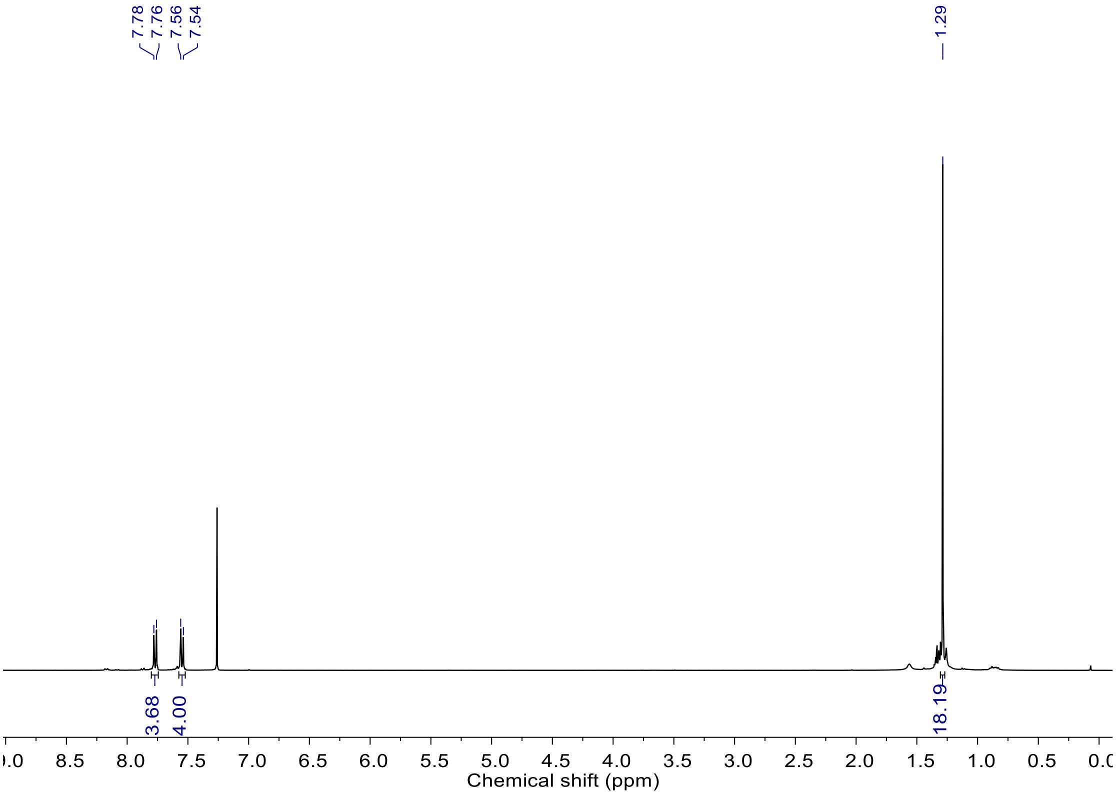

Fig. S53 ^{1}H NMR spectrum of **2 Meso** in $CDCl_3$.

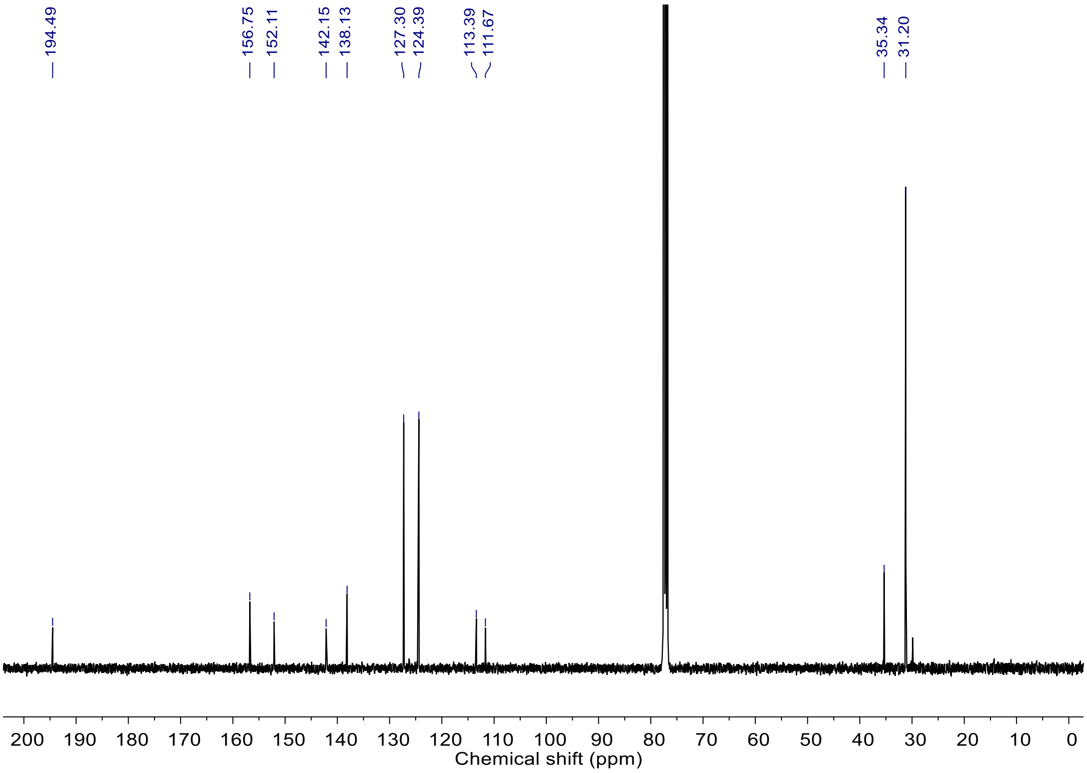

Fig. S54 ^{13}C NMR spectrum of **2 Meso** in $CDCl_3$.

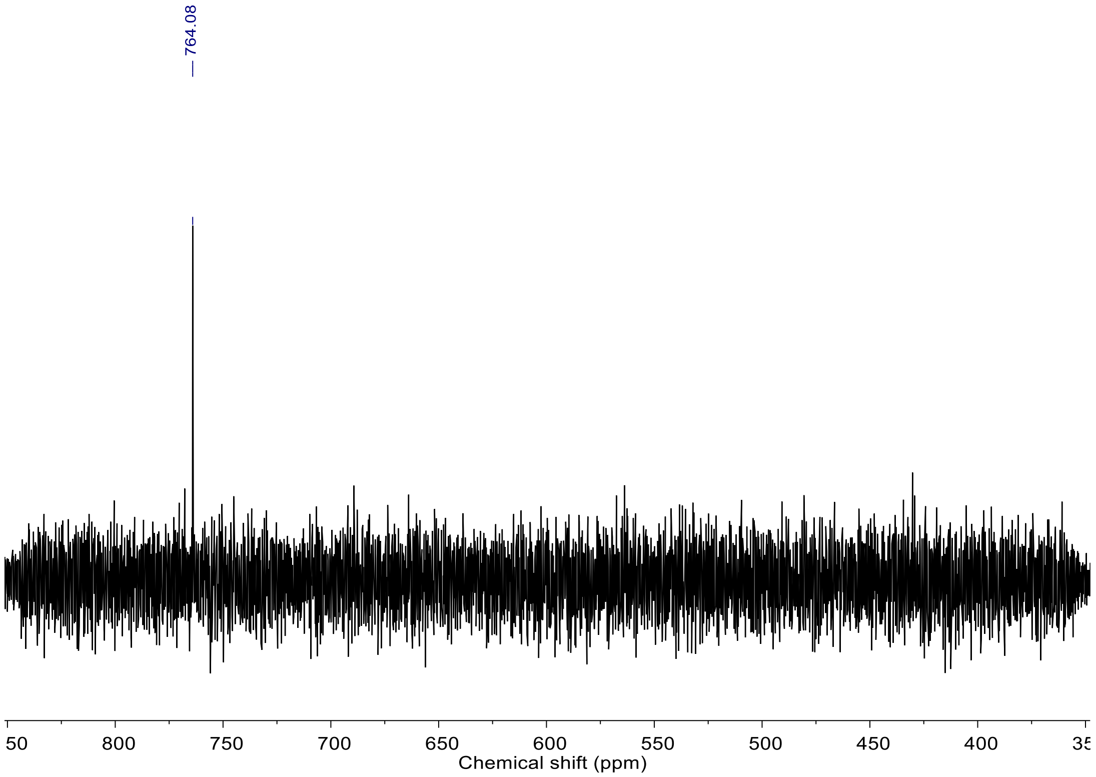

Fig. S55 ^{77}Se NMR spectrum of **2 Meso** in $CDCl_3$.

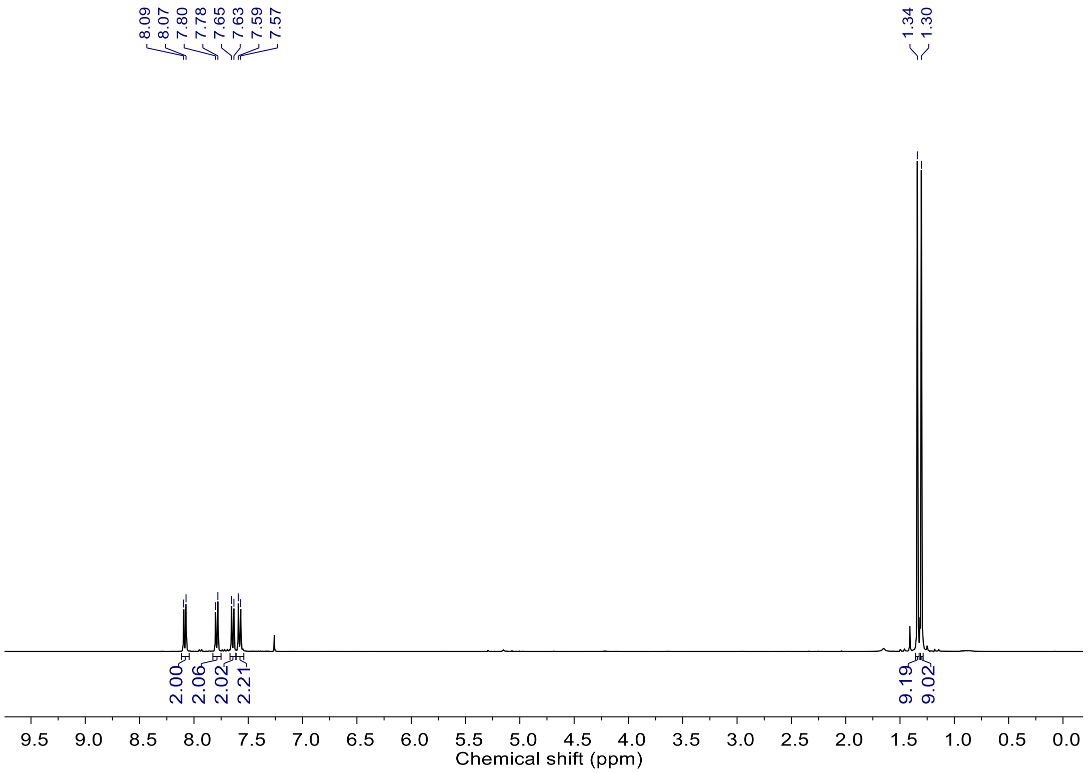

Fig. S56 ^{1}H NMR spectrum of **3** in $CDCl_3$.

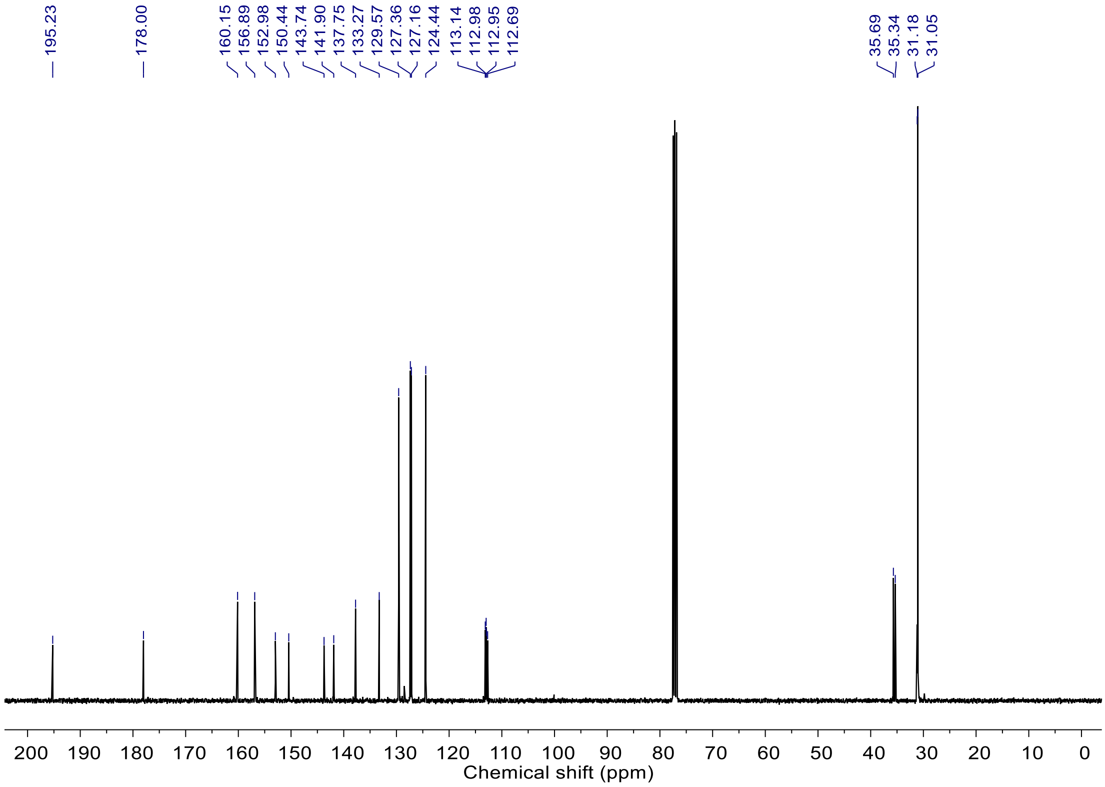

Fig. S57 ^{13}C NMR spectrum of **3** in $CDCl_3$.

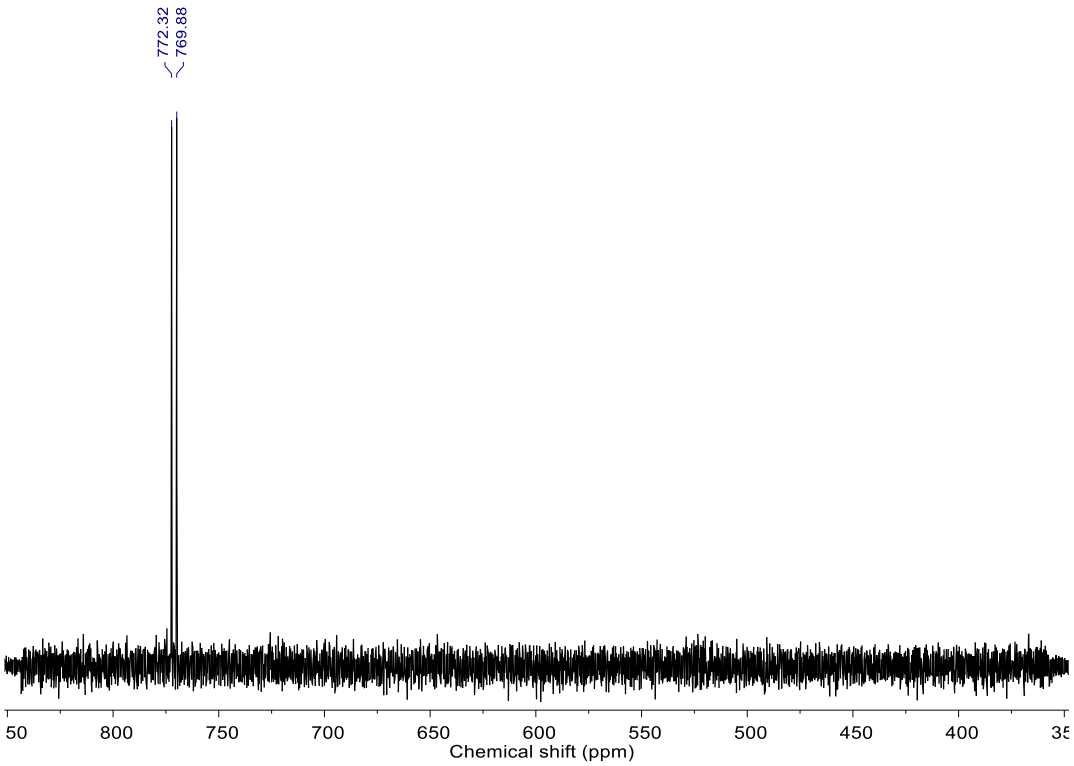

Fig. S58 ^{77}Se NMR spectrum of **3** in $CDCl_3$.

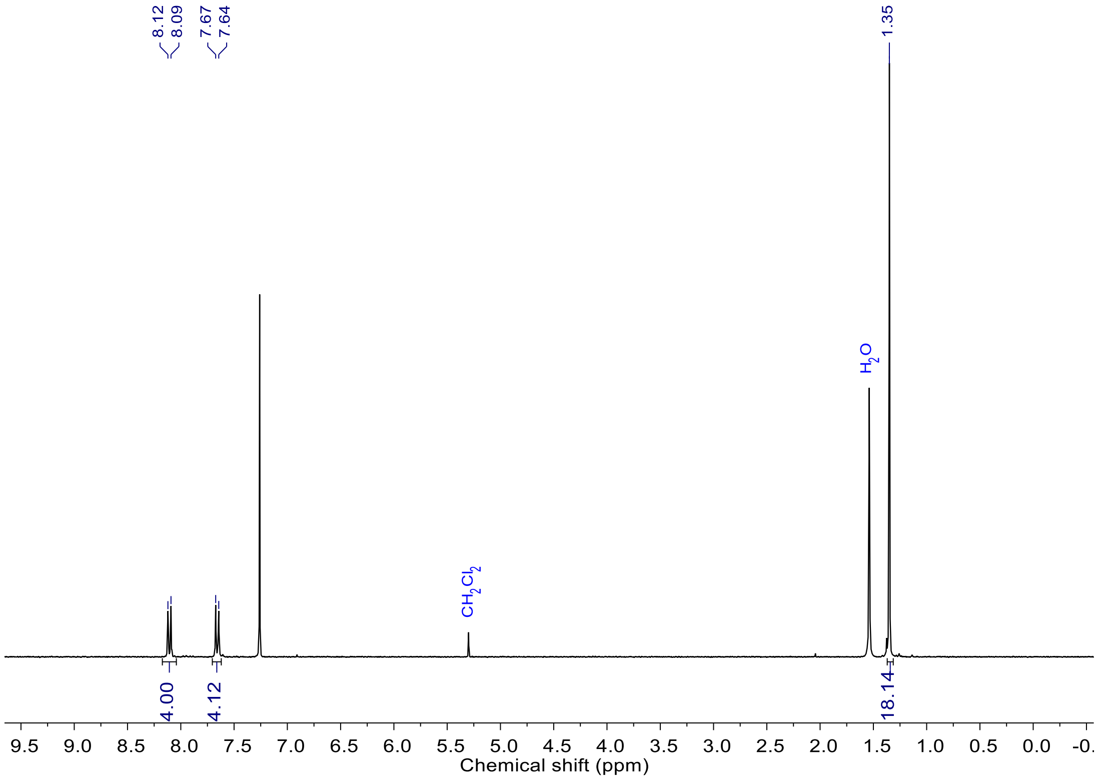

Fig. S59 ^{1}H NMR spectrum of **4** in $CDCl_3$.

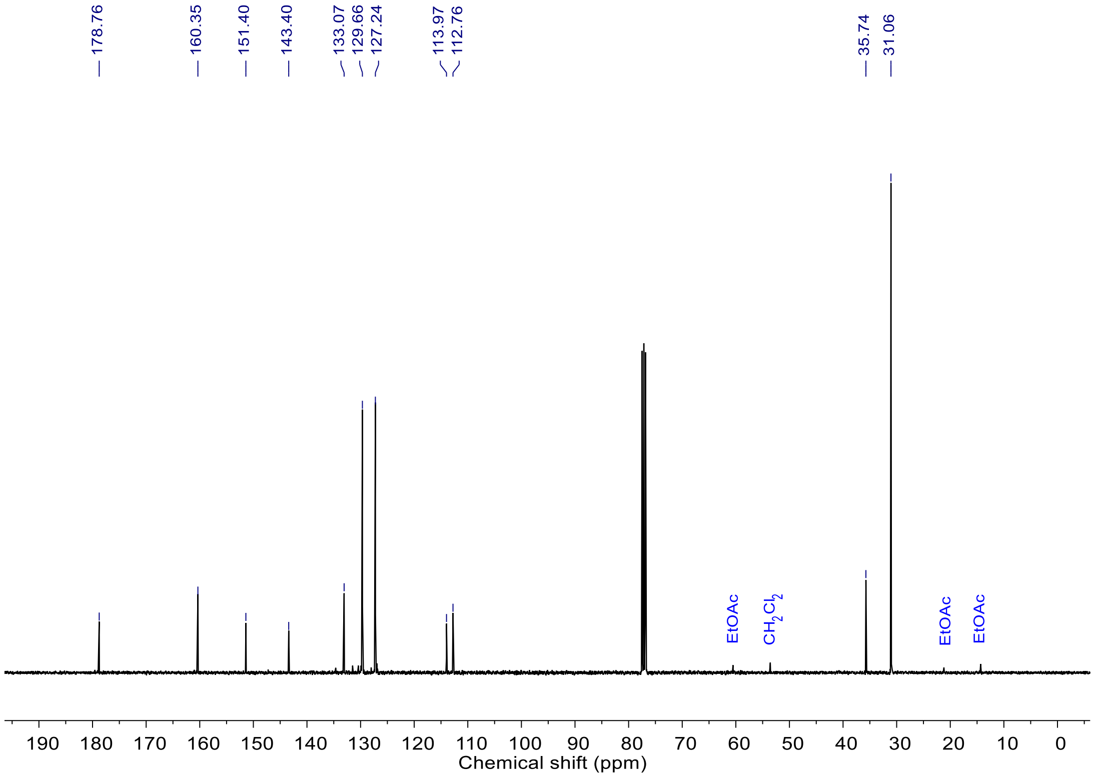

Fig. S60 ^{13}C NMR spectrum of **4** in $CDCl_3$.

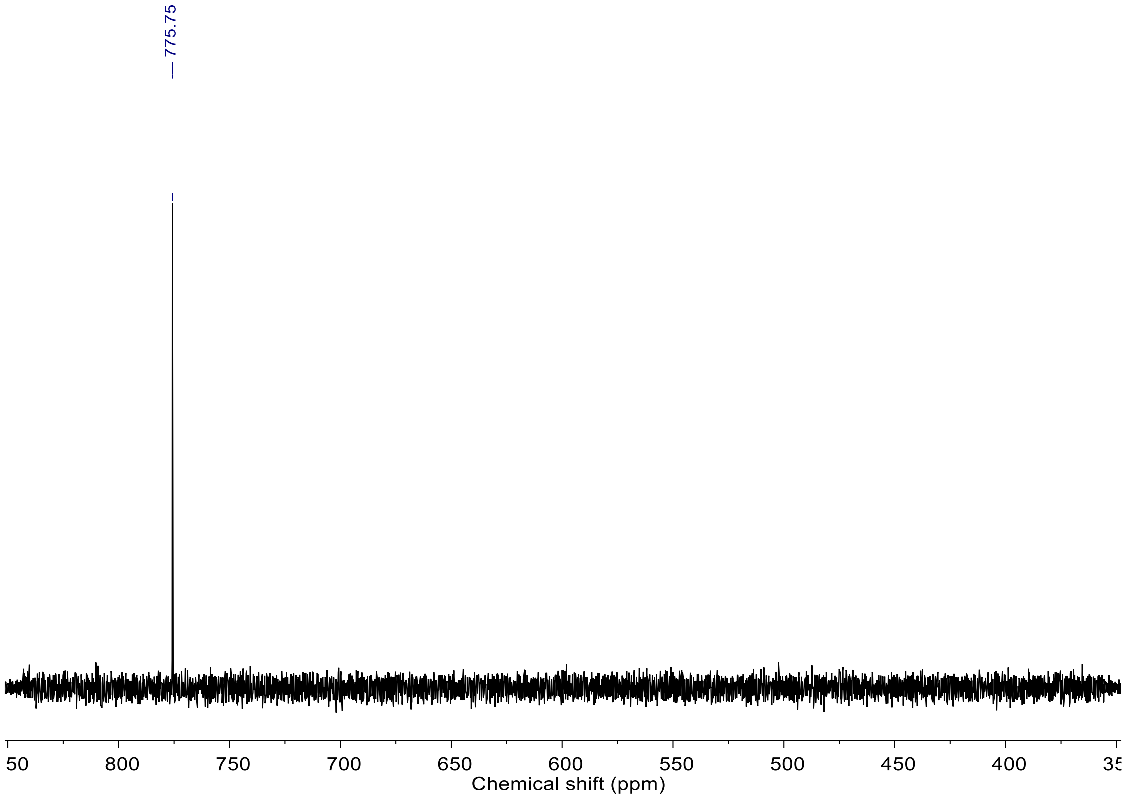

Fig. S61 ^{77}Se NMR spectrum of **4** in $CDCl_3$.

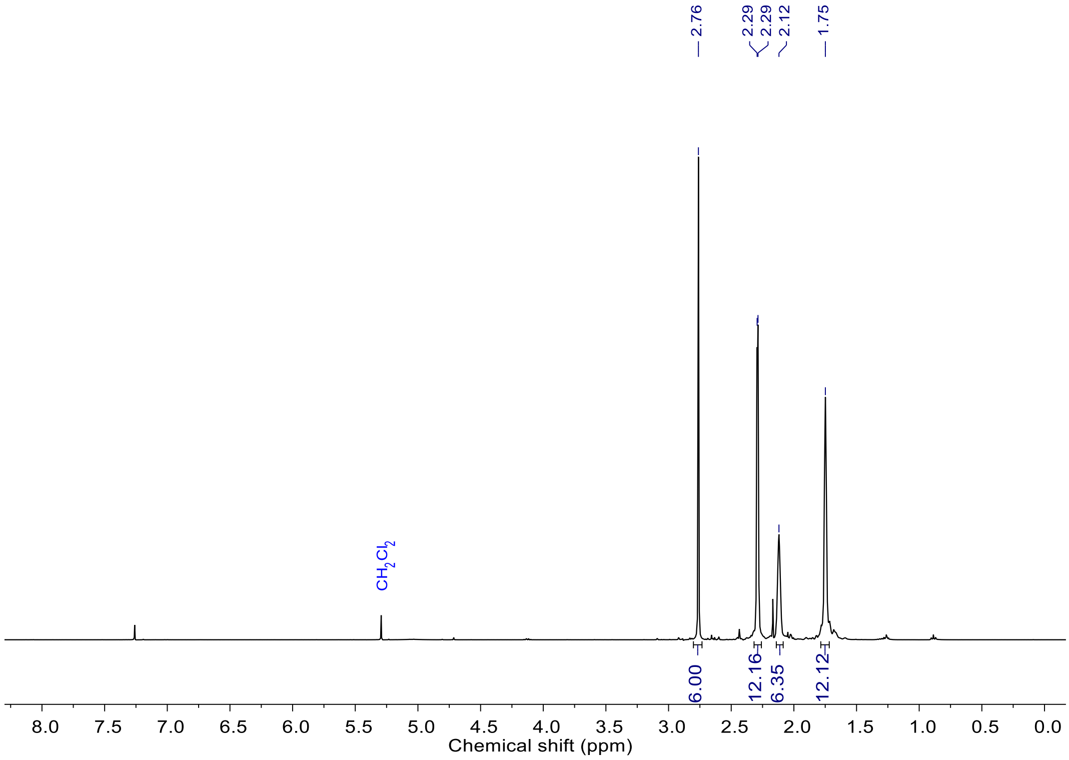

Fig. S62 ^{1}H NMR spectrum of **7** in $CDCl_3$.

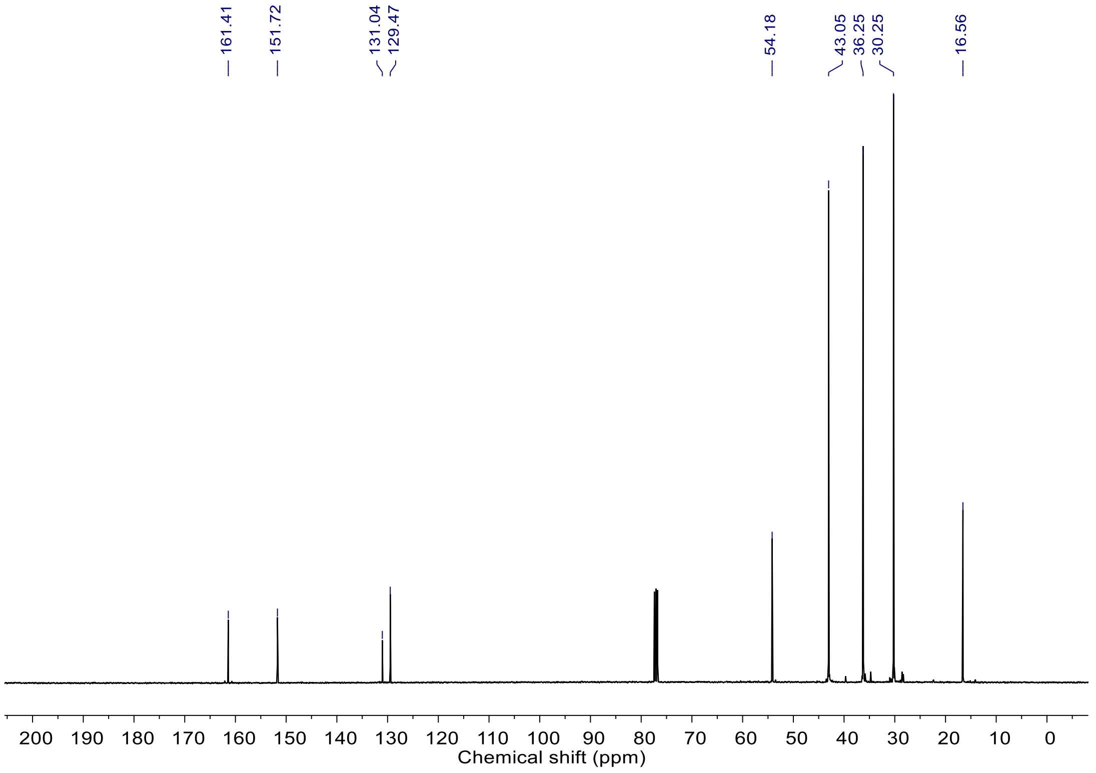

Fig. S63 ^{13}C NMR spectrum of **7** in $CDCl_3$.

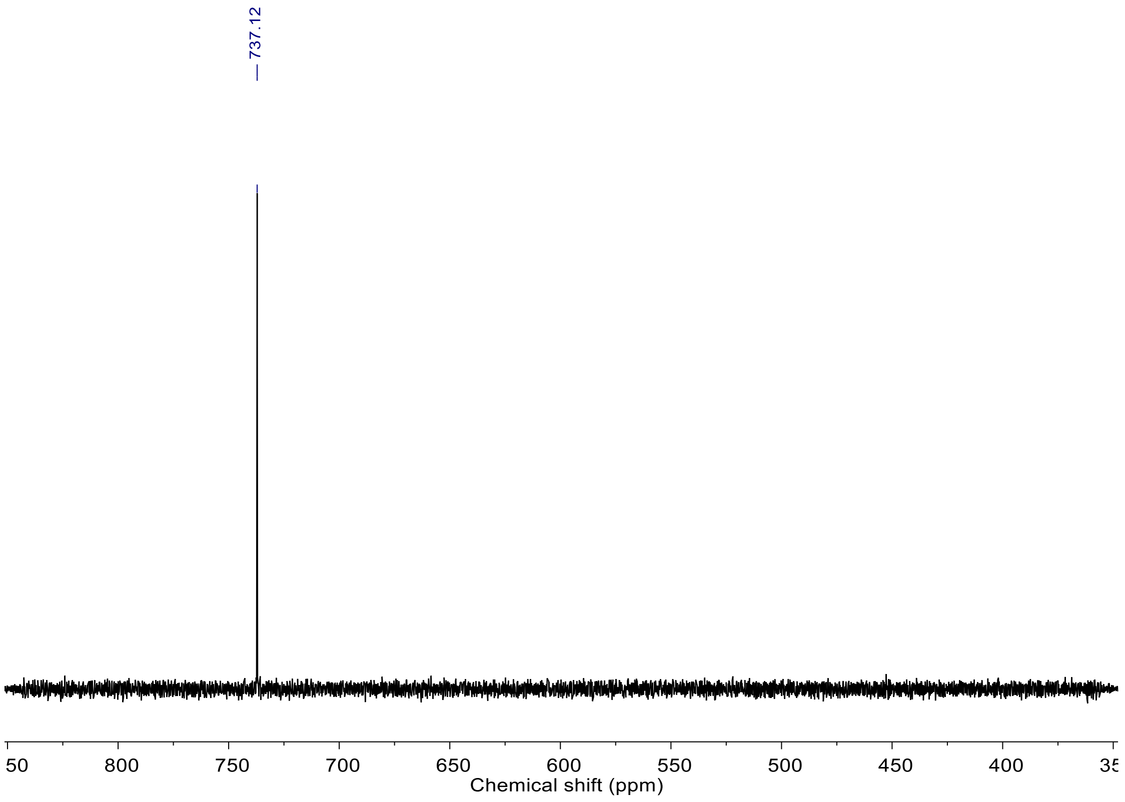

Fig. S64 ^{77}Se NMR spectrum of **7** in $CDCl_3$.

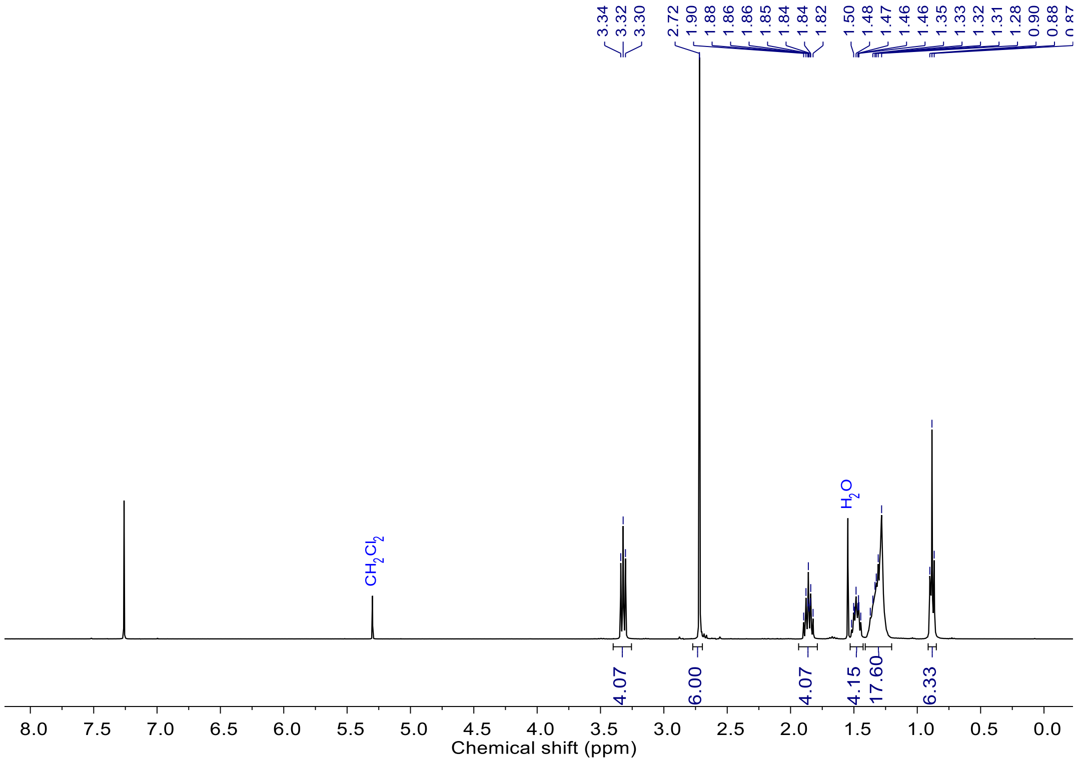

Fig. S65 ^{1}H NMR spectrum of **10** in $CDCl_3$.

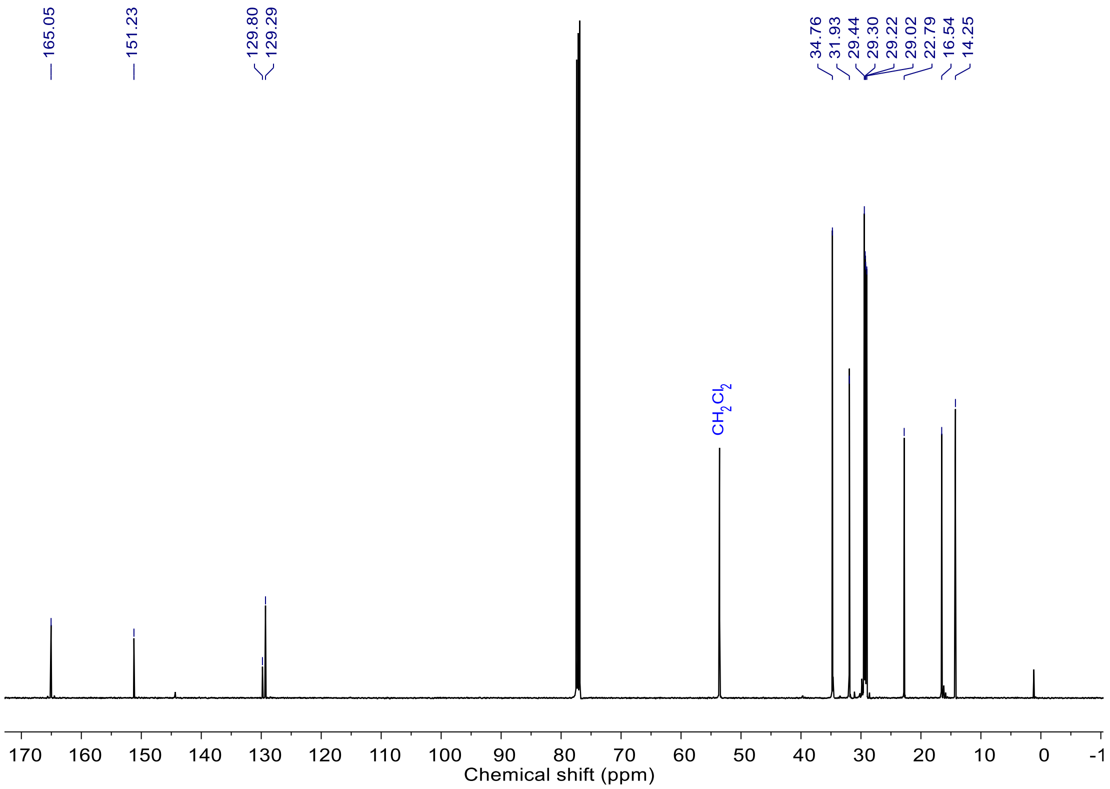

Fig. S66 ^{13}C NMR spectrum of **10** in $CDCl_3$.

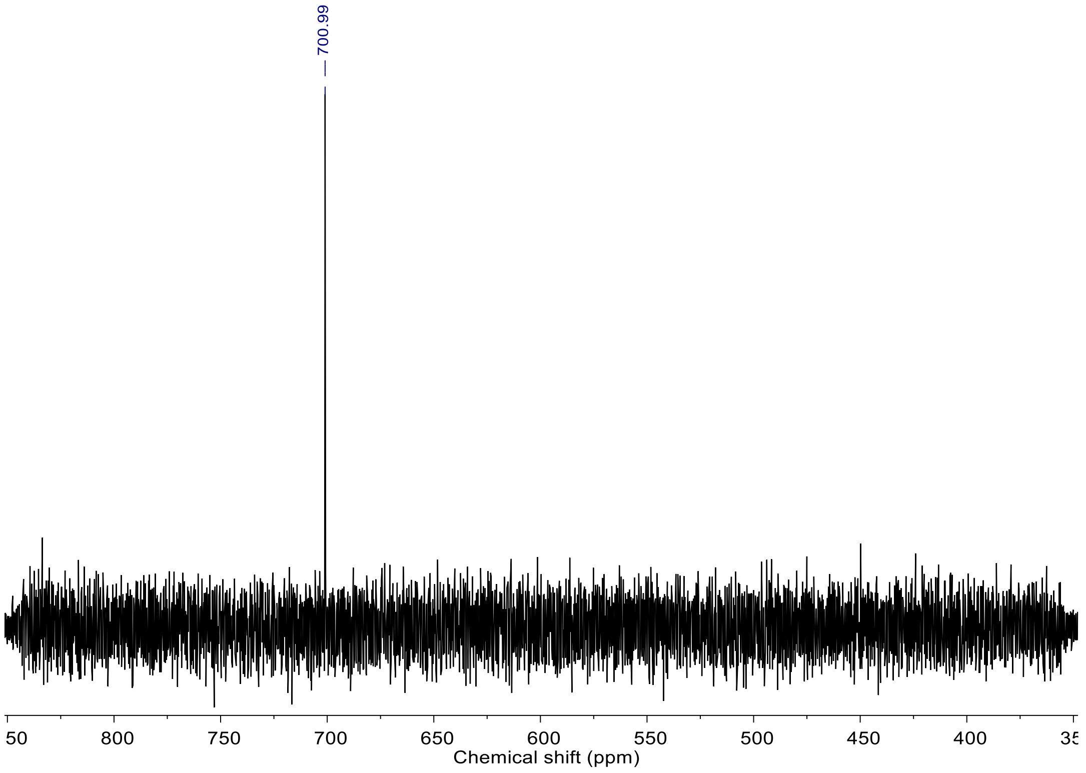

Fig. S67 ^{77}Se NMR spectrum of **10** in $CDCl_3$.

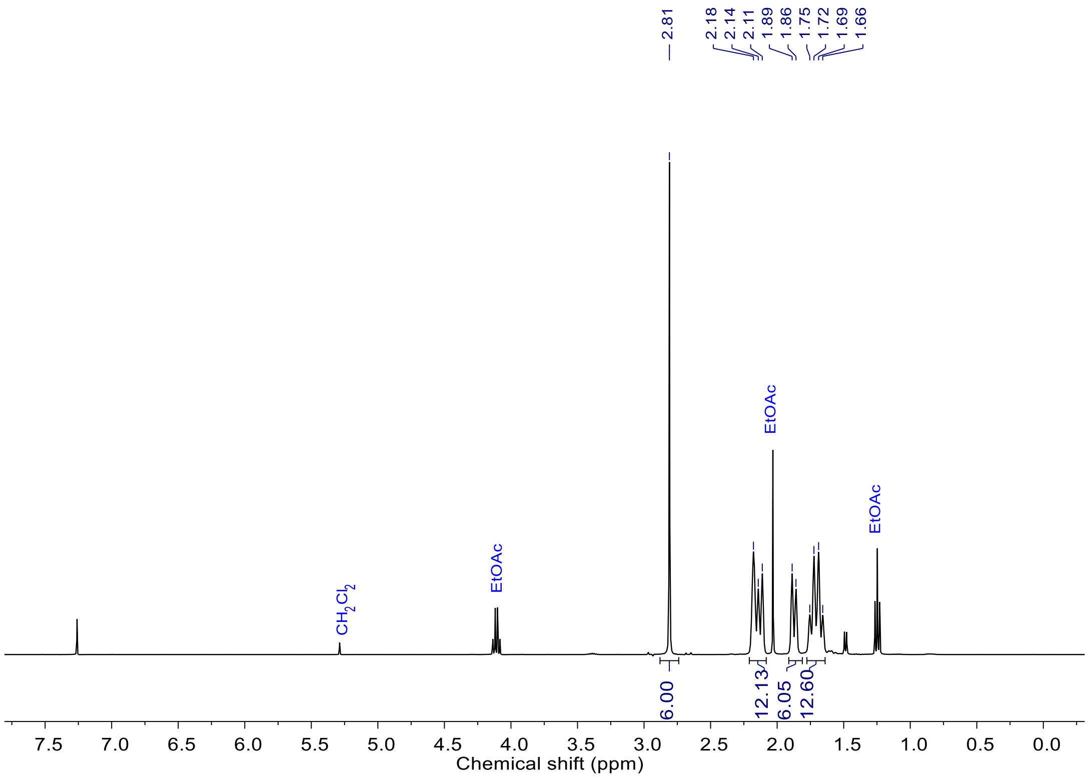

Fig. S68 ^{1}H NMR spectrum of **8 Chiral** in $CDCl_3$.

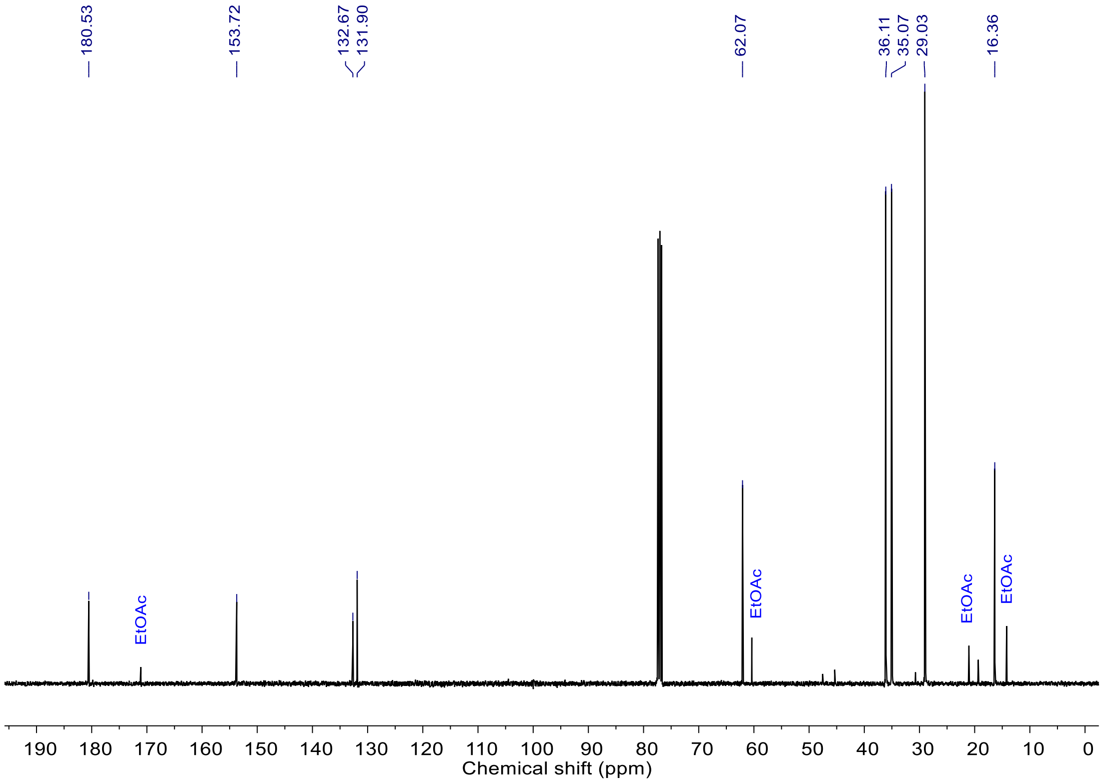

Fig. S69 ^{13}C NMR spectrum of **8 Chiral** in $CDCl_3$.

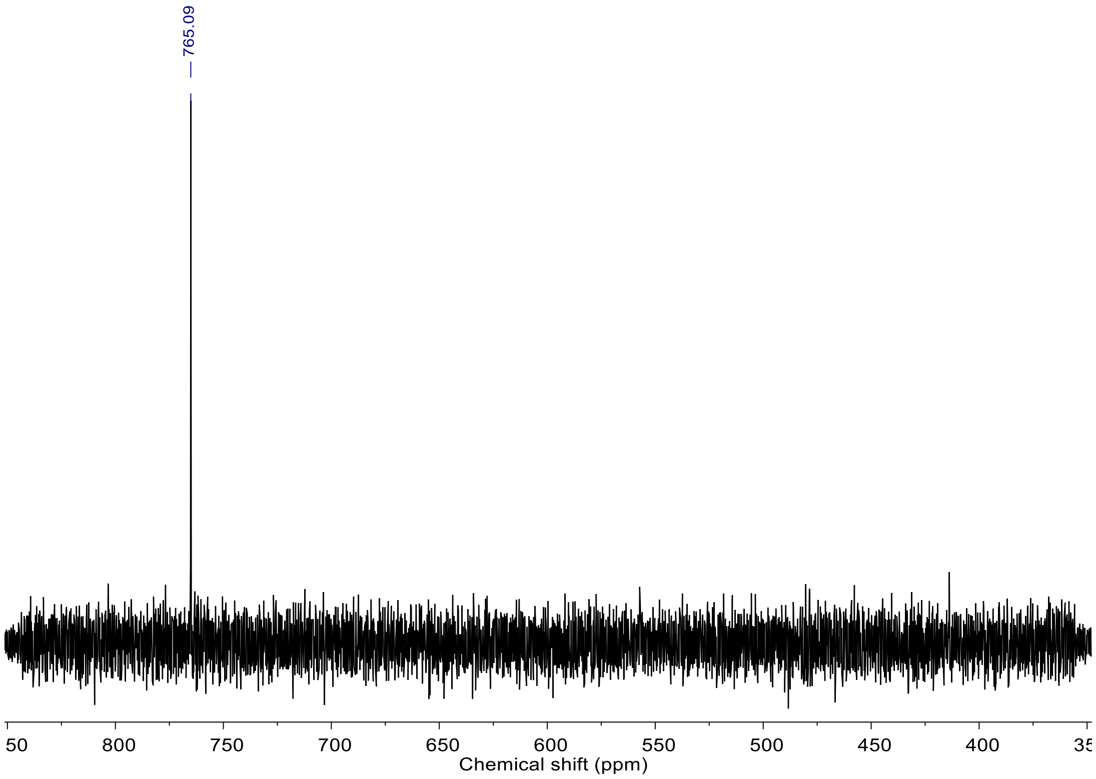

Fig. S70 ^{77}Se NMR spectrum of **8 Chiral** in $CDCl_3$.

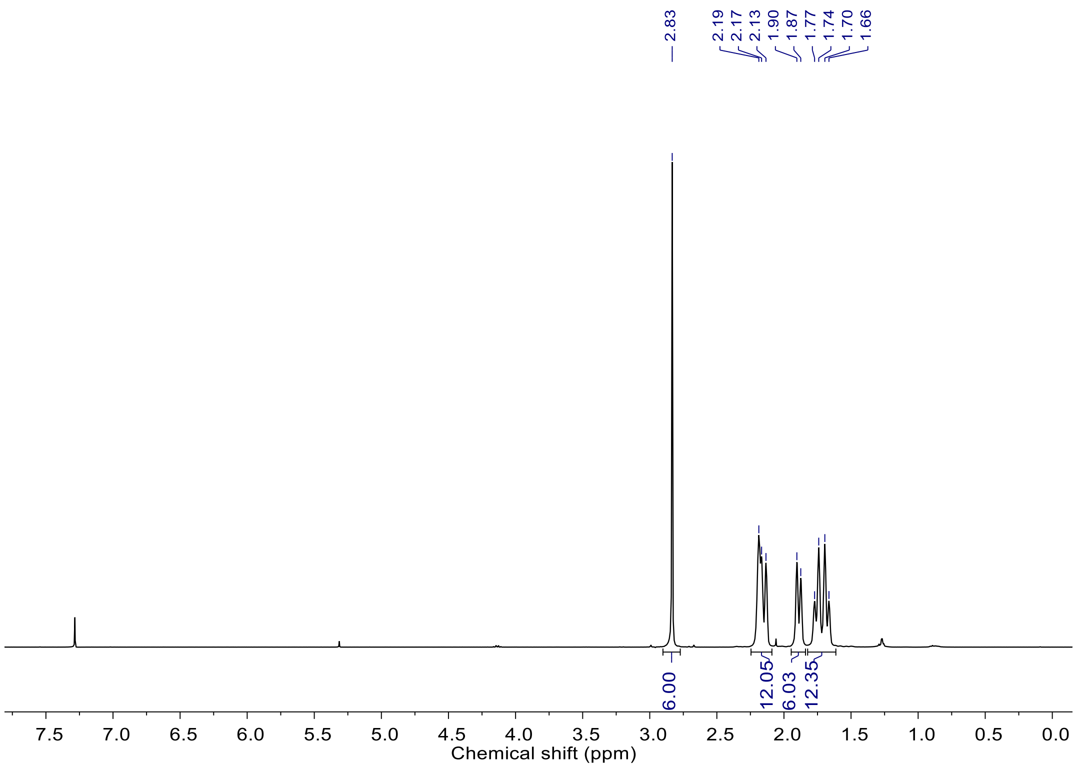

Fig. S71 ^{1}H NMR spectrum of **8 Meso** in $CDCl_3$.

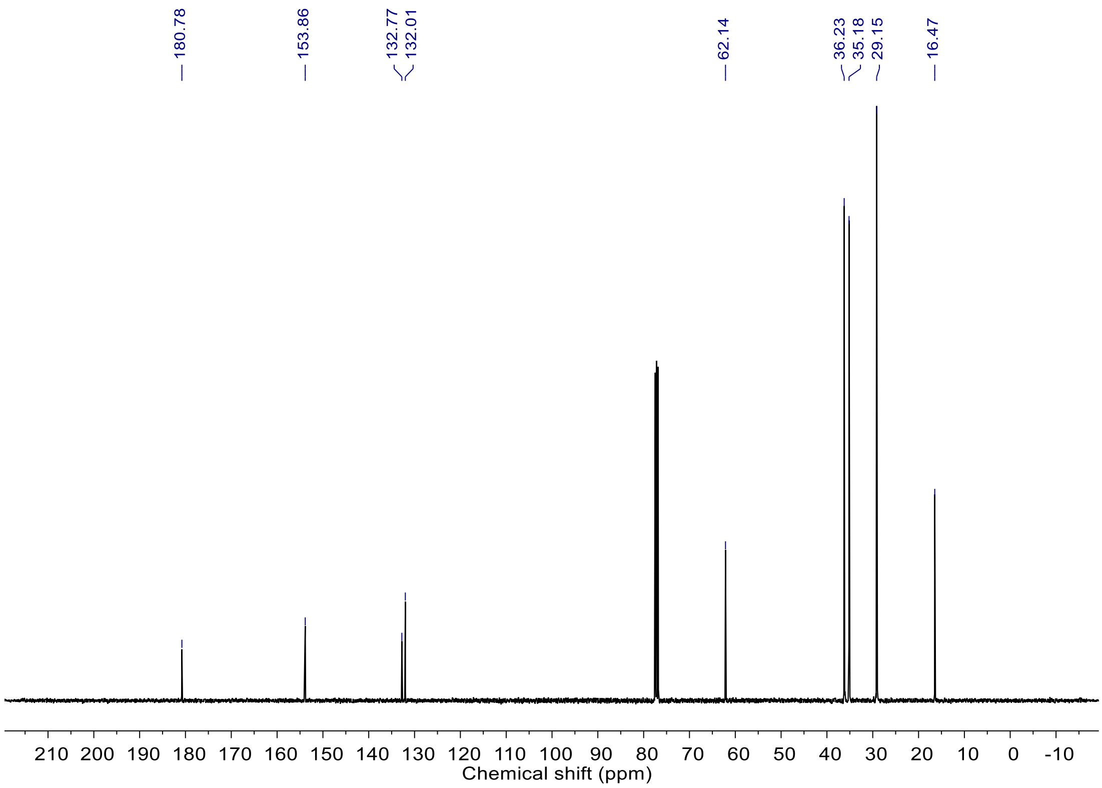

Fig. S72 ^{13}C NMR spectrum of **8 Meso** in $CDCl_3$.

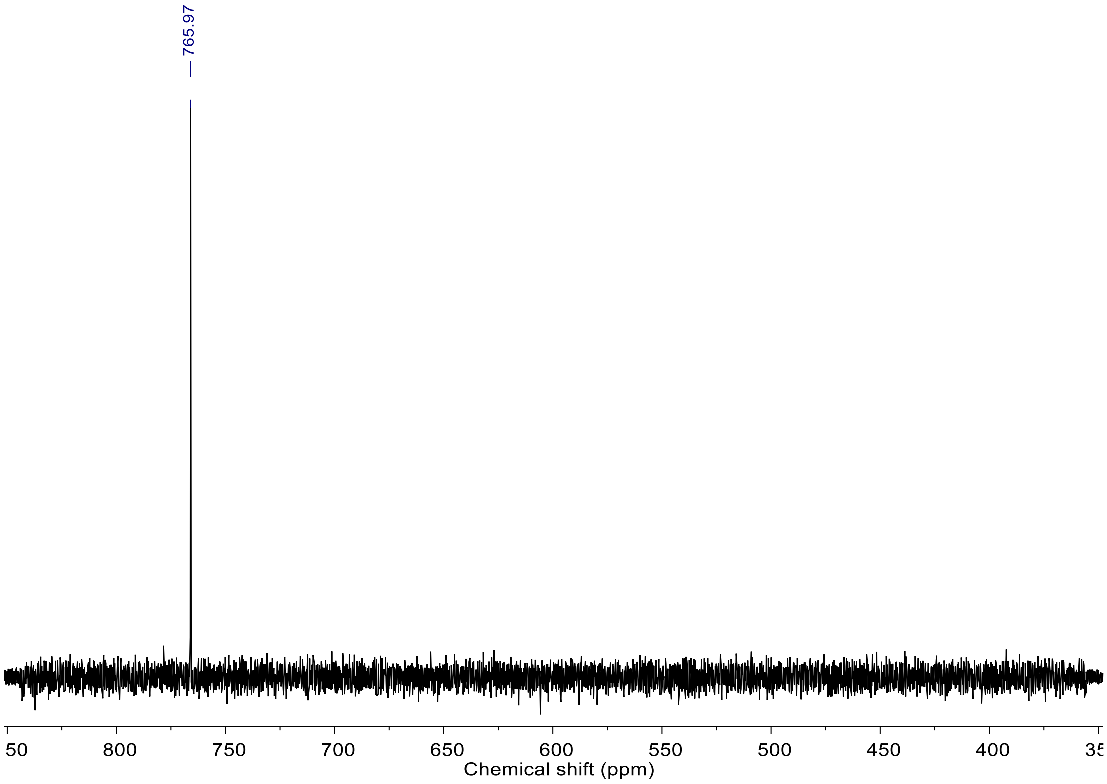

Fig. S73 ^{77}Se NMR spectrum of **8 Meso** in $CDCl_3$.

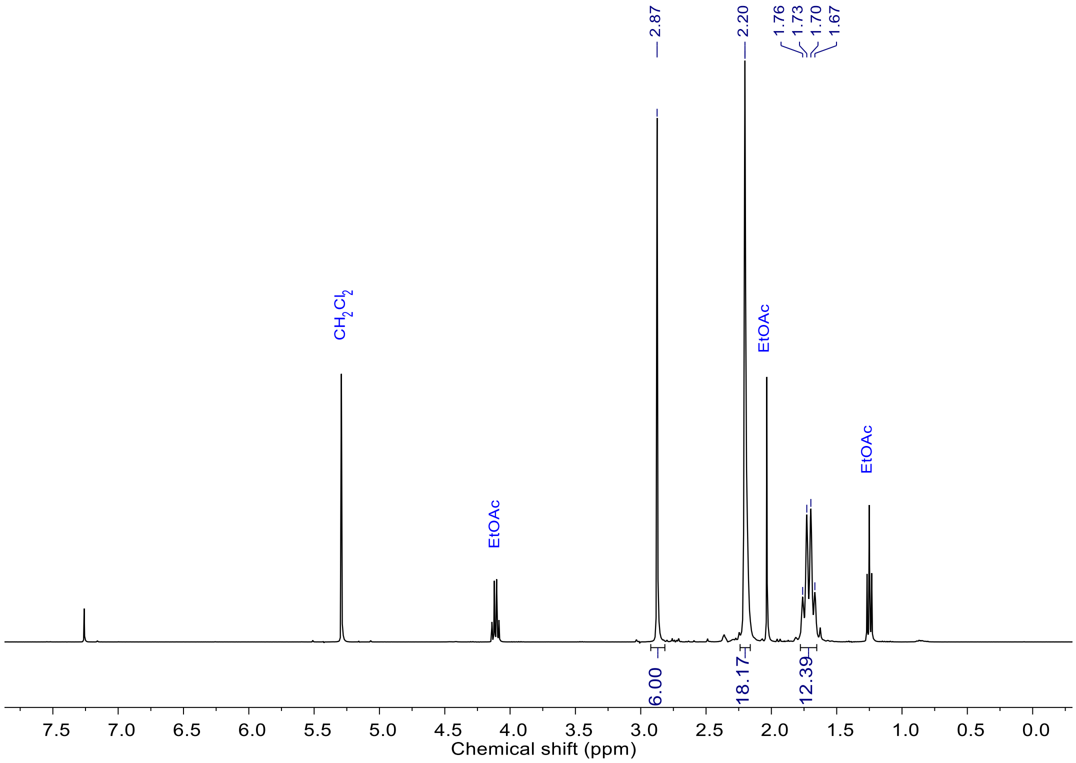

Fig. S74 ^{1}H NMR spectrum of **9** in $CDCl_3$.

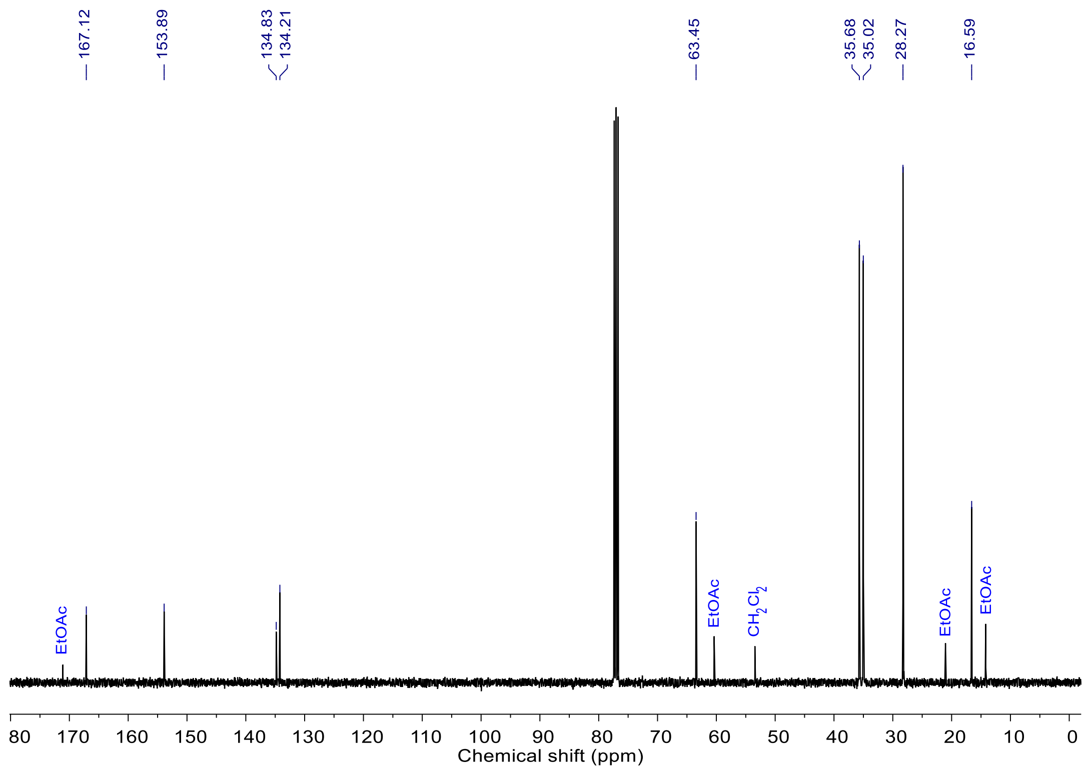

Fig. S75 ^{13}C NMR spectrum of **9** in $CDCl_3$.

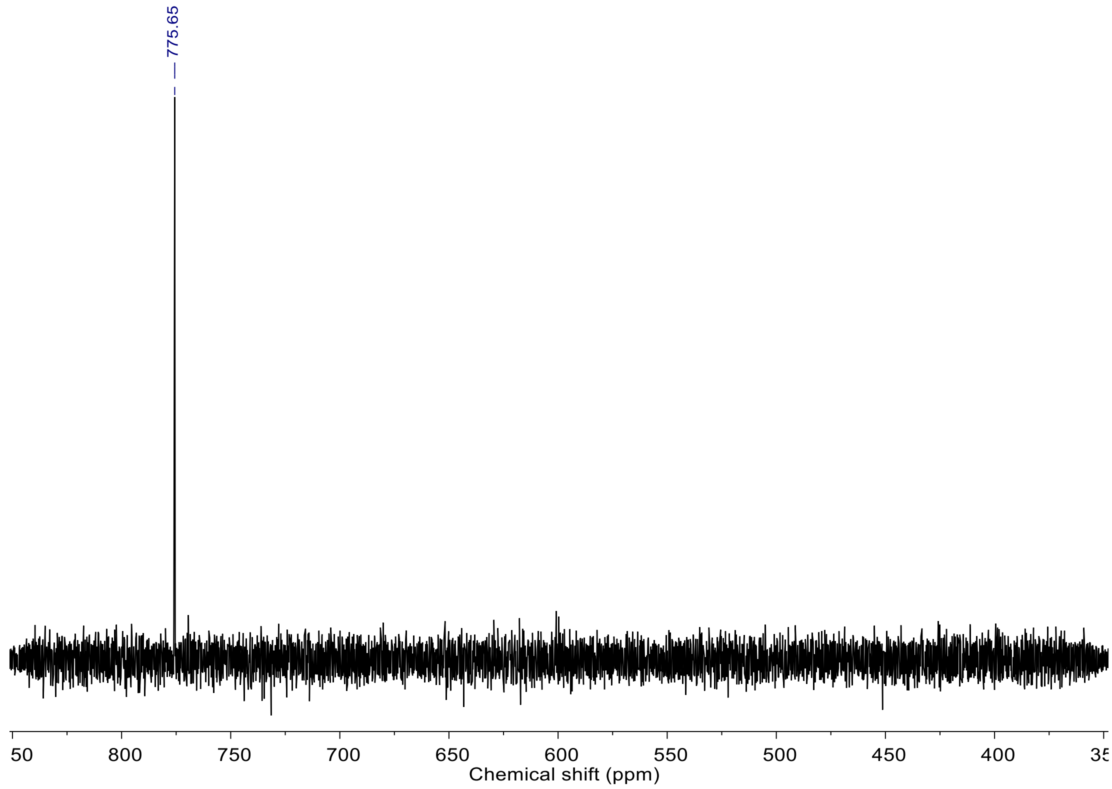

Fig. S76 ^{77}Se NMR spectrum of **9** in $CDCl_3$.

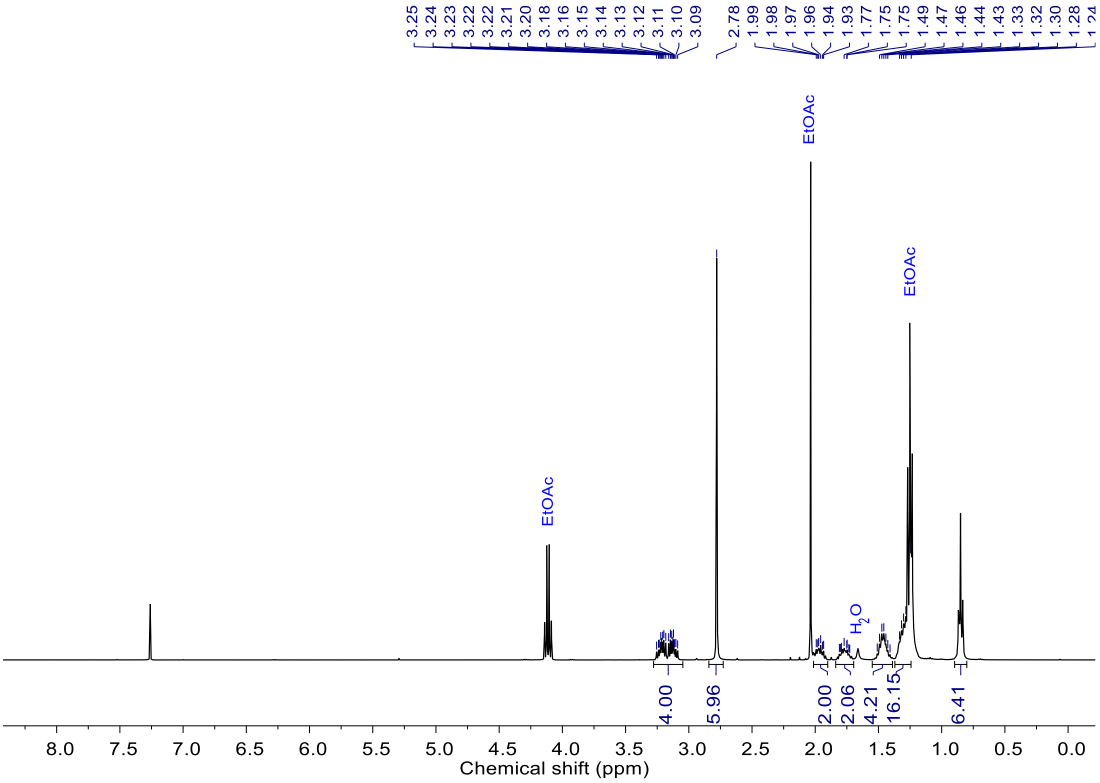

Fig. S77 ^{1}H NMR spectrum of **11 Chiral** in $CDCl_3$.

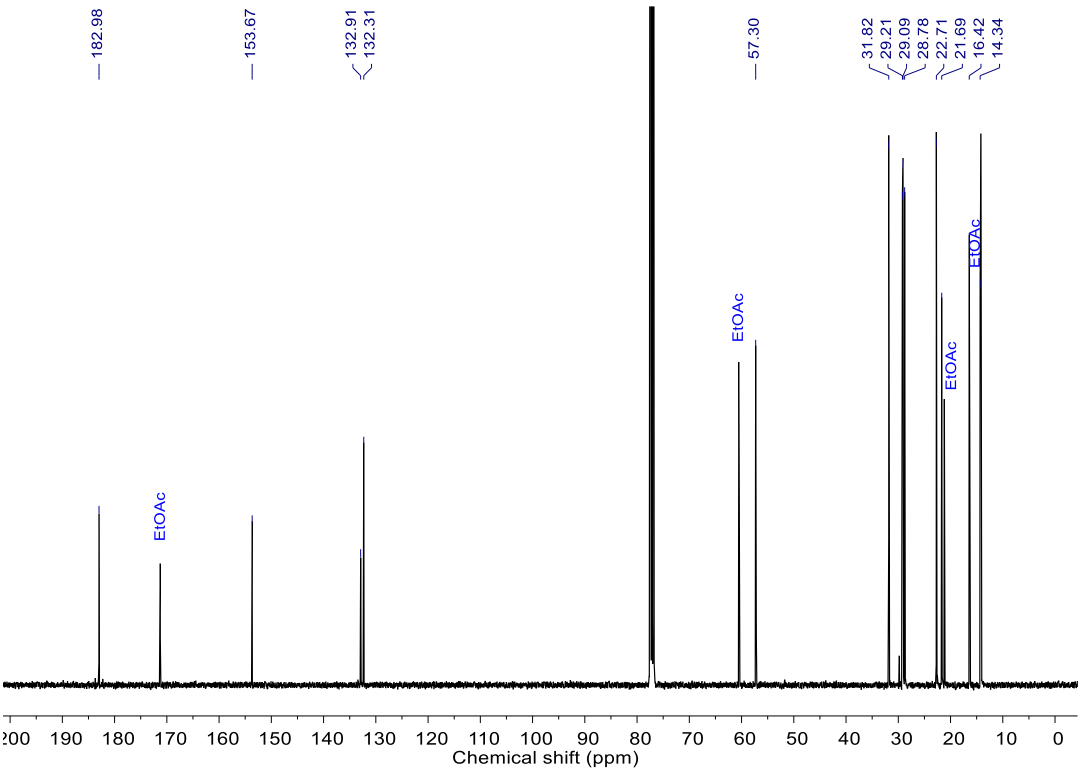

Fig. S78 ^{13}C NMR spectrum of **11 Chiral** in $CDCl_3$.

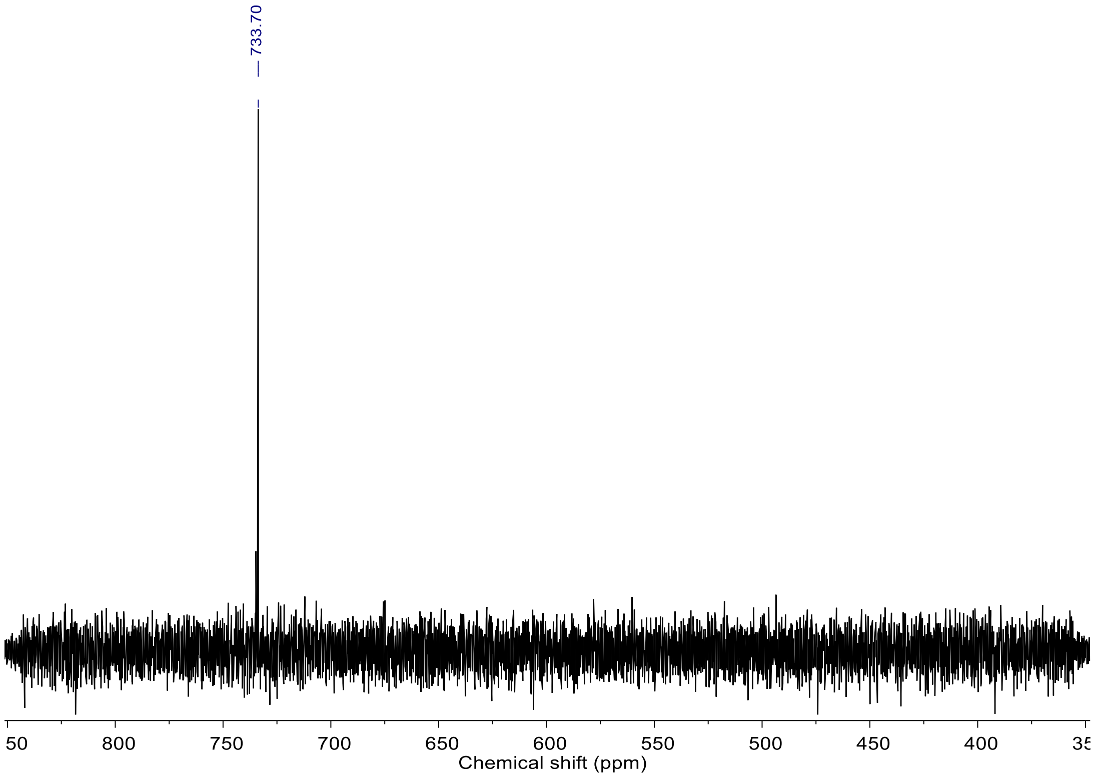

Fig. S79 ^{77}Se NMR spectrum of **11 Chiral** in $CDCl_3$.

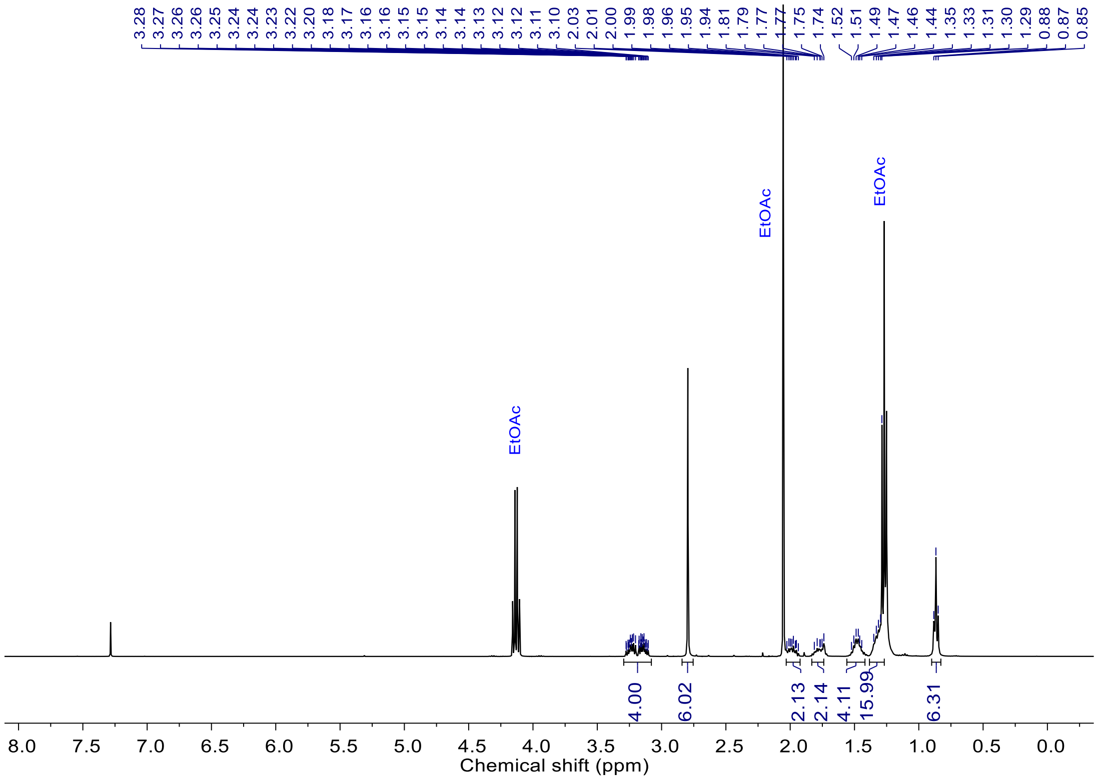

Fig. S80 ^{1}H NMR spectrum of **11 Meso** in $CDCl_3$.

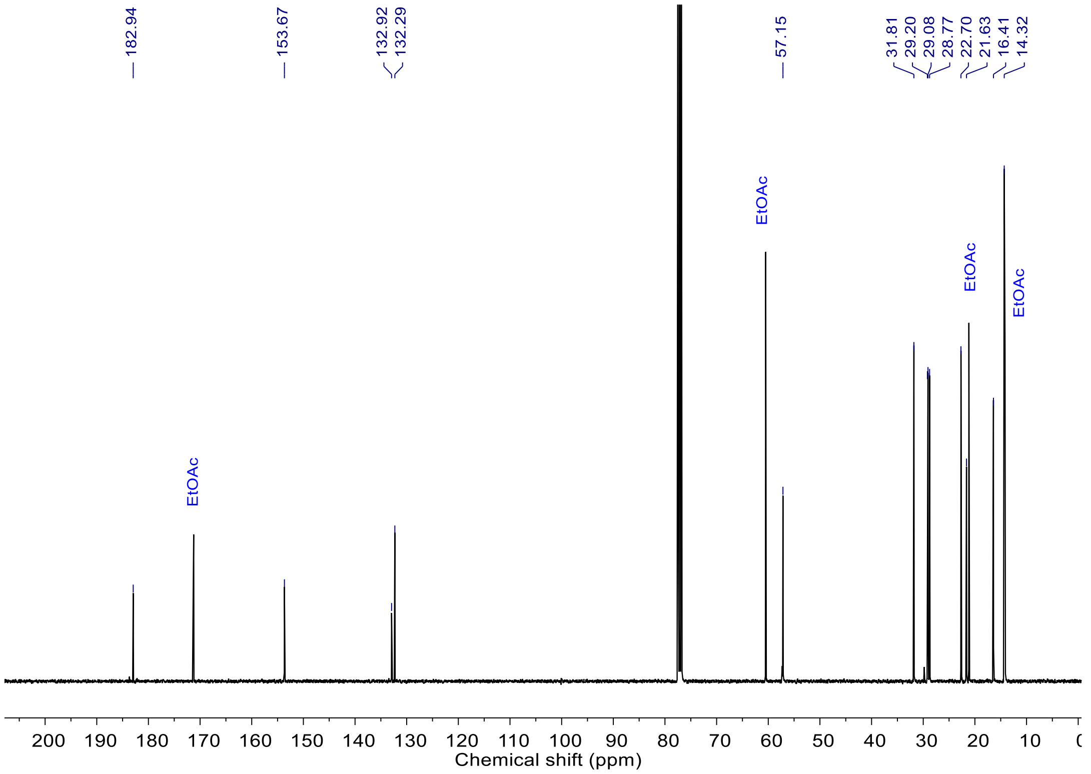

Fig. S81 ^{13}C NMR spectrum of **11 Meso** in $CDCl_3$.

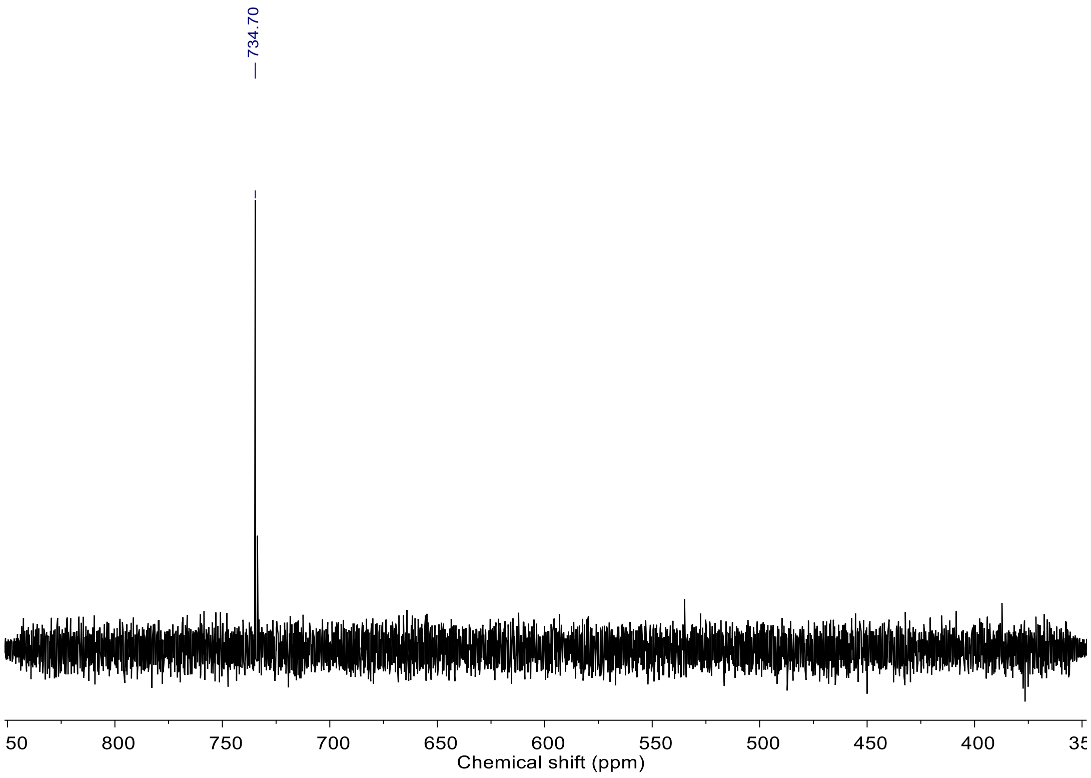

Fig. S82 ^{77}Se NMR spectrum of **11 Meso** in $CDCl_3$.

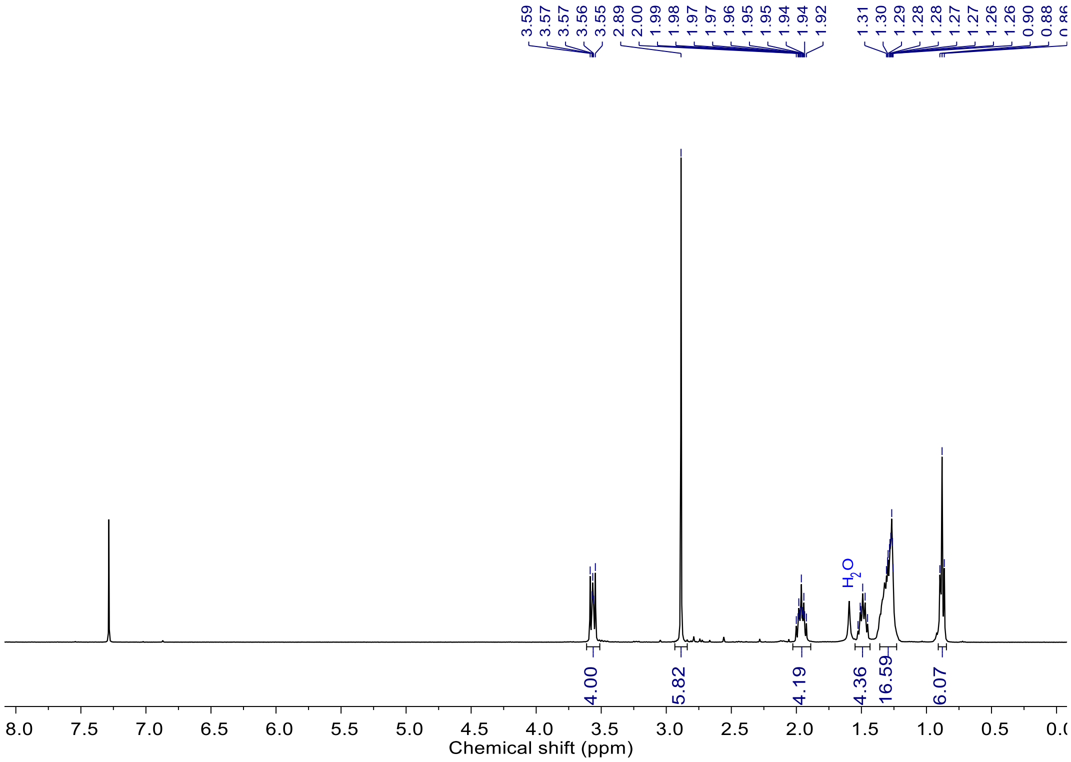

Fig. S83 ^{1}H NMR spectrum of **12** in $CDCl_3$

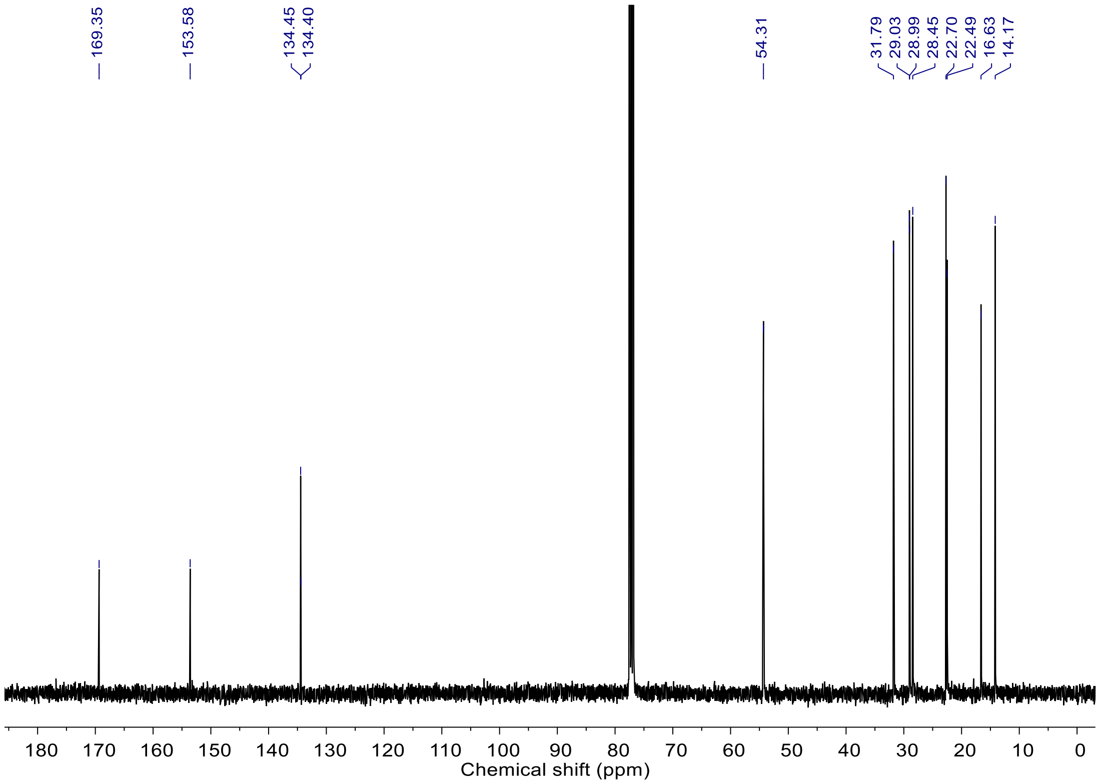

Fig. S84 ^{13}C NMR spectrum of **12** in $CDCl_3$.

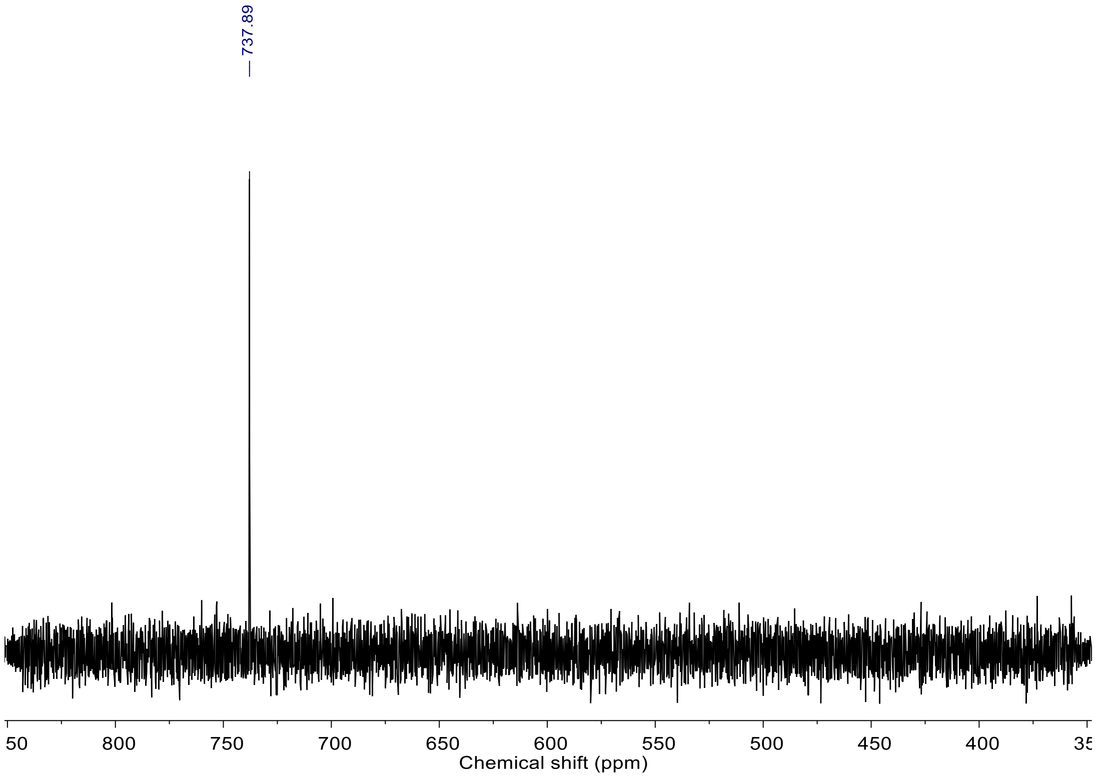

Fig. S85 ^{77}Se NMR spectrum of **12** in $CDCl_3$.

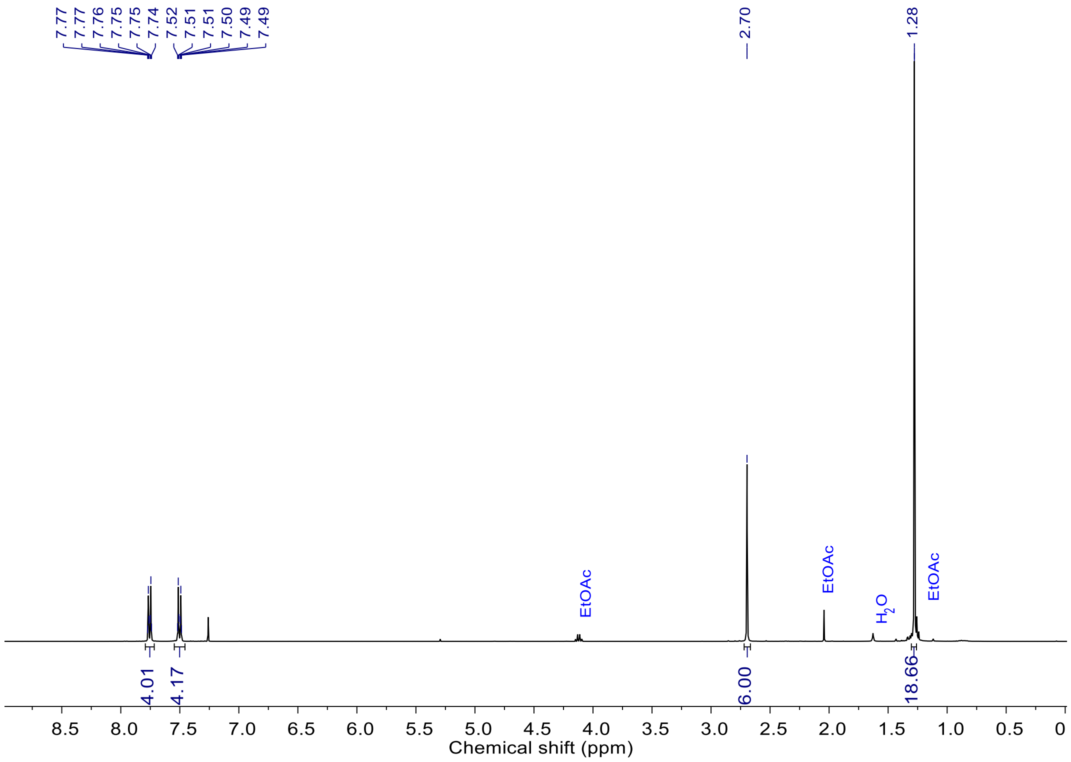

Fig. S86 ^{1}H NMR spectrum of **6 Meso** in $CDCl_3$.

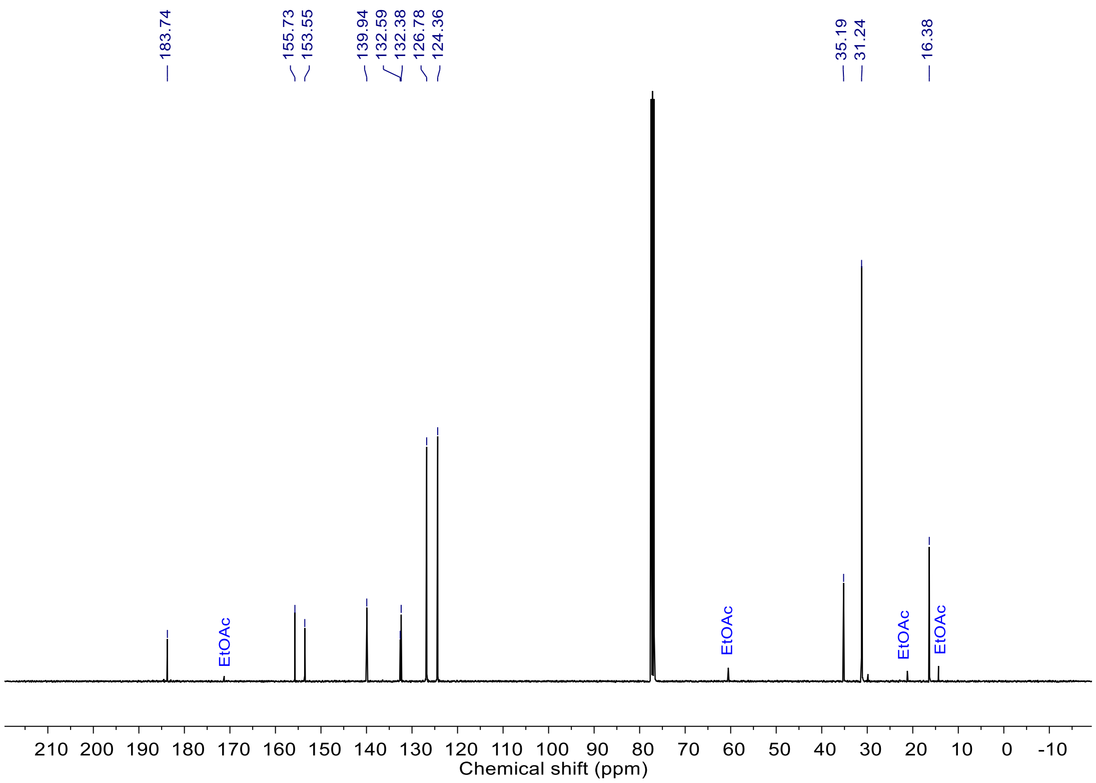

Fig. S87 ^{13}C NMR spectrum of **6 Meso** in $CDCl_3$.

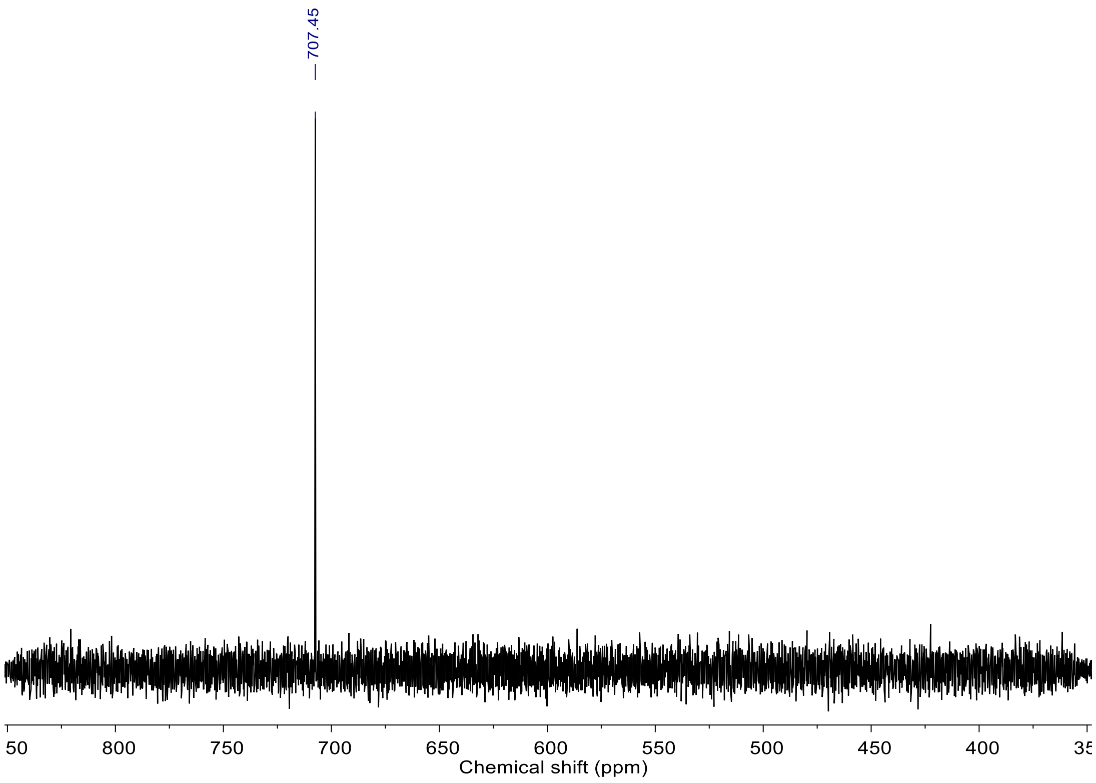

Fig. S88 ^{77}Se NMR spectrum of **6 Meso** in $CDCl_3$.

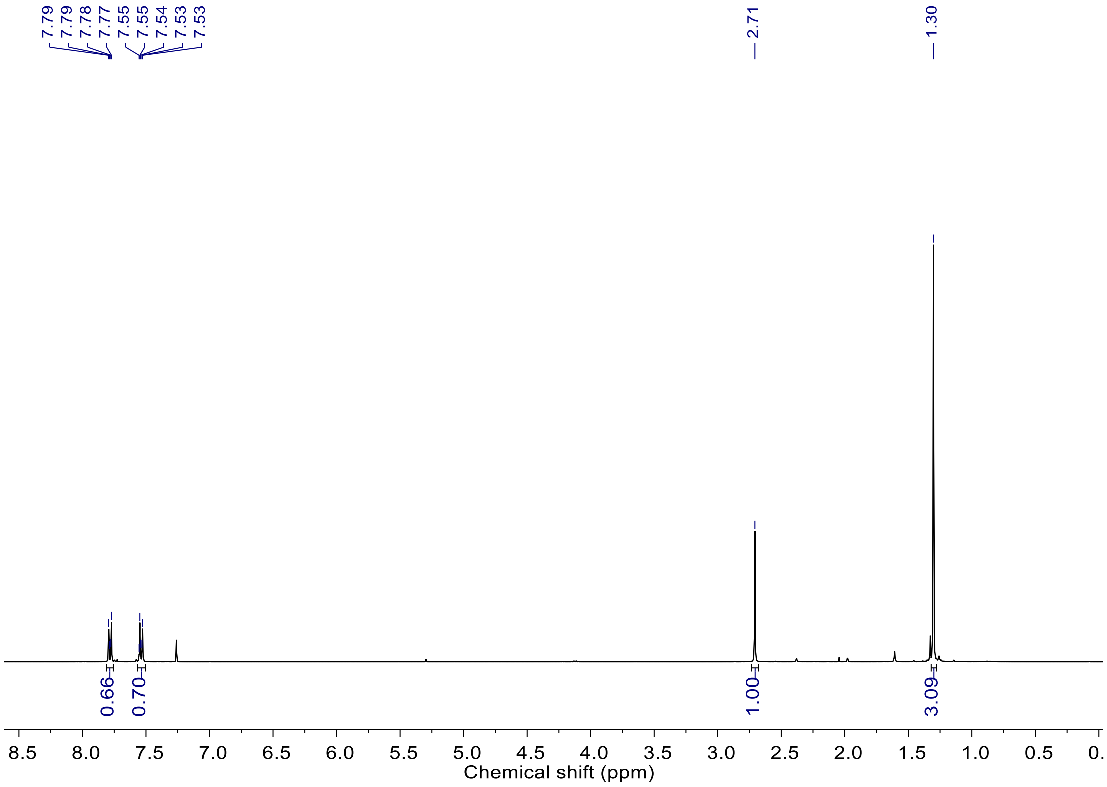

Fig. S89 ^{1}H NMR spectrum of **6 Chiral** in $CDCl_3$.

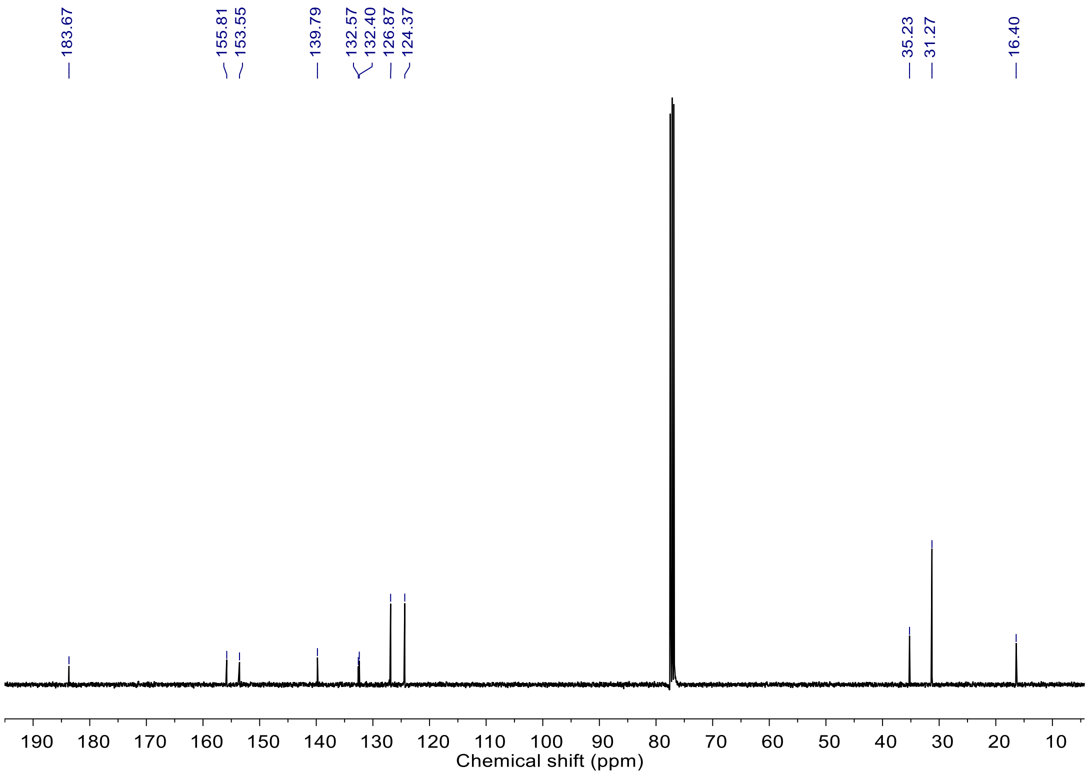

Fig. S90 ^{13}C NMR spectrum of **6 Chiral** in $CDCl_3$.

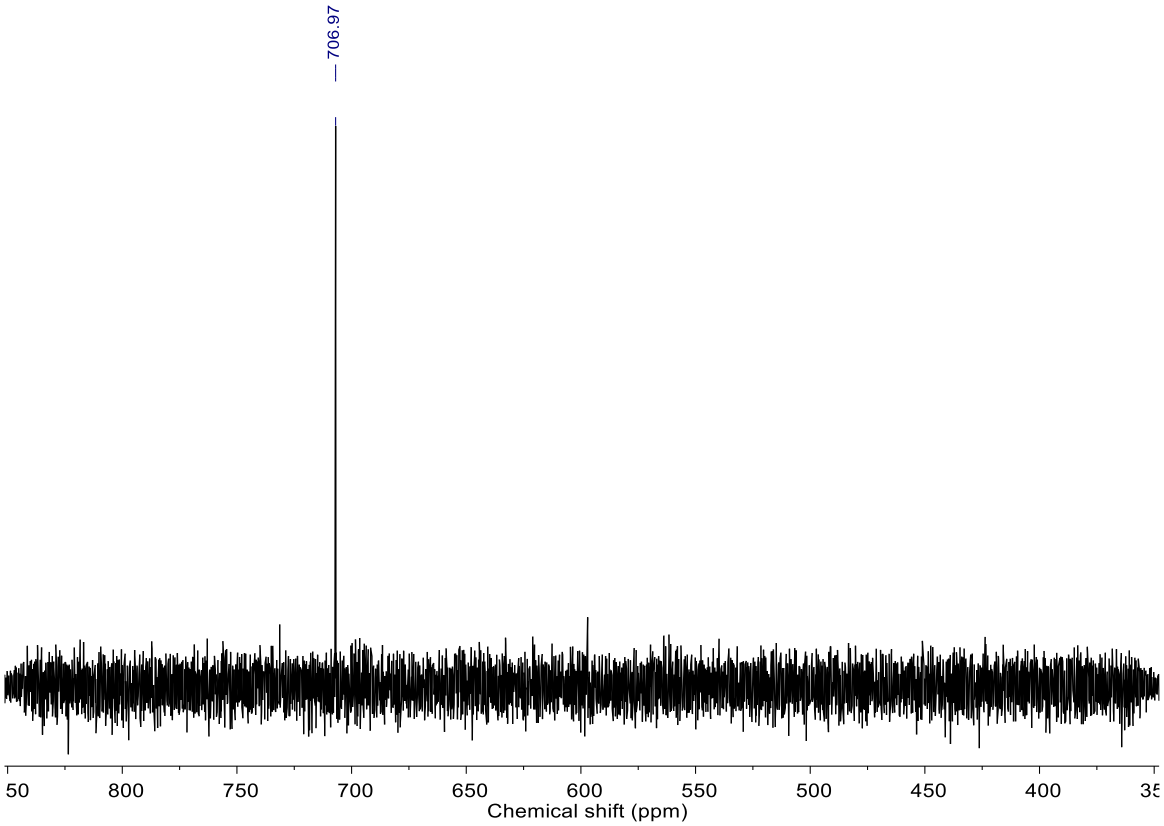

Fig. S91 ^{77}Se NMR spectrum of **6 Chiral** in $CDCl_3$.
